# Discovery and In Vitro Reconstitution of Closoxazole Biosynthesis from *Pyxidicoccus fallax*

**DOI:** 10.1002/cbic.202500126

**Published:** 2025-04-28

**Authors:** Lucia Lernoud, Rimonda Jakob, Lars Warnick, Marie Luisa Marx, Lea Winand

**Affiliations:** ^1^ Department of Biochemical and Chemical Engineering Laboratory of Technical Biology TU Dortmund University Emil‐Figge‐Str. 66 44227 Dortmund Germany

**Keywords:** benzoxazoles, biocatalysis, condensing amidohydrolase, heterocycles, natural products

## Abstract

Benzoxazoles are important structural components of both bioactive natural products and pharmaceutical active ingredients. In this study, a putative benzoxazole gene cluster originating from the myxobacterium *Pyxidicoccus fallax* is investigated. This gene cluster is found to confer the ability for production of closoxazoles, which were recently discovered in the anaerobic bacterium *Clostridium cavendishii*. To obtain further insights into the biosynthetic mechanism, the required key enzymes are subjected to in vitro studies. Notably, significant differences to the biosynthetic pathway in *C. cavendishii* are observed. First, the condensing amidohydrolase uses an unstable ester as substrate and, thus, establishes a C—N bond for benzoxazole formation. In contrast, the homolog from *C. cavendishii* is thought to use an amide substrate. Second, both AMP ligases encoded in this pathway attach a third aryl carboxylic acid building block to the benzoxazole intermediate, but these enzymes exhibit different regioselectivities. This facilitates the production of closoxazole A and B but also gives access to new derivatives in which a third building block is linked to the phenolic amine of the benzoxazole. The substrate flexibility of these enzymes allows us to introduce other building blocks into the biosynthetic pathway and thus expand the structural diversity of benzoxazole‐containing natural products.

## Graphical abstract

The myxobacterium *Pyxidicoccus fallax* is found to be a producer of benzoxazole‐containing natural products named closoxazoles. In vitro studies with the biosynthetic enzymes reveal new insights into the reaction mechanism for closoxazole formation. The biosynthetic plasticity enables the production of new closoxazoles with different substitution patterns, increasing the structural diversity of benzoxazole‐containing natural products.

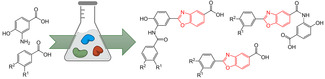

## 1 Introduction

Benzoxazole heterocycles can be found in a variety of biologically active compounds.^[^ ^1^ ^]^ For instance, the muscle relaxant chlorzoxazone, the transthyretin stabilizer tafamidis, the insomnia medication suvorexant, or pemafibrate, used for treatment of hyperlipidaemia, are currently approved drugs containing a benzoxazole motif.^[^ ^2^ ^]^ In addition, benzoxazoles are a characteristic feature of various natural products as exemplified by the antibiotics A‐33853, boxazomycin, calcimycin, and caboxamycin, or the anticarcinogenic compounds nakijinol, nataxazole, and norcabenzoxazole.^[^ ^3^ ^]^

**Figure 1:**
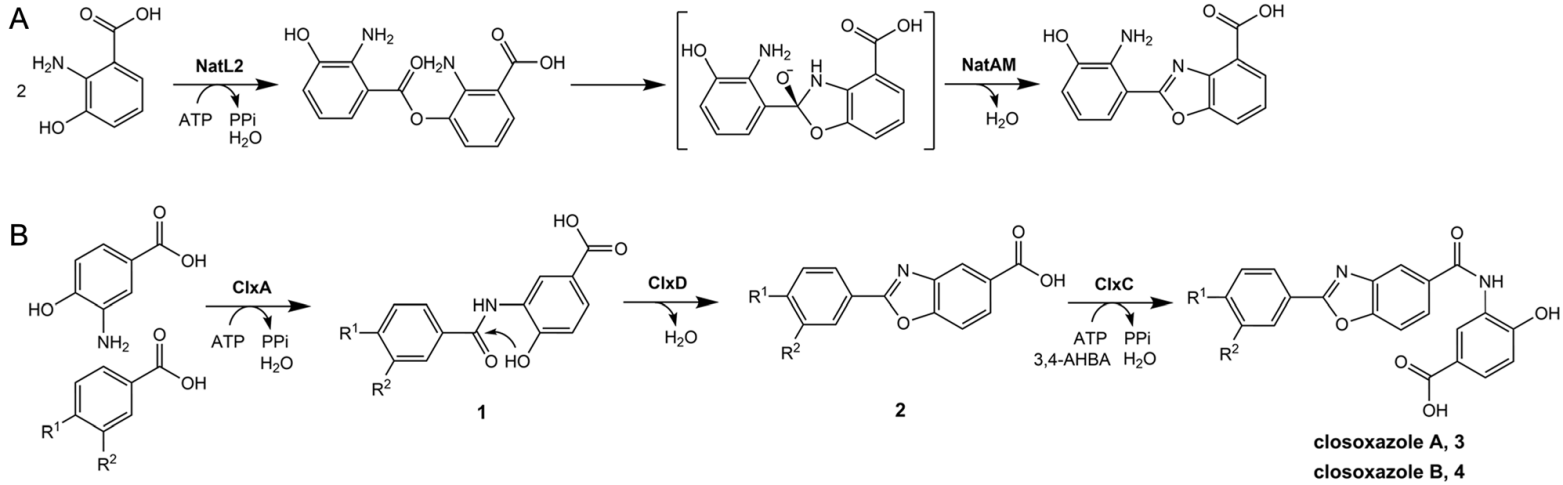
A) Enzymatic benzoxazole formation during nataxazole biosynthesis. B) Biosynthetic pathway of closoxazoles from *C. cavendishii* DSM 21758. Closoxazole A: R^1^ = OH, R^2^ = NH_2_. Closoxazole B: R^1^ = NH_2_, R^2^ = H.^[^ ^4^, ^9^ ^]^

For a long time, little was known about how benzoxazoles are produced in nature. The elucidation of the nataxazole biosynthesis from *Streptomyces* sp. Tü 6167 shed light on the biocatalytic origin of these heterocycles.^[^ ^4^ ^]^ The key enzymes of the nataxazole biosynthetic pathway, the AMP ligase NatL2 and the amidohydrolase NatAM, are sufficient to produce benzoxazoles from aryl carboxylic acid building blocks. The AMP ligase NatL2 first activates 3‐hydroxyanthranilic acid (3‐HAA) via adenylation and subsequently performs a dimerization with a second 3‐HAA building block to an ester intermediate (**Figure** **1**A).

Afterward, the amidohydrolase NatAM catalyzes an intramolecular cyclocondensation to form the benzoxazole heterocycle. In a zinc‐dependent mechanism, this enzyme generates a hemiorthoamide from the ester intermediate and eliminates water to yield the benzoxazole. Homologs of the NatAM enzyme can also be found in biosynthetic pathways of other benzoxazole‐containing natural products, e.g., antibiotic A‐33853 or caboxamycin.^[^ ^5^, ^6^ ^]^ In addition, a similar heterocyclization reaction has been reported for the condensing amidohydrolase MxcM, which performs an intramolecular condensation of the ß‐aminoethyl amide of the non‐ribosomal peptide myxochelin B to synthesize an imidazoline moiety.^[^ ^7^, ^8^ ^]^

In 2022, the anaerobic bacterium *Clostridium cavendishii* DSM 21758 was found to produce benzoxazole‐containing natural products named closoxazole A and B, in which the benzoxazole moiety also is generated by the activity of a condensing amidohydrolase.^[^ ^9^ ^]^ The amidohydrolase encoded in this pathway only has low sequence homology to the aforementioned condensing amidohydrolases (18.4% sequence similarity to NatAM, 18.3% sequence similarity to MxcM).

The biosynthesis of closoxazoles starts from the precursor 3‐amino‐4‐hydroxybenzoic acid (3,4‐AHBA), which is produced by a putative 2‐amino‐3,7‐dideoxy‐d‐threo‐hept‐6‐ulosonate synthase (ClxE) and a putative 3,4‐AHBA synthase (ClxB) from l‐aspartate‐4‐semialdehyde and dihydroxyacetone phosphate. Subsequently, the AMP ligase ClxA adenylates 3,4‐AHBA and ligates it with another 3,4‐AHBA unit (Figure 1B). The ligation product (**1**) is then subjected to the intramolecular condensation reaction catalyzed by the amidohydrolase ClxD to form the benzoxazole heterocycle (**2**). Interestingly, experimental data indicate that the amidohydrolase accepts an amide as substrate and not an ester, as reported for the NatAM enzyme.^[^ ^9^ ^]^ Consequently, the amidohydrolase ClxD is meant to form a C—O bond during the cyclocondensation reaction. The second AMP ligase encoded in the biosynthetic gene cluster, ClxC, attaches a third 3,4‐AHBA building block to the carboxy function of the benzoxazole intermediate to finally yield closoxazole A (**3**). Additionally, it has been demonstrated that the closoxazole biosynthesis enzymes exhibit a certain substrate flexibility. In *C. cavendishii* DSM 21758, 4‐aminobenzoic acid (4‐ABA) can be used as an alternative building block to 3,4‐AHBA, leading to the synthesis of closoxazole B (**4**). In a precursor‐directed biosynthesis experiment using a recombinant *E. coli* strain fed with 3,5‐dichlorobenzoic acid, an analog of the pharmaceutical drug tafamidis was produced.^[^ ^9^ ^]^

A bioinformatic analysis indicated that gene clusters, which are related to the known benzoxazole biosynthetic gene clusters (BGC), occur in numerous bacteria from different phyla.^[^ ^9^ ^]^ In this study, we investigated a putative benzoxazole BGC originating from the myxobacterium *Pyxidicoccus fallax* DSM 14698. The two AMP ligases and the condensing amidohydrolase encoded in this gene cluster were heterologously expressed in *Escherichia coli* and used for in vitro reconstitution of the benzoxazole pathway.

## 2 Results and Discussion

### 2.1 In Silico Analysis of *Pyxidicoccus fallax* DSM 14698

**Figure 2:**
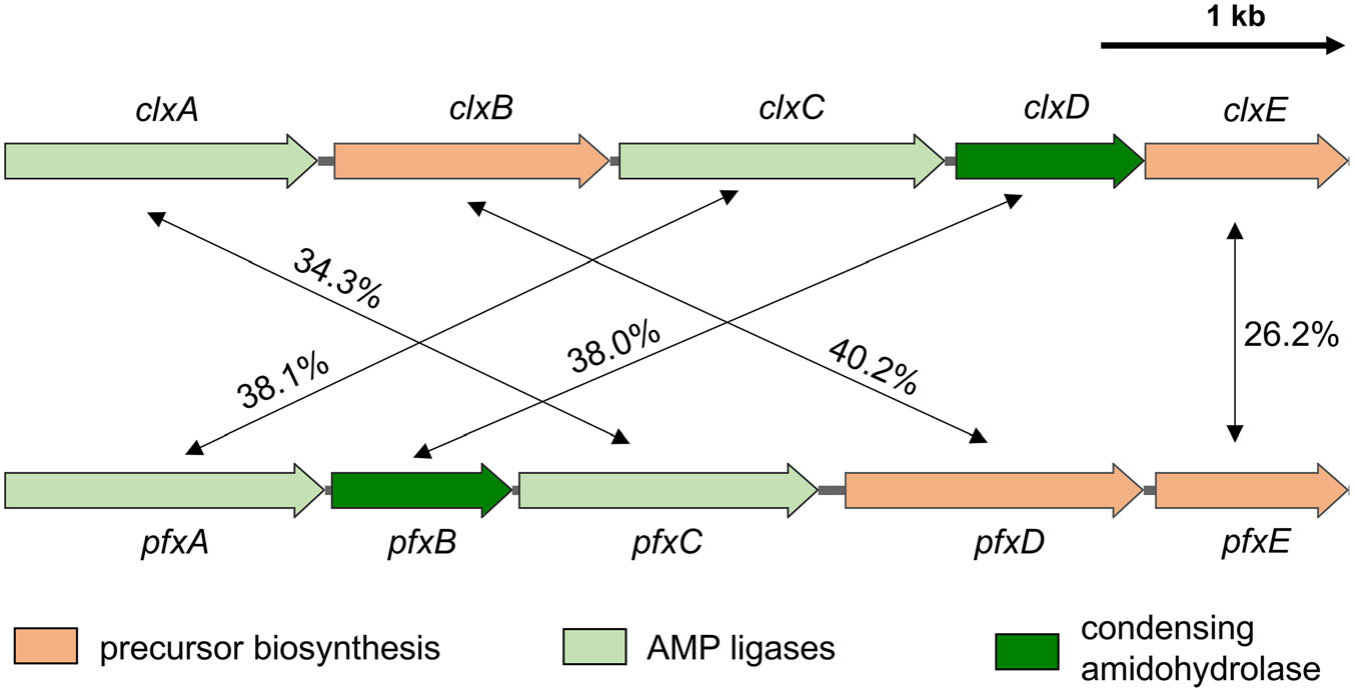
Comparison of the closoxazole biosynthetic gene cluster from *C. cavendishii* DSM 21758 (top) with its homolog from the myxobacterium *P. fallax* DSM 14698 (bottom).

A protein blast analysis of the condensing amidohydrolase ClxD revealed a multitude of homologous enzymes in various bacterial phyla, e.g., bacillota, actinomycetota, or myxococcota. The putative condensing amidohydrolase‐encoding genes originating from myxobacteria attracted our special interest. To date, no benzoxazole‐containing natural products have been identified in myxobacteria. In silico analysis of the *P. fallax* DSM 14698 genome (submitted GenBank assembly: GCA_012933655) revealed a set of five genes with homologies to the closoxazole BGC of *C. cavendishii* (**Figure** **2**). However, the sequence similarities are rather low and the arrangement of the genes in the two clusters differs. The enzymes encoded by *pfxD* (locus HG543_RS13895) and *pfxE* (locus HG543_RS13900) share 40.2 and 26.6% homology to ClxB and ClxE on the amino acid level and thus are assumed to be involved in precursor biosynthesis. The gene *pfxA* (locus HG543_RS13880) encodes an AMP ligase that has the highest homology to ClxC (38.1% similarity), whereas *pfxC* (locus HG543_RS13890) encodes another AMP ligase that is homologous to ClxA (40.2% similarity). Gene *pfxB* (locus HG543_RS13885) encodes an amidohydrolase (38.0% similarity to ClxD) that presumably is involved in heterocycle formation.

### 2.2 Cultivation and Extraction of *P. fallax*

Due to the low sequence homology of the biosynthetic enzymes encoded in the two BGCs of *P. fallax* and *C. cavendishii*, our initial aim was to examine whether *P. fallax* is capable of producing closoxazoles, or if other benzoxazole‐containing natural products are produced. For that purpose, *P. fallax* DSM 14698 was cultivated for 12 days in VY/2 medium. Via LC–MS analysis, we were able to detect trace amounts of the mass of closoxazole A (**3**) in the bacterial raw extract. This result implies that the putative closoxazole gene cluster from *P. fallax* is in fact responsible for the biosynthesis of closoxazoles. To trigger the production of **3**, we also supplemented the building block 3,4‐AHBA to *P. fallax* cultures. In the extract of these cultures, the mass of **3** was present in higher intensity and masses corresponding to the ligation product (**1**) and the benzoxazole (**2**) were also detected (Figure S1, Supporting Information). The MS/MS fragmentation pattern is consistent with the fragmentation published for **3** (Figure S2, Supporting Information).^[^ ^9^ ^]^ The mass of closoxazole B (**4**) was not present in the bacterial raw extracts. Additional feeding of the required building block 4‐ABA also did not trigger the production of **4**. It is not unlikely that the closoxazole biosynthetic enzymes of *P. fallax* have a substrate specificity that differs from that of their homologs originating from *C. cavendishii*. Notably, in the benzoxazole biosynthetic pathways of the natural products caboxamycin, nataxazole, and antibiotic A‐33853, the alternative building blocks salicylic acid, 6‐methylsalicylic acid, or 3‐hydroxypicolinic acid are recruited, respectively.^[^ ^5^, ^6^, ^10^ ^]^ For that reason, *P. fallax* might also utilize other aryl carboxylic acids as building blocks for closoxazole biosynthesis. Analysis of the *P. fallax* DSM 14698 genome indicated that the genome encodes an anthranilate synthase, a kynureninase and a 2,3‐dihydro‐2,3‐dihydroxybenzoate dehydrogenase. Thus, *P. fallax* most likely is a native producer of anthranilic acid, 3‐HAA, and 2,3‐dihydroxybenzoic acid. However, inspection of the bacterial raw extracts via LC–MS did not reveal any compounds that might correspond to closoxazole derivatives or the respective biosynthetic intermediates incorporating aryl carboxylic acids other than 3,4‐AHBA.

### 2.3 In Vitro Reconstitution of the Closoxazole Biosynthetic Pathway from *P. fallax*

**Figure 3:**
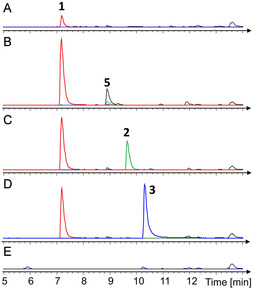
In vitro reconstitution of the putative closoxazole pathway from *P. fallax* DSM 14698 using 3,4‐AHBA as substrate. LC–MS chromatograms of reactions including A) PfxA, B) PfxC, C) PfxC and PfxB, D) PfxC, PfxB, and PfxA. E) Negative control without enzymes. Black: BPC; Red: EIC of **1** (*m/z* 289.08); Green: EIC of **2** (*m/z* 271.07); Blue: EIC of **3** (*m/z* 406.10).

At this point, there was substantial evidence that the BGC identified in *P. fallax* mediates the ability to produce closoxazoles. However, it was not yet clear whether the biosyntheses in *C. cavendishii* and *P. fallax* rely on similar mechanisms. To date, no in vitro experiments have been conducted to investigate the closoxazole biosynthetic pathway in detail. With this intent, the two AMP ligases PfxA and PfxC and the amidohydrolase PfxB were heterologously produced in *E. coli* using the pET28a(+) plasmid as expression vector (Figure S3–S5, Supporting Information). Each enzyme was equipped with an N‐terminal hexahistidine tag to enable protein purification via Ni‐NTA affinity chromatography. The success of the protein purification was verified via SDS‐PAGE (Figure S6–S8, Supporting Information). To reconstitute the closoxazole pathway in vitro using the Pfx enzymes, we tested different enzyme combinations with 3,4‐AHBA as substrate (**Figure** **3**).

Initially, the formation of the ligation product of two 3,4‐AHBA units using the AMP ligases was investigated. After incubation of PfxA or PfxC in the presence of 3,4‐AHBA and ATP, the mass of the ligation product (**1**, *m/z* 289.08) was observed in both reactions. Notably, the significantly larger peak area in the presence of PfxC indicates that this enzyme is primarily responsible for catalyzing the initial ligation reaction. We also observed another compound (**5**) with a *m/z* value of 424.11 in the PfxC reaction that presumably corresponds to a ligation product consisting of three 3,4‐AHBA building blocks. If the amidohydrolase PfxB is added to the reaction mixture containing PfxC and 3,4‐AHBA, a new peak is generated with a *m/z* value of 271.07 corresponding to the benzoxazole intermediate (**2**). Concludingly, the amidohydrolase catalyzes an intramolecular condensation of the ligation product generated by PfxC to form the benzoxazole heterocycle. If both AMP ligases and the amidohydrolase are incubated in the presence of 3,4‐AHBA and ATP, a new peak arises at a retention time of 10.3 min. The mass (*m/z* 406.10) as well as the MS/MS fragmentation pattern (Figure S9, Supporting Information) of this peak is consistent with the published data for **3**.^[^ ^9^ ^]^ After upscaling the in vitro reaction, this compound was purified and analyzed by NMR, which unequivocally confirmed that the chemical structure is identical to closoxazole A (**3**) (Figure S10, S11, Supporting Information).

### 2.4 Detailed Investigation of the Reaction Pathway

**Figure 4:**
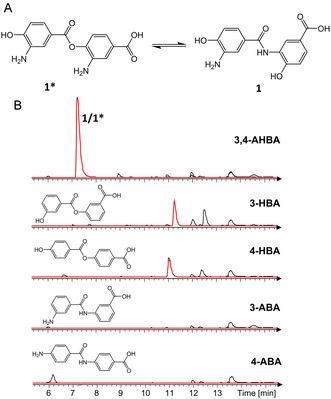
A) Predicted spontaneous ester amide rearrangement in aqueous solution. B) LC–MS chromatograms of the ligation product formation using the AMP ligase PfxC and the aryl carboxylic acids 3,4‐AHBA (**1**/1*; *m/z* 289.08), 3–HBA (*m/z* 259.06), 4‐HBA (*m/z* 259.06), 3‐ABA (*m/z* 257.09), or 4‐ABA (*m/z* 257.09) as substrates. Black: BPC; Red: EICs of the respective ligation products. Negative controls are shown in Figure S18, Supporting Information.

For the reaction mechanism of the benzoxazole formation in the closoxazole biosynthesis from *C. cavendishii*, a substantial difference to the reaction pathway of the nataxazole biosynthesis was reported. The amidohydrolase NatAM utilizes an ester as substrate, thus generating a C—N bond during the cyclocondensation.^[^ ^4^ ^]^ On the other hand, ClxD is supposed to synthesize the benzoxazole via a C—O bond.^[^ ^9^ ^]^ Because of this discrepancy, our aim was to determine the identity of the substrate converted by the condensing amidohydrolase PfxB. For this purpose, we cultured the PfxC expression strain *E. coli* BL21(DE3): pET28a‐*pfxC* under supplementation of 3,4‐AHBA and isolated the ligation product (**1**) from the bacterial raw extract using a semipreparative HPLC (Figure S12, Supporting Information). NMR analyses of the purified compound revealed that the two 3,4‐AHBA monomers are linked via an amide bond (Table S3, Figure S13–S17, Supporting Information). Afterward, the purified ligation product (**1**) was used as substrate in an in vitro reaction with the amidohydrolase PfxB, but no product formation was obtained. Even after the addition of PfxC and ATP to this reaction mixture no benzoxazole was synthesized, thus excluding any enzyme‐enzyme interactions. Our next attempt was to reproduce the feeding experiment conducted by Horch et al.^[^ ^9^ ^]^ While feeding the amide (**1**) to the ClxD expression strain led to the formation of **2**, we again did not observe any evidence for enzymatic activity analyzing the bacterial raw extract of our PfxB expression strain fed with **1**. These results might indicate that an ester (1*) is the true biosynthetic intermediate used as substrate by the amidohydrolase PfxB, and not the amide (**1**), as it was reported for ClxD. In this scenario, the AMP ligase PfxC from *P. fallax* links the two 3,4‐AHBA building blocks via an ester bond. However, the ester is likely unstable in aqueous environment, resulting in an ester‐amide rearrangement (**Figure** **4**A). A similar situation was demonstrated for the reaction pathway catalyzed by NatL2 and NatAM.^[^ ^4^ ^]^ To obtain further evidence to support this hypothesis, the AMP ligase PfxC was incubated with 3‐hydroxybenzoic acid (3‐HBA) or 4‐hydroxybenzoic acid (4‐HBA) and the reaction mixtures were screened for the respective dimeric structures. As these aryl carboxylic acids lack an amino function, the ligation can only occur via an ester bond. The formation of the expected ligation products was successfully confirmed by LC–MS measurement (Figure 4B). On the other hand, when 3‐aminobenzoic acid (3‐ABA) or 4‐ABA are used as substrates, only minor traces of the respective ligation products were formed. This result validates that PfxC is able to ligate two aryl carboxylic acids via an ester bond. Using the ester 1* as substrate, the amidohydrolase PfxB then generates the benzoxazole heterocycle by establishing a C—N bond, as reported for the amidohydrolase NatAM.

**Figure 5:**
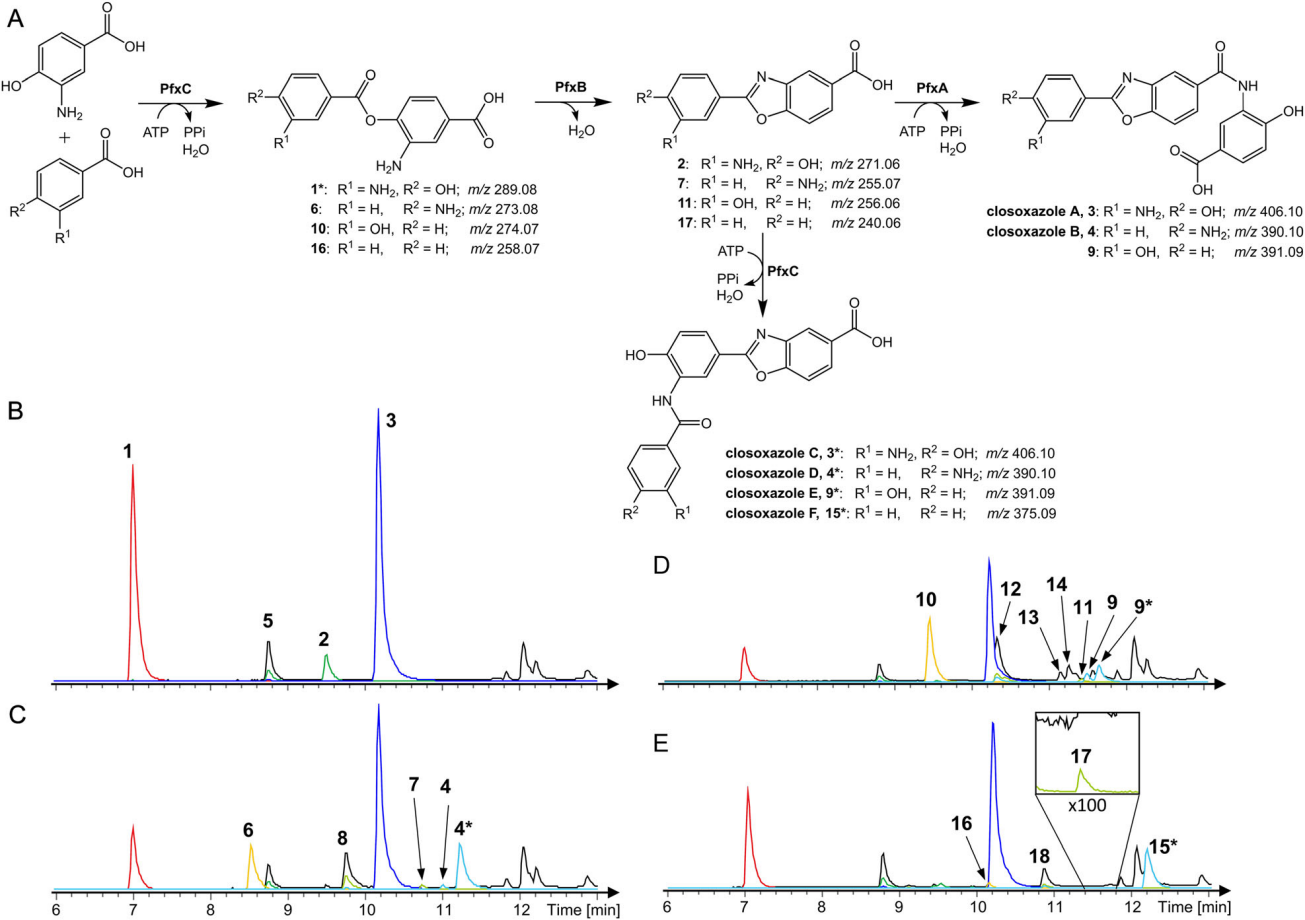
A) Reaction pathway for closoxazole biosynthesis using the enzymes PfxA, PfxB, and PfxC originating from *P. fallax* DSM 14698. Aryl carboxylic acids other that 3,4‐AHBA can be channeled into the reaction pathway for production of new closoxazole derivatives. LC–MS chromatograms of in vitro reactions using PfxA, PfxB, and PfxC and the substrates B) 3,4‐AHBA, C) 3,4‐AHBA and 4‐ABA, D) 3,4‐AHBA and 3‐HBA, or E) 3,4‐AHBA and BA. Black: BPC; Colored: EICs of the respective closoxazole derivatives and the biosynthetic intermediates. Negative controls are shown in Figure S40, Supporting Information.

Afterward, we examined the second ligation reaction in more detail. We engineered a recombinant *E. coli* strain that expresses both the AMP ligase PfxC and the amidohydrolase PfxB from *P. fallax* (Figure S19, Supporting Information). This expression strain was used for production of the benzoxazole intermediate (**2**) starting from 3,4‐AHBA (Figure S20, Supporting Information). The benzoxazole (**2**) was purified from the bacterial raw extract and its identity was verified by NMR (Table S4, Figure S21–S25, Supporting Information). Subsequently, we performed further in vitro reactions in which the enzymes PfxA or PfxC, respectively, were incubated in presence of the benzoxazole (**2**), 3,4‐AHBA and ATP (Figure S26, Supporting Information). LC–MS analyses of these reaction mixtures revealed that significant amounts of closoxazole A (**3**) were formed in the PfxA reaction, while the peak area of **2** was decreased. Using PfxC as biocatalyst, we also obtained a compound with an *m/z* value of 406.10, but at a retention time of 10.5 min. In contrast, **3** elutes after a retention time of 10.3 min. Based on LC–MS/MS analyses (Figure S27, Supporting Information), the compound generated by PfxC is expected to be a constitutional isomer of **3** (herein referred to as 3*) where the third 3,4‐AHBA building block is attached to the phenolic amine of **2**. When analyzing the bacterial raw extract of the PfxC and PfxB expression strain, we also observed significant amounts of the putative constitutional isomer 3* (Figure S20, Supporting Information), which we purified using semi‐preparative HPLC. NMR spectroscopic analyses confirmed the structural identity of 3*, which is hereinafter termed closoxazole C (Table S5, Figure S28–S32, Supporting Information). From this, it can be concluded that both AMP ligases PfxA and PfxC are able to attach a third 3,4‐AHBA building block to the benzoxazole (**2**), but with different regioselectivities (**Figure** **5**A).

### 2.5 Biocatalytic Synthesis of Closoxazole Derivatives

Interestingly, we detected a new peak with a mass that might correspond to closoxazole B (**4**, *m/z* 390.10) in the raw extract of *E. coli* BL21(DE3): pET28a‐*pfxBC* fed with 3,4‐AHBA (Figure S20, Supporting Information). *E. coli* naturally has the ability to synthesize 4‐ABA via the shikimate pathway.^[^ ^11^ ^]^ We therefore hypothesized that 4‐ABA can be introduced as an alternative building block in the truncated closoxazole pathway. To stimulate the production of this compound, the *E. coli* expression strain was cultivated in the presence of both 3,4‐AHBA and 4‐ABA. This led to a significantly increased peak intensity in the LC–MS chromatogram of the bacterial raw extract (Figure S33, Supporting Information). A comparison of the MS/MS fragmentation pattern with the one previously published indicated that this compound is not closoxazole B (**4**) (Figure S34, Supporting Information). Instead, the fragmentation is analogous to that of the constitutional isomer 3*. Upon HPLC purification, NMR analysis confirmed that a new closoxazole derivative was produced (designated as closoxazole D or 4*), in which the 4‐ABA building block is linked to the phenolic amine of **2** (Table S6, Figure S35–S39, Supporting Information). Subsequently, we performed in vitro reactions where the enzymes PfxA, PfxB, and PfxC are incubated in presence of both 3,4‐AHBA and 4‐ABA. In these reactions, we were able to detect compounds **1**, **2**, and **3** along with closoxazole D (4*) as well as other masses that can be attributed to the ligation product of 3,4‐AHBA and 4‐ABA (**6**, *m/z* 273.09) and the corresponding benzoxazole (**7**, *m/z* 255.08) (Figure 5C).

In addition, a second peak with an *m/z* of 390.10 was detected, but at a retention time that differed from 4*. We assumed this compound to be closoxazole B (**4**), which was supported by the MS/MS fragmentation pattern (Figure S41, Supporting Information). The mass (*m/z* 408.12) and MS/MS fragmentation of compound **8** corresponds to a ligation product consisting of one 4‐ABA and two 3,4‐AHBA building blocks (Figure S42, Supporting Information).

To investigate if aryl carboxylic acids other than 3,4‐AHBA and 4‐ABA can also be incorporated in the closoxazole biosynthesis, we prepared further in vitro reactions in which 3‐HBA or benzoic acid (BA) were used as alternative building blocks. In the reaction with 3‐HBA and 3,4‐AHBA, two peaks with an *m/z* value of 391.10 were observed after retention times of 11.5 and 11.6 min, respectively (Figure 5D). The mass of these compounds fits to the predicted closoxazole derivatives incorporating a 3‐HBA building block. Taking the MS/MS fragmentation of the compound eluting after 11.6 min (designated as 9*) into account (Figure S43, Supporting Information), we assume that the 3‐HBA is attached to the phenolic amine of **2**. According to our previous findings, this reaction is thought to be catalyzed by PfxC. The chemical structures of this compound, named closoxazole E (9*), as well as of the benzoxazole intermediate **11**, were verified after in vivo production in the PfxC and PfxB expression strain (Table S7–S8, Figure S44–S54, Supporting Information). The fragmentation of **9** rather indicates that this compound is made from a benzoxazole that consists of a 3‐HBA and a 3,4‐AHBA building block via attachment of another 3,4‐AHBA to its carboxy function, most likely mediated by the AMP ligase PfxA. The *m/z* values of the 3‐HBA and 3,4‐AHBA ligation product (**10**, *m/z* 274.07) as well as for the corresponding benzoxazole derivative (**11**, *m/z* 256.06) were also present in the enzymatic reaction. In addition, we identified three other peaks with *m/z* values that might correspond to a ligation product made of two 3,4‐AHBA and one 3‐HBA building block (**12**, *m/z* 409.10), a ligation product made of two 3‐HBA units (**13**, *m/z* 259.06), and a ligation product that consists of one 3,4‐AHBA and two 3‐HBA building blocks (**14**, *m/z* 394.09) (Figure S55, Supporting Information).

When BA is used as a substrate along with 3,4‐AHBA in reactions including PfxA, PfxB, and PfxC, only one peak with a mass of a putative closoxazole derivative was produced (15*, *m/z* 375.09) (Figure 5E). Feeding *E. coli* BL21(DE3): pET28a‐*pfxBC* with both 3,4‐AHBA and BA led to a compound with the same mass, retention time and MS/MS fragmentation pattern (Figure S56–S57, Supporting Information). NMR analyses revealed that this compound is another closoxazole derivative, designated as closoxazole F (15*), where the BA building block is attached to the phenolic amine (Table S9, Figure S58–S62, Supporting Information). We also observed peaks that might correspond to a 3,4‐AHBA and BA ligation product (**16**, *m/z* 258.07) and the respective benzoxazole derivative (**17**, *m/z* 240.06). However, the low peak intensity indicates that BA is not very well accepted by PfxC for the initial ligation reaction. The additional peak (**18**) at a retention time of 10.9 min has an *m/z* value 393.11 and can be attributed to a ligation product consisting of one BA and two 3,4‐AHBA building blocks (Figure S63, Supporting Information).

With the newly acquired knowledge on the substrate tolerance of the closoxazole biosynthetic enzymes, we reinvestigated the bacterial raw extract of *P. fallax* DSM 14698 by LC–MS for the presence of the corresponding derivatives, but did not uncover any evidence for their production.

## 3 Conclusion

Condensing amidohydrolases are a group of enzymes that catalyze intramolecular cyclocondensations for heterocycle formation. These enzymes act independent of (organic) cofactors and were shown to convert non‐natural substrates.^[^ ^4^, ^7^, ^12^ ^]^ For the condensing amidohydrolase MxcM, a good storage stability and a high tolerance to organic solvents have also been documented.^[^ ^7^, ^8^ ^]^ These characteristics make condensing amidohydrolases promising candidates for use in heterocyclic chemistry. In 2022, the condensing amidohydrolase ClxD was reported to synthesize a benzoxazole heterocycle during the biosynthetic pathway of the natural products closoxazole A and B in *Clostridium cavendishii*.^[^ ^9^ ^]^ Interestingly, putative benzoxazole producing gene clusters including condensing amidohydrolase encoding genes are present in various strains of different bacterial phyla. In this study, we investigated the benzoxazole gene cluster of the myxobacterium *P. fallax*. After cultivation of *P. fallax* and analysis of the bacterial crude extracts, we demonstrated that this gene cluster confers the ability for closoxazole A biosynthesis.

Subsequently, the condensing amidohydrolase PfxB and the AMP ligases PfxA and PfxC encoded in the closoxazole gene cluster of *P. fallax* were heterologously produced in *E. coli* and investigated in vitro. Initially, the AMP ligase PfxC ligates two 3,4‐AHBA building blocks. Our data indicate that this enzyme establishes an ester bond to connect the 3,4‐AHBA units, and not an amide, as proposed for the closoxazole pathway of *C. cavendishii*.^[^ ^9^ ^]^ However, we assume that the ester is unstable in aqueous solution and can spontaneously transform into the amide. A similar mechanism was observed in the nataxazole biosynthesis.^[^ ^4^ ^]^ As the amide is not accepted as a substrate by the condensing amidohydrolase PfxB, the benzoxazole formation occurs via the ester intermediate. The AMP ligase PfxA then catalyzes the ligation of another 3,4‐AHBA building block to synthesize closoxazole A (**3**). Interestingly, the AMP ligase PfxC can also link a third building block to the benzoxazole intermediate (**2**), but with different regioselectivity, resulting in constitutional isomers of the known closoxazoles A and B.

Additionally, we demonstrated that, using the Pfx enzymes, also other aryl carboxylic acids than 3,4‐AHBA or 4‐ABA can be channeled into the closoxazole pathway. This way, new derivatives containing 3‐HBA or BA building blocks were produced, thus further expanding the structural diversity of benzoxazole‐containing natural products. In conclusion, the Pfx enzymes represent valuable candidates for the biocatalytic synthesis of benzoxazoles with variable substitution patterns. For that purpose, the substrate scope and kinetic parameters of the enzymes deserve further investigation in future.

## 4 Experimental Section

4.1

4.1.1

##### Strains, Nucleic Acids and Plasmids

The bacterial strains and plasmids used in this study can be found in Table S1, Supporting Information. The genes *pfxA, pfxC* and the gene *pfxB* (codon optimized for *E. coli*) were synthesized by Life Technologies GmbH (Thermo Scientific, see Supporting Information).

##### Cultivation of *P. fallax* DSM 14698

*P. fallax* DSM 14698 was cultivated in either solidified or liquid VY/2 medium (5 g L^−1^ Baker´s yeast, 1.36 g L^−1^ CaCl_2_ × 2 H_2_O, 0.5 mg L^−1^ vitamin B12; pH 7.4). For closoxazole production analysis, 200 mL VY/2 cultures were cultivated for 12 days at 30 °C and 130 rpm. Some cultures were supplemented with 50 mg L^−1^ of 3,4‐AHBA or 4‐ABA to trigger closoxazole production. At the end of cultivation, 30 g L^−1^ of the adsorber resin Amberlite XAD7HP (Sigma‐Aldrich) were added and the cultures were incubated for two additional hours. Afterward, the adsorber resin was recovered by filtration, washed with water, and adsorbed metabolites were eluted with 100 mL methanol (MeOH). The extracts were concentrated with a rotary evaporator (Heidolph) and analyzed via LC–MS.

##### Growth Conditions of *E. coli* and Nucleic Acid Extraction

*E. coli* TOP10 strains were cultivated either in liquid or solidified LB medium (10 g L^−1^ tryptone, 10 g L^−1^ NaCl, 5 g L^−1^ yeast extract; pH 7). *E. coli* BL21(DE3) strains were cultivated either on solidified LB Medium or in liquid TB medium (12 g L^−1^ tryptone/peptone, 24 g L^−1^ yeast extract, 4 mL L^−1^ glycerol, 0.17 M KH_2_PO_4_, 0.72 M K_2_HPO_4_; pH 7). *E. coli* liquid cultures were incubated at 180 rpm and, if not indicated differently, at a temperature of 37 °C. If required, 50 mg L^−1^ kanamycin were used as selection marker. Plasmid DNA was purified from *E. coli* using the NucleoSpin Plasmid (NoLid) Mini Kit (Macherey‐Nagel). DNA entrapped in agarose gel was purified using the NucleoSpin Gel and PCR Clean‐up Mini Kit (Macherey‐Nagel).

##### General Cloning Procedure

Plasmids were constructed by making use of the Gibson Assembly method^[^ ^13^ ^]^ or circular polymerase extension cloning (CPEC).^[^ ^14^ ^]^ Insert DNA was amplified via overhang PCR using the Phusion High‐Fidelity DNA‐Polymerase (Thermo Scientific). Plasmid linearization was performed with the FastDigest restriction enzymes (Thermo Scientific). Linearized plasmids were dephosphorylated with FastAP alkaline phosphatase (Thermo Scientific) to avoid recircularization. For Gibson assembly, 5 μL 2× GeneArt Gibson Assembly HiFi Master Mix (Invitrogen) were mixed with 50 ng linearized plasmid in a total volume of 10 μL. The DNA inserts amplified by overhang PCR were added at insert‐to‐vector ratios of 1:1, 3:1, and 5:1. The reaction mix was incubated at 50 °C for 60 min. For CPEC, 10 μL Phusion High‐Fidelity PCR Master Mix (Thermo Scientific) were mixed with 50–100 ng linearized plasmid in a reaction volume of 20 μL. For high GC DNA, 1 μL DMSO was added. The amount of insert DNA was adjusted to insert‐to‐vector ratios of 3:1, 9:1, and 18:1. Subsequently, the assembled plasmids were used for chemical transformation of *E. coli* TOP10. Positive transformants were selected using the kanamycin resistance. The plasmid identity was verified via colony PCR and Sanger sequencing (Microsynth Seqlab).

##### Construction of Expression Plasmids

To produce the enzymes PfxA, PfxC, and PfxB as His‐tagged proteins in *E. coli*, the respective synthetic genes (see Supporting Information) were integrated into the multiple cloning site of the vector pET28a(+). For that purpose, the genes *pfxA*, *pfxB*, and *pfxC* were amplified via overhang PCR using the primer pairs P01/P02, P03/P04, or P05/P06 (Table S2, Supporting Information), respectively, to attach homologous regions required for Gibson assembly. Due to expression problems, we used a codon‐optimized gene variant of *pfxB*. Subsequently, the insert DNA was transferred into the Ecl136III restriction site of the plasmid pET28a(+).

The expression plasmid pET28a‐*pfxBC* was assembled via CPEC. For that purpose, plasmid pET28a‐*pfxB* was linearized using the restriction enzyme HindIII. The DNA insert containing T7 promoter, His‐tag coding sequence, and *pfxC* was amplified via overhang PCR using the primers P07/P08 to obtain a fragment with homologous regions for CPEC.

The plasmid identity of pET28a‐*pfxA*, pET28a‐*pfxB*, pET28a‐*pfxC*, and pET28a‐*pfxBC* was verified via colony PCR and sequencing (Microsynth Seqlab) (Figure S3–S5, Figure S18, Supporting Information). Subsequently, the plasmids were introduced into *E. coli* BL21(DE3) by chemical transformation.

##### Enzyme Production and Purification

For enzyme production, 100 mL TB medium was inoculated with well‐grown precultures of *E. coli* BL21(DE3): pET28a‐*pfxA*, *E. coli* BL21(DE3): pET28a‐*pfxB*, or *E. coli* BL21(DE3): pET28a‐*pfxC* to an OD_600_ of 0.1. The cultures were incubated at 37 °C and 180 rpm until an OD_600_ of 0.6 was reached. Subsequently, 0.5 mM IPTG was added to induce the T7 expression system. After 20 h cultivation at 20 °C, the cells were harvested by centrifugation (11.000 rpm, 5 min, 4 °C, Eppendorf Centrifuge 5804 R, rotor F‐34‐6‐38 06/18). The cell pellets were resuspended in lysis buffer (25 mM Tris, 500 mM NaCl, and 100 mL L^−1^ glycerol). Cell disruption was performed by high‐pressure homogenization at 20 kpsi (CF1 cell disruptor, Constant Systems). For removal of cell debris, the lysate was centrifuged for 30 min at 19.000 rpm and 4 °C (ThermoScientific Sorvall RC6+ centrifuge, rotor F13‐14 × 50cy). The protein of interest was then purified from the supernatant via NiNTA affinity chromatography using HisTrap FF Crude columns (Cytiva Europe) and the ÄKTA pure protein purification system (Cytiva Europe). First, the column was equilibrated with 5 column volumes (CV) binding buffer (lysis buffer with 40 mM imidazole) at a flow rate of 1 mL min^−1^. After applying the protein sample, the column was washed with 10 CV binding buffer. Subsequently, the amount of elution buffer (lysis buffer with 500 mM imidazole) was increased to 100 vol% over 10 CV to perform the protein elution. The column was flushed for further 5 CV with 100 vol% lysis buffer and washed with binding buffer (10 CV). The isolated enzyme solutions were desalted using PD‐10 desalting columns (Cytiva Europe), which were equilibrated with storage buffer (20 mM Tris, 100 mM NaCl, 100 mL L^−1^ glycerol). The protein concentrations were determined using a NanoDrop2000 spectrophotometer (Thermo Scientific), and the desalted enzymes were shock‐frozen in liquid nitrogen and stored at −80 °C. The protein samples were analyzed via SDS PAGE (Figure S6–S8, Supporting Information).

##### Enzymatic Assays

Enzyme activity assays were generally performed in Tris–HCl buffer (50 mM, pH 8) including 10 mM MgCl_2_ and 2 μM of the enzymes PfxA, PfxB, and/or PfxC. In reactions with the AMP ligases PfxA and PfxC, 2 mM ATP was added. As substrates, 3,4‐AHBA, the ligation product (**1**), the benzoxazole intermediate (**2**), or the alternative building blocks 4‐ABA, 3‐HBA, BA, 4‐HBA, or 3‐ABA were used. The substrate concentration was adjusted to 2 mM. When two substrates were used, the concentration of each substrate was 1 mM. The reactions were incubated for 3 h at 30 °C and 1.000 rpm and stopped by the addition of 2 V MeOH.

##### Heterologous Production of Closoxazoles and Its Biosynthetic Intermediates

Cultures of *E. coli* BL21(DE3): pET28a‐*pfxBC* were inoculated to an OD_600_ of 0.1 and at 37 °C and 180 rpm. At an OD_600_ of 0.6, IPTG (0.5 mM) and substrates were added. For production of **1**, **2**, and 3*, the cultures were fed 320 mg L^−1^ 3,4‐AHBA. For production of the derivatives 4*, 9*, and 15*, 50 mg L^−1^ 3,4‐AHBA and 50 mg L^−1^ of the alternative building blocks (4‐ABA, 3‐HBA or BA) were added. The cultures were incubated for 2 days at 37 °C. Product isolation was performed using the adsorber resin Amberlite XAD7HP (Sigma‐Aldrich) as described earlier. The bacterial raw extracts were analyzed via LC–MS.

For product purification, the cultivations were upscaled to a total volume of 800 mL. Compound **1** was isolated from *E. coli* BL21(DE3): pET28a‐*pfxC*, compounds **2**, 3*, 4*, 9*, **11**, and 15* were purified from the *E. coli* BL21(DE3): pET28a‐*pfxBC* crude extracts via reversed phase HPLC (Shimadzu). Closoxazole A (**3**) was purified from an upscaled in vitro reaction using PfxA, PfxB and PfxC and 3,4‐AHBA as substrate. A VP250/10 Nucleodur C_18_ Isis, 5 μm (Macherey‐Nagel) column under following conditions was used: Column oven: 30 °C. Mobile phase: Acetonitrile (ACN) and ultrapure water with 0.1 vol% trifluoroacetic acid.

For purification of **1**, **2**, 3*, 9*, and **11**, a first purification step with following flow rate and gradient specifications were used: Flow rate: 4 mL min^−1^. Gradient: 0–7 min: 10 vol% ACN; 7–21 min: 10–100 vol% ACN; 21–25 min: 100 vol% ACN; 25–27 min: 100–10 vol% ACN; 27–35 min: 10 vol% ACN. Retention times: **1**—10.2 min; **2**—13.4 min; 3*—14.3 min; 9*—17.0 min; **11**—16.9 min.

For purification of **3** and 4* following flow rate and gradient specifications were applied: Flow rate: 3 mL min^−1^ Gradient: 0–5 min 20 vol% ACN; 5–20 min: 20–50 vol% ACN; 20–21 min: 50 vol% ACN; 21–22 min: 50–20 vol% ACN; 22–25 min: 20 vol% ACN. Retention times: **3**—13.5 min; 4*—19.5 min.

For purification of 15*, following flow rate and gradient specifications were used: Flow rate: 3 mL min^−1^. Gradient: 0–5 min 20 vol% ACN; 5–20 min: 20–70 vol% ACN; 20–21 min: 70 vol% ACN; 21–22 min: 70–20 vol% ACN; 22–25 min: 20 vol% ACN. Retention time: 20.6 min.

Further purification was needed for the compounds **1**, **2**, 3*, 4*, 9*, **11** and 15*, which were all isolated from a crude extract of an *E. coli* culture.

For further purification of **1**, an isocratic washing step with a column VP250/10 Nucleodur C_18_ Sphinx, 5 μm (Macherey‐Nagel) was performed under following specifications: Column oven: 30 °C. Mobile phase: 20 vol% MeOH and 80 vol% ultrapure water with 0.1 vol% trifluoroacetic acid. Flow rate: 3 mL min^−1^. Retention time: 18.2 min.

For **2**, 3*, 4*, 9*, **11**, and 15*, a second isolation step was performed using a VP250/10 Nucleodur C_18_ Isis, 5 μm (Macherey‐Nagel) column under following conditions: Column oven: 30°. Mobile phase: ACN and ultrapure water with 0.1 vol% trifluoroacetic acid. The flow rate and gradient or isocratic conditions varied depending on the compound being isolated (see later).

For compound **2** an isocratic isolation step with 20 vol% ACN for 15 min was used. Flow rate: 3 mL min^−1^. Retention time: 8.3 min.

For compound 3*, the following chromatographic conditions were applied: Flow rate: 3 mL min^−1^. Gradient: 0–5 min 20 vol% ACN; 5–20 min: 20–50 vol% ACN; 20–21 min: 50 vol% ACN; 21–22 min: 50–20 vol% ACN; 22–25 min: 20 vol% ACN. Retention time: 14.6 min.

For compound 4* an isocratic isolation step with 40 vol% ACN for 13 min was used. Flow rate: 3 mL min^−1^. Retention time: 7.3 min.

Gradient for the purification of 9* and **11**: 0–5 min: 20 vol% ACN; 5–35 min: 20–50 vol% ACN; 35–36 min: 50 vol% ACN; 36–37 min: 50–20 vol% ACN; 37–40 min: 20 vol% ACN. Retention time: 9*—29.7 min; **11**—28.2 min.

Gradient for the purification of 15*: 0–10 min: 40 vol% ACN; 10–24 min: 40–70 vol% ACN; 24–25 min: 70 vol% ACN; 25–26 min: 70–40 vol% ACN; 26–30 min: 40 vol% ACN. Retention time: 18.2 min.

To obtain sufficient purity of 3*, a third reversed‐phase HPLC purification step was required. For that, a VP250/10 Nucleodur C_18_ Pyramid, 5 μm (Macherey‐Nagel) column was used. The following chromatographic conditions were applied: Column oven: 30 °C. Mobile phase: ACN and ultrapure water with 0.1 vol% trifluoroacetic acid. Flow: 3 mL min^−1^. Gradient: 0–5 min: 15 vol% ACN; 5–32 min: 15–32 vol% ACN; 32–34 min: 32–70 vol% ACN; 34–35 min: 70 vol% ACN; 35–36 min: 70–15 vol% ACN; 36–40 min: 15% ACN. Retention time: 28.57 min.

##### Spectroscopic Analyses

An Agilent 1260 Infinity HPLC system coupled with a Bruker Daltonics Compact quadrupole time‐of‐flight mass spectrometer was used for LC–MS analysis. For HPLC, a Nucleoshell RP 18 ec column (100 × 2 mm, 2.7 μm; Macherey–Nagel) was applied at the following conditions: Flow rate: 0.3 mL min^−1^. Column oven: 40 °C. Mobile phases: MeOH and water with 0.1 vol% formic acid. Gradient: 0–3 min: 2 vol% MeOH; 3–12 min: 2–95 vol% MeOH; 12–14 min: 95 vol% MeOH; 14–16 min: 95–2 vol% MeOH; 16–20 min: 2 vol% MeOH. MS was conducted with a capillary voltage of 4.5 kV, dry gas (N_2_) temperature of 240 °C, and a dry gas (N_2_) flow rate of 8 L min^−1^ in positive mode. LC–MS/MS analyses were performed with collision energies of 23 or 30 eV. NMR measurements were carried out at ambient temperature using a Bruker Avance III HD 600 MHz (CryoProbe) spectrometer equipped with a 5 mm helium‐cooled inverse quadrupole resonance cryoprobe. The NMR spectra were recorded using deuterated dimethylsulfoxide (DMSO) as the solvent and internal standard (DMSO: δ_H_ 2.50 ppm and δ_C_ 39.51 ppm).

3‐(3‐Amino‐4‐hydroxybenzamido)‐4‐hydroxybenzoic acid (**1**): ^1^H NMR (600 MHz, DMSO, 300 K): *δ *= 7.50 (dd, *J *= 8.3, 1.6 Hz, 1H, CH‐1), 6.92 (d, *J *= 8.3 Hz, 1H, CH‐2), 7.56 (d, *J *= 1.6 Hz, 1H, CH‐5), 9.25 (s, 1H, NH‐11), 8.40 (d, *J *= 2.1 Hz, 1H, CH‐13), 7.63 (dd, *J *= 8.5, 2.1 Hz, 1H, CH‐15), 6.98 (d, *J *= 8.5 Hz, 1H, CH‐16); ^13^C NMR (150 MHz, DMSO, 300 K): *δ *= 122.5* (C‐1), 114.6 (C‐2), 150.6* (C‐3), 125.3 (C‐4), 118.7* (C‐5), 164.7 (C‐7), 126.0 (C‐12), 124.6 (C‐13), 121.5 (C‐14), 127.0 (C‐15), 115.2 (C‐16), 152.9 (C‐17), 167.1 (C‐18); HRMS (ESI): *m/z* calcd for C_14_H_12_N_2_O_4_: 289.0819 [M+H]^+^; found: 289.0809.

2‐(3‐Amino‐4‐hydroxyphenyl)benzo[*d*]oxazole‐5‐carboxylic acid (**2**): ^1^H NMR (600 MHz, DMSO, 300 K): *δ *= 7.98 (dd, *J *= 8.5, 1.7 Hz, 1H, CH‐1), 7.82 (d, *J *= 8.0 Hz, 1H, CH‐2), 8.23 (d, *J *= 1.7 Hz, 1H, CH‐5), 7.58 (dd, *J *= 8.3, 2.0 Hz, 1H, CH‐14), 6.95 (d, *J *= 8.3 Hz, 1H, CH‐15), 7.70 (d, *J *= 2.1 Hz, 1H, CH‐18); ^13^C NMR (150 MHz, DMSO, 300 K): *δ *= 126.4 (C‐1), 110.6 (C‐2), 152.9 (C‐3), 142.0 (C‐4), 120.4 (C‐5), 127.6 (C‐6), 167.0 (C‐7), 164.3 (C‐11), 150.1* (C‐12), 120.7* (C‐14), 115.2 (C‐15), 150.1* (C‐16), 117.0 (C‐17), 116.1* (C‐18); HRMS (ESI): *m/z* calcd for C_14_H_10_N_2_O_4_: 271.0713 [M+H]^+^; found: 271.0714.

Closoxazole C (3*): ^1^H NMR (600 MHz, DMSO, 300 K): *δ *= 7.90 (dd, *J *= 8.5, 2.1 Hz, 1H, CH‐1), 7.13 (d, *J *= 8.5 Hz, 1H, CH‐2), 8.81 (d, *J *= 2.1 Hz, 1H, CH‐5), 9.23 (s, 1H, NH‐8), 11.07 (s, 1H, OH‐9), 8.26 (d, *J *= 1.5 Hz, 1H, CH‐13), 8.01 (dd, *J *= 8.5, 1.5 Hz, 1H, CH‐15), 7.86 (d, *J *= 8.5 Hz, 1H, CH‐16), 7.39 (br s, 1H, CH‐18), 6.88 (d, *J *= 8.2 Hz, 1H, CH‐19), 7.47 (br s, 1H, CH‐22); ^13^C NMR (150 MHz, DMSO, 300 K): *δ *= 124.8 (C‐1), 116.1 (C‐2), 152.2 (C‐3), 127.2 (C‐4), 121.7 (C‐5), 116.7 (C‐6), 164.1 (C‐7), 142.0 (C‐12), 120.5 (C‐13), 127.7 (C‐14), 126.5 (C‐15), 110.7 (C‐16), 153.0 (C‐17), 114.3 (C‐19), 125.3 (C‐23), 165.1 (C‐24), 167.0 (C‐28); HRMS (ESI): *m/z* calcd for C_21_H_15_N_3_O_6_: 406.1034 [M+H]^+^; found: 406.1014.

Closoxazole D (4*): ^1^H NMR (600 MHz, DMSO, 300 K): *δ *= 7.88 (dd, *J *= 8.4, 2.1 Hz, 1H, CH‐1), 7.12 (d, *J *= 8.4 Hz, 1H, CH‐2), 8.78 (d, *J *= 2.1 Hz, 1H, CH‐5), 9.22 (s, 1H, NH‐8), 8.26 (br s, 1H, CH‐13), 8.00 (dt, *J *= 8.5 Hz, 1.6 Hz, 1H, CH‐15), 7.86 (d, *J *= 8.5 Hz, 1H, CH‐16), 7.74 (d, *J *= 8.5 Hz, 1H, 2 × CH‐20), 6.66 (d, *J *= 8.5 Hz, 1H, 2 × CH‐21); ^13^C NMR (150 MHz, DMSO, 300 K): *δ *= 124.5 (C‐1), 116.4 (C‐2), 152.2 (C‐3), 127.5 (C‐4), 121.8 (C‐5), 116.7 (C‐6), 164.1 (C‐7), 141.9 (C‐12), 120.5 (C‐13), 127.6 (C‐14), 126.5 (C‐15), 110.7 (C‐16), 153.0 (C‐17), 167.0 (C‐18), 129.3 (C‐20), 112.9 (C‐21), 152.3 (C‐22), 120.1 (C‐23), 165.3 (C‐24); HRMS (ESI): *m/z* calcd for C_21_H_15_N_3_O_5_: 390.1084 [M+H]^+^; found: 390.1070.

Closoxazole E (9*): ^1^H NMR (600 MHz, DMSO, 300 K): *δ *= 7.93 (dd, *J *= 8.5, 2.2 Hz, 1H, CH‐1), 7.14 (d, *J *= 8.5 Hz, 1H, CH‐2), 8.75 (d, *J *= 2.2 Hz, 1H, CH‐5), 9.43 (s, 1H, NH‐8), 10.98 (s, 1H, OH‐9), 8.26 (d, *J *= 1.8 Hz, 1H, CH‐13), 8.00 (dd, *J *= 8.5 Hz, 1.8 Hz, 1H, CH‐15), 7.86 (d, *J *= 8.5 Hz, 1H, CH‐16), 7.37 (dd, *J *= 2.6, 1.5 Hz, 1H, CH‐20), 7.00 (ddd, *J *= 8.0, 2.5, 1.0 Hz, 1H, CH‐22), 7.35 (t, *J *= 8.0, 7.7 Hz, 1H, CH‐25), 7.43 (dt, *J *= 7.7, 1.4 Hz, 1H, CH‐26), 9.81 (s, 1H, OH‐27); ^13^C NMR (150 MHz, DMSO, 300 K): *δ *= 125.3 (C‐1), 116.2 (C‐2), 152.8 (C‐3), 126.5 (C‐4), 122.5 (C‐5), 116.6 (C‐6), 164.0 (C‐7), 141.9 (C‐12), 120.5 (C‐13), 127.7 (C‐14), 126.7 (C‐15), 110.7 (C‐16), 153.0 (C‐17), 167.0 (C‐18), 114.3 (C‐20), 157.5 (C‐21), 118.9 (C‐22), 135.6 (C‐23), 165.2 (C‐24); 129.7 (C‐25), 117.9 (C‐26); HRMS (ESI): *m/z* calcd for C_21_H_14_N_2_O_6_: 391.0925 [M+H]^+^; found: 391.0916.

2‐(3‐Hydroxyphenyl)benzo[*d*]oxazole‐5‐carboxylic acid (**11**): ^1^H NMR (600 MHz, DMSO, 300 K): *δ *= 8.04 (dd, *J *= 8.5, 1.7 Hz, 1H, CH‐1), 7.89 (d, *J *= 8.5 Hz, 1H, CH‐2), 8.31 (d, *J *= 1.7 Hz, 1H, CH‐5), 13.12 (s, 1H, OH‐9), 7.66 (ddd, *J *= 7.6, 1.6, 1.0 Hz, 1H, CH‐14), 7.43 (t, *J *= 8.1, 7.9 Hz, 1H, CH‐15), 7.05 (ddd, *J *= 8.1, 2.6, 1.0, 1H, CH‐16), 7.61 (dd, *J *= 2.6, 1.6, 1H, CH‐18), 10.00 (s, 1H, OH‐19); ^13^C NMR (150 MHz, DMSO, 300 K): *δ *= 127.0 (C‐1), 111.0 (C‐2), 153.0 (C‐3), 141.6 (C‐4), 121.0 (C‐5), 128.0* (C‐6), 167.0* (C‐7), 163.7 (C‐11), 127.1 (C‐12), 118.3 (C‐14), 130.6 (C‐15), 119.6 (C‐16), 157.9 (C‐17), 113.8 (C‐18); HRMS (ESI): *m/z* calcd for C_14_H_9_NO_4_: 256.0604 [M+H]^+^; found: 256.0609.

Closoxazole F (15*): ^1^H NMR (600 MHz, DMSO, 300 K): *δ *= 7.94 (dd, *J *= 8.5, 2.2 Hz, 1H, CH‐1), 7.15 (d, *J *= 8.5 Hz, 1H, CH‐2), 8.72 (d, *J *= 2.2 Hz, 1H, CH‐5), 9.60 (s, 1H, NH‐8), 10.95 (s, 1H, OH‐9), 8.26 (d, *J *= 1.8 Hz, 1H, CH‐13), 8.00 (dd, *J *= 8.5, 1.8 Hz, 1H, CH‐15), 7.86 (d, *J *= 8.5, 1H, CH‐16), 8.01 (dd, *J *= 8.5, 1.6 Hz, 1H, 2 × CH‐20), 7.56 (t, *J *= 8.5, 7.4 Hz, 1H, 2 × CH‐21), 7.63 (dt, *J *= 7.4, 1.6 Hz, 1H, CH‐22); ^13^C NMR (150 MHz, DMSO, 300 K): *δ *= 125.5 (C‐1), 116.3 (C‐2), 153.1 (C‐3), 126.6 (C‐4), 123.1 (C‐5), 116.6 (C‐6), 164.0 (C‐7), 141.9 (C‐12), 120.5 (C‐13), 127.7 (C‐14), 126.5 (C‐15), 110.7 (C‐16), 153.0 (C‐17), 167.0 (C‐18), 127.6 (C‐20), 131.9 (C‐22), 134.2 (C‐23), 165.4 (C‐24); HRMS (ESI): *m/z* calcd for C_21_H_14_N_2_O_5_: 375.0975 [M+H]^+^; found: 375.0954.

*Chemical shifts were deduced from HSQC or HMBC spectra.

## Conflict of Interest

The authors declare no conflict of interest.

## Supporting information

Supplementary Material^[^ ^15^ ^]^

## Data Availability

The data that support the findings of this study are available in the supplementary material of this article.

## References

[1] A. Abdullahi , K. Y. Yeong , Med. Chem. Res. 2024, 33, 406.

[2] a) D.‐L. Dong , Y. Luan , T.‐M. Feng , C.‐L. Fan , P. Yue , Z.‐J. Sun , R.‐M. Gu , B.‐F. Yang , Eur. J. Pharmacol. 2006, 545, 161; 16859676 10.1016/j.ejphar.2006.06.063

[3] a) K. H. Michel , L. D. Boeck , M. M. Hoehn , N. D. Jones , M. O. Chaney , J. Antibiot. 1984, 37, 441; 10.7164/antibiotics.37.441 6547431

[4] H. Song , C. Rao , Z. Deng , Y. Yu , J. H. Naismith , Angew Chem. Int. Ed. 2020, 59, 6054. 10.1002/anie.201915685 PMC7204872 31903677

[5] M. Lv , J. Zhao , Z. Deng , Y. Yu , Chem. Biol. 2015, 22, 1313. 26496684 10.1016/j.chembiol.2015.09.005

[6] A. A. Losada , C. Cano‐Prieto , R. García‐Salcedo , A. F. Braña , C. Méndez , J. A. Salas , C. Olano , Microb. Biotechnol. 2017, 10, 873. 28417606 10.1111/1751-7915.12716 PMC5481532

[7] L. Winand , D. J. Vollmann , J. Hentschel , M. Nett , Catalysts 2021, 11, 892.

[8] L. Winand , S. Theisen , S. Lütz , K. Rosenthal , M. Nett , Catalysts 2023, 13, 229.

[9] T. Horch , E. M. Molloy , F. Bredy , V. G. Haensch , K. Scherlach , K. L. Dunbar , J. Franke , C. Hertweck , Angew Chem. Int. Ed. 2022, 61, e202205409. 10.1002/anie.202205409 PMC9400959 35656913

[10] C. Cano‐Prieto , A. A. Losada , A. F. Braña , C. Méndez , J. A. Salas , C. Olano , ChemBioChem 2015, 16, 1925. 26083234 10.1002/cbic.201500261

[11] B. Roux , C. T. Walsh , Biochemistry 1992, 31, 6904. 1637823 10.1021/bi00145a006

[12] a) L. Winand , L. Lernoud , S. A. Meyners , K. Kuhr , W. Hiller , M. Nett , ChemBioChem 2023, 24, e202200635; 36484355 10.1002/cbic.202200635

[13] D. G. Gibson , L. Young , R.‐Y. Chuang , J. C. Venter , C. A. Hutchison , H. O. Smith , Nat. Methods 2009, 6, 343. 19363495 10.1038/nmeth.1318

[14] J. Quan , J. Tian , Nat. Protoc. 2011, 6, 242. 21293463 10.1038/nprot.2010.181

[15] F. W. Studier , B. A. Moffatt , J. Mol. Biol. 1986, 189, 113. 3537305 10.1016/0022-2836(86)90385-2

