# Supplementary material for: Discovery and In Vitro Reconstitution of Closoxazole Biosynthesis from Pyxidicoccus fallax
Source: Chembiochem. 2025 Apr 28;26(10):e202500126. doi: 10.1002/cbic.202500126 (PMC12118335; doi:10.1002/cbic.202500126)
Supplement: Supplementary file 1 — Supplementary Material[ 15 ] [file CBIC-26-e202500126-s001.pdf]

ChemBioChem

Discovery and in vitro Reconstitution of Closoxazole Biosynthesis from *Pyxidicoccus fallax*

Supporting Information

Table of contents

Gene sequences 1

Table S 1. Strains and plasmids used in this study. 2

Table S 2. Primers used in this study. 3

Figure S 1. LC-MS chromatogram of raw extract of *P. fallax* DSM 14698 supplemented with 3,4-AHBA. 3

Figure S 2. LC-MS/MS fragmentation pattern of the ligation product (**1**), the benzoxazole (**2**), and closoxazole A (**3**) 4

Figure S 3. Schematic map of plasmid pET28a-*pfxA* 5

Figure S 4. Schematic map of plasmid pET28a-*pfxB* 5

Figure S 5. Schematic map of plasmid pET28a-*pfxC*. 6

Figure S 6. SDS PAGE analysis of PfxA. 6

Figure S 7. SDS PAGE analysis of PfxB. 7

Figure S 8. SDS PAGE analysis of PfxC.. 7

Figure S 9. LC-MS/MS fragmentation pattern of closoxazole A (**3**). 8

Figure S 10. ^{1}H NMR spectrum (600 MHz, DMSO-d_6) of closoxazole A (**3**). 8

Figure S 11. ^{1}H-decoupled ^{13}C NMR spectrum (150 MHz, DMSO-d_6) of **3**. 8

Figure S 12. Raw extract of *E. coli* BL21(DE3): pET28a-*pfxC* supplemented with 3,4-AHBA. 9

Table S 3. NMR data of **1** in DMSO-d_6. 9

Figure S 13. ^{1}H NMR spectrum (600 MHz, DMSO-d_6) of **1**. 10

Figure S 14. ^{1}H-decoupled ^{13}C NMR spectrum (150 MHz, DMSO-d_6) of **1**. 10

Figure S 15. COSY spectrum (DMSO-d_6) of **1**. 11

Figure S 16. HSQC spectrum (DMSO-d_6) of **1**. 11

Figure S 17. HMBC spectrum (DMSO-d_6) of **1**. 12

Figure S 18. LC-MS chromatograms of the ligation product formation using the AMP ligase PfxC and the aryl carboxylic acids 3,4-AHBA, 3-HBA, 4-HBA, 3-ABA, or 4-ABA as substrates. 12

Figure S 19. Schematic map of plasmid pET28a-*pfxBC* 13

Figure S 20. Raw extract of *E. coli* BL21(DE3): pET28a-*pfxBC* supplemented with 3,4-AHBA. 13

Table S 4. NMR data of **2** in DMSO-d_6. 14

Figure S 21. ^{1}H NMR spectrum (600 MHz, DMSO-d_6) of **2**. 15

Figure S 22. ^{1}H-decoupled ^{13}C NMR spectrum (150 MHz, DMSO-d_6) of **2**. 15

Figure S 23. COSY spectrum (DMSO-d_6) of **2**. 16

Figure S 24. HSQC spectrum (DMSO-d_6) of **2**. 16

Figure S 25. HMBC spectrum (DMSO-d_6) of **2**. 17

Figure S 26. In vitro reactions with using the benzoxazole (**2**) as substrate. 17

Figure S 27. LC-MS/MS fragmentation pattern of compound **3*** 18

Table S 5. NMR data of closoxazole C (**3***) in DMSO-d_6. 19

Figure S 28. ^{1}H NMR spectrum (600 MHz, DMSO-d_6) of closoxazole C (**3***). 20

Figure S 29. ^{1}H-decoupled ^{13}C NMR spectrum (150 MHz, DMSO-d_6) of closoxazole C (**3***). 20

Figure S 30. COSY spectrum (DMSO-d_6) of closoxazole C (**3***). 21

Figure S 31. HSQC spectrum (DMSO-d_6) of closoxazole C (**3***). 21

Figure S 32. HMBC spectrum (DMSO-d_6) of closoxazole C (**3***).. 22
Figure S 33. Raw extract of *E. coli* BL21(DE3): pET28a-*pfxBC* supplemented with 3,4-AHBA and 4-ABA. ... 23
Figure S 34. LC-MS/MS fragmentation pattern of closoxazole D (**4***). 23
Table S 6. NMR data of closoxazole D (**4***) in DMSO-d_6. 24
Figure S 35. ^{1}H NMR spectrum (600 MHz, DMSO-d_6) of closoxazole D (**4***). 25
Figure S 36. ^{1}H-decoupled ^{13}C NMR spectrum (150 MHz, DMSO-d_6) of closoxazole D (**4***). 25
Figure S 37. COSY spectrum (DMSO-d_6) of closoxazole D (**4***).. 26
Figure S 38. HSQC spectrum (DMSO-d_6) of closoxazole D (**4***).. 26
Figure S 39. HMBC spectrum (DMSO-d_6) of closoxazole D (**4***).. 27
Figure S 40. LC-MS chromatograms of in vitro reactions using PfxA, PfxB and PfxC and the substrates 3,4-AHBA, 3,4-AHBA and 4-ABA, 3,4-AHBA and 3-HBA, or 3,4-AHBA and BA. 27
Figure S 41. LC-MS/MS fragmentation of closoxazole D (**4***) und closoxazole B (**4**) 28
Figure S 42. LC-MS/MS fragmentation patterns of **6** and **8** 28
Figure S 43. LC-MS/MS fragmentation patterns of **9*** and **9**. 29
Figure S 44. Raw extract of *E. coli* BL21(DE3): pET28a-*pfxBC* supplemented with 3,4-AHBA and 3-HBA. ... 30
Table S 7. NMR data of closoxazole E (**9***) in DMSO-d_6. 31
Figure S 45. ^{1}H NMR spectrum (600 MHz, DMSO-d_6) of closoxazole E (**9***). 32
Figure S 46. ^{1}H-decoupled ^{13}C NMR spectrum (150 MHz, DMSO-d_6) of closoxazole E (**9***). 32
Figure S 47. COSY spectrum (DMSO-d_6) of closoxazole E (**9***). 33
Figure S 48. HSQC spectrum (DMSO-d_6) of closoxazole E (**9***). 33
Figure S 49. HMBC spectrum (DMSO-d_6) of closoxazole E (**9***). 34
Table S 8. NMR data of **11** in DMSO-d_6. 35
Figure S 50. ^{1}H NMR spectrum (600 MHz, DMSO-d_6) of **11**. 36
Figure S 51. ^{1}H-decoupled ^{13}C NMR spectrum (150 MHz, DMSO-d_6) of **11**. 36
Figure S 52. COSY spectrum (DMSO-d_6) of **11**. 37
Figure S 53. HSQC spectrum (DMSO-d_6) of **11**. 37
Figure S 54. HMBC spectrum (DMSO-d_6) of **11**. 38
Figure S 55. LC-MS/MS fragmentation patterns of **10**, **12**, **13**, and **14**. 38
Figure S 56. Raw extract of *E. coli* BL21(DE3): pET28a-*pfxBC* supplemented with 3,4-AHBA and BA........... 39
Figure S 57. LC-MS/MS fragmentation pattern of **15***. 39
Table S 9. NMR data of closoxazole F (**15***) in DMSO-d_6.. 40
Figure S 58. ^{1}H NMR spectrum (600 MHz, DMSO-d_6) of closoxazole F (**15***)... 41
Figure S 59. ^{1}H-decoupled ^{13}C NMR spectrum (150 MHz, DMSO-d_6) of closoxazole F (**15***).......................... 41
Figure S 60. COSY spectrum (DMSO-d_6) of closoxazole F (**15***). 42
Figure S 61. HSQC spectrum (DMSO-d_6) of closoxazole F (**15***). 42
Figure S 62. HMBC spectrum (DMSO-d_6) of closoxazole F (**15***). 43
Figure S 63. LC-MS/MS fragmentation patterns of **16** and **18**. 43

Gene sequences

Gene sequence of HG543_RS13880 (*pfxA*) from *P. fallax* DSM 14698

ATGCGCTTCAGCTTTGAAAAGAGATTACGGGCCCTCCAGTCCCCTCCCGTCATTTCGCGCTACTTCTC
CGGGGAGGGGCATGCCGACACCATGCCTCCCGCGCGGCTCGCCCGGTACCAGCTCGAAGCCCTCAAGG
CCATCGTCCAGCGGGCATATGACCAGTCGCCCTTCTACAGGGAGAAGATGACCGGGGCCGGAGTCTCT
CCCGGCGACCTGGAGCGGCTGGAGGACCTCTCCAAGCTGCCCTTCCTGACGAAGGACGAGCTGCGCGG
GCGCCCCTGGCTCCTGCTGACCTGCGACAAGAAGGACGTCGTGCTCATCCAGGTGTCCACCGGGACGA
CCGGCGGTGAAGAAATCTACATGACGTATACGTGGAACGACTATCTGCTCCACGACCTGTCCCCGCGC
TACGGGCACCTCTTCCCGGTCGGCCCCGGCGACGTGTGCCTCAACGCCCTGCCGTACGAGATGAGCAC
GGCGGGGCTCTCCTTTCACAAGACCTTCATGGATGGCTACCAGTCCACGGTCATCCCCGCCGGAAAGG
GCGGCGCCTACTCCACGCCCGCGAAGACCCTGAAGATGATCCGCGACCTGCGGCCCAACGTCGTGGTC
ACCAGTCCCTCCTGGTCCATGACCCTGGCGGAGGAAGCGGCCAGCGGCTCCTTCGACCTGAAGAGCCT
GGGCATCAAGAAGATGTGGCTGACCGGCGAAGGCTGCTCGCCTGCCTTCCGCCGGCGCGTGGAGAACA
TCTGGGGCACCACGGCCAACTACTTCTACGGCTCGCTCGAGTGCGGAGCGCTCGGCATCGAGTGCGAT
GCACACAACGGTTACCACCTGACCCAGGCCCACGTGCTCATGGAGATTGTCGACCCGAAGACCGGGGT
GAGCCTGCCTCCTGGGGAGATTGGCGAAATCGTCGTGACGGCGTTACTGCGCTACGACAGCCCGGTCA
TCCGGTTCCGCACGGGGGACCTGGGCTCCCTGGACACCGCCGCATGCGCCTGCGGCTCCACGCTCACC
CGGTTCCACATGAAGGGCCGCGCGTTCGACCAGCTCCACTTCCGTGGCCGGCCGCTCTCGCCCTTCTT
CCTGGAGGAGTTCCTGATGCGGATGCCGGAGGTCGGCAACTGGTTCCAGTTCGTCATGCCGGCCTCGG
ACAGCGCGCGCATCAAGATTCGCTGCGAGCTGGCGGACGGAGTCCCACCCTCCGCGGAGCTGGCCGCC
ACGCTCGCCAGCAGGATGGAGGCGTCGACGGGGCTGCCCTTCGACATCGAGCTCGTCGACCACCTGCC
GCGCCCGAATGGCAAGGCGGTCCGGGTCGTTCGTGAGTGA

Gene sequence of HG543_RS13885 (*pfxB*) from *P. fallax* DSM 14698, codon optimized for *E. coli*

ATGATCATTGATGCACATGCACATGTTAGCCCGACCACCTATGGTGCAACCGAAAAATATCTGGAAGT
TCTGAAACAGAGCGGTATTGATCAGGCAGTTATTTGTCCTGGTGGTATGCTGGATGTTCGTAAAATGA
GCGAATTTGTTAGCGGTCAGAAAAAGCCGGATACCGTTCCGAAAAATGATTATGTTGCACGTTGTGTT
GTTAGCAGCCCGAGCCTGTTAGGTATGGCATGTGTTGATCCGACCGATCCGCGTGCAGCAGAAAAACT
GGAAGAACTGCTGGAACAGGGTTTTCGTGGTCTGATGGTTAGTCCGCTGGTTCACAAATTTAGCTTTC
CGGATGAAGCAATGGCAGATCTGGCATGGCTGTGTGGTGAACATGATGTTCCGGTTATTAGCCATAAT
GGTTGGCGTCCGGGTGCAAATACCGTTGATTATGTGCAGCTGGCACGTCGTTTTCCGGGTACAAACTT
TATTCTGGAACATATGGGTGCACTGCCGGTTGATGTTGAAGCAGCAGATGCAGCAGCCGAACTGGATA
ACCTGTTTCTGGAAACCAGCCTGAGCAGCTATCTGCATCTGGCAGGCACCGTTAAAAAGACCGGTGCA
AGCAAAGTTCTGTTTGGTAGCGAATATCCGCTGAGCCATCCGGCACTGGAACTGCGTAAAATCTTCCT
GCTGCCGCTGACCGATGATGAACGTGAACGCATTTTAGGTGGTAATATTCGCGGTCTGCTGCGTCTGG
ATTAA

Gene Sequence of HG543_RS13890 (*pfxC*) from *P. fallax* DSM 14698

```
ATGGCAACGCAAGAACGCCTGGAGCGCATCAATCAGGTGCTGCGGCACGCACGCGGCGCGGACTTCTA
CAAAGAGCGCCTGCCGGCCACGCCGCTGCGCACCTGGGAGGAGTTCCAGCGGCTTCCCTTCACGACCA
AGGAGGACCTGCGCCGGCAGTCACCGCACGGGATGGTCTGTGTCCCGCGGCAGGAGCTGCTGCAGTAC
CACGAGTCCTCCGCGACGACCGGCGCGCCCGTCTCCGTCTGGTACAGCGGCAAGGACCTGGCGGAAAT
CCAGGCGCGCTTCTCGGAGTGGGGCGTCGGGTTCATGCCCGGGGACCGGGTGCTCATCCGCTTCCCCT
ATGCCCTCTCCACCATCGGGCACTTCGTCCACGCGGCCGCCCAGCACAAGCGGGCCTGTGTCATCCCC
GCGGACAGCCGGACCAGCATCACGCCGCTGCCGCGCGTGGTCGAGCTCATGAGGAAGTTGCAAGTCAC
CGTCCTCGCCACCATCTCCCTGTCCGCGGTGATGATCGCCGAAGCCGCGGAGATGGCGGGGTTCGAGC
CCCGTCGCGACTTTCCCCACCTGCGTGCCATCTGCTCCGCGGGAGAGCCGCTGACCCAGGCCCGGCGC
AAGCTCCTGGAGGAAATCTGGGGAGTCCCTGTGTATGACAATTATGGAATGACAGAGACGGGCCCGCA
GGCCATGGACTGCCGGGTGCAGCAGCTCCATCCCTGGCAGGGCCACTTCTGCATGGAAGTCCTGGACG
AGCGGCTCGAGAAGGAGGTGGCGCCGGGTGAGACGGGCTACCTGGTCGTCACGTCCCTCACCCCGAGG
GCCTCGCCCGTGATTCGCTATCTCACCGGAGACCGCGTCCAGCGCATGGAGCGGCCCTGCGAGTGCGG
CCAGAGCTCGACGCTGCGCGTCCGCGGCCGGGTGGAGGACGTCCTGTGGAGCCAGGGCAGGCCGTTGG
ACCTCTGGGAGCTGGAGGAGATTGTCTCCCAGCTGCCGGGCCGCCGCTTCTGGAGGGTGGCTTCCGCG
CCGGACGGGCGGCTGCACTTCGTCGTGGAGCAGGAGCGTGACGGGGACTCGCTGCGGCCTGCCCTCGT
GTCGCGGTTGGAAGCGCACCATGGCGTCCGCATGAAGGTGGACCTGGTCCCCAAGGGGACCCTCTACG
ACCGGAACGAGCCGGTCTCGTTCGGAATGGCGGGCAAGCCCATCTACGTGTGCACGCCCCAGTCCATG
CCCGAGGTCCGAGCGTAG
```

Table S 1. Strains and plasmids used in this study.

**Strain or Plasmid**	**Specification**	**Reference**
E. coli TOP10	F⁻ *mcr*A Δ(*mrr-hsd*RMS-*mcr*BC) φ80*lac*ZΔM15 Δ*lac*X74 *rec*A1 *ara*D139 Δ(*ara-leu*)7697 *gal*U *gal*K λ⁻ *rps*L(Str^{R})	Thermo Fisher Scientific
E. coli BL21(DE3)	F⁻ *omp*T *gal dcm lon hsd*SB(*rB*⁻ *mB*⁻) λ(DE3 [lacI *lac*UV5- T7p07 *ind*1 *sam*7 *nin*5]) [*mal*B+]$_{K-12}$(λ^{S})	Studier and Moffatt, 1986[15]
Pyxidicoccus fallax DSM 14698	-	DSMZ
pET28a(+)	*E. coli* vector for expression of His-tagged proteins	Novagen
pET28a-*pfxA*	pET28a(+) vector carrying HG543_RS13880 gene fused to a *N*-terminal His-tag coding sequence	This study
pET28a-*pfxB*	pET28a(+) vector carrying *E. coli* codon optimized HG543_RS13885 gene fused to a *N*-terminal His-tag coding sequence	This study
pET28a-*pfxC*	pET28a(+) vector carrying the HG543_RS13890 gene fused to a *N*-terminal His-tag coding sequence	This study
pET28a-*pfxBC*	pET28a(+) vector carrying HG543_RS13885 and HG543_RS13890 genes fused to a *N*-terminal His-tag coding sequence	This study

Table S 2. Primers used in this study.

Name	Sequence (5'→3')
P01	AGCTTGTCGACGGAGTCACTCACGAACGACCCGG
P02	CGGATCCGAATTCGAGCTCATGCGCTTCAGCTTTGAAAAG
P03	CCGCAAGCTTGTCGACGGAGCTCTTAATCCAGACGCAGCAGACCGCGAAT
P04	GTCGCGGATCCGAATTCGAGATGATCATTGATGCACATGCACATGTTAGCC
P05	CGGATCCGAATTCGAGCTCATGGCAACGCAAGAACG
P06	AGCTTGTCGACGGAGCTACGCTCGGACCTCGG
P07	GGTGCTCGAGTGCGGCCGCAAGCTTCTACGCTCGGAC
P08	CTGGACTGACTCCGTCGACATAATACGACTCACTATAGGGGAATTGTGAGCGGATAACA
P09	GATCCCGCGAAATTAATAC
P10	GGATATAGTTCCTCCTTTC

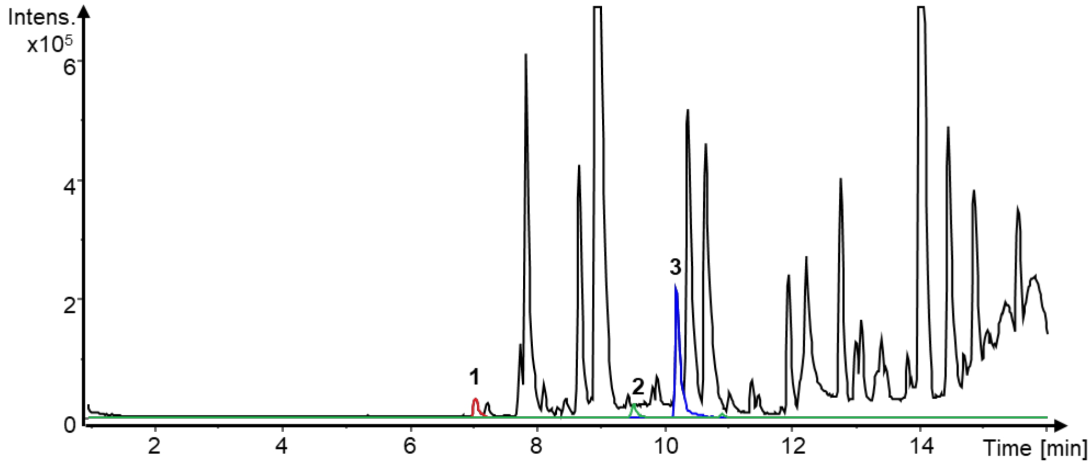

Figure S 1. LC-MS chromatogram of raw extract of *P. fallax* DSM 14698 supplemented with 50 mg L^{-1} 3,4-AHBA. Black: BPC. Red: EIC of **1** (*m/z* 289.08). Green: EIC of **2** (*m/z* 271.07). Blue: EIC of **3** (*m/z* 406.10).

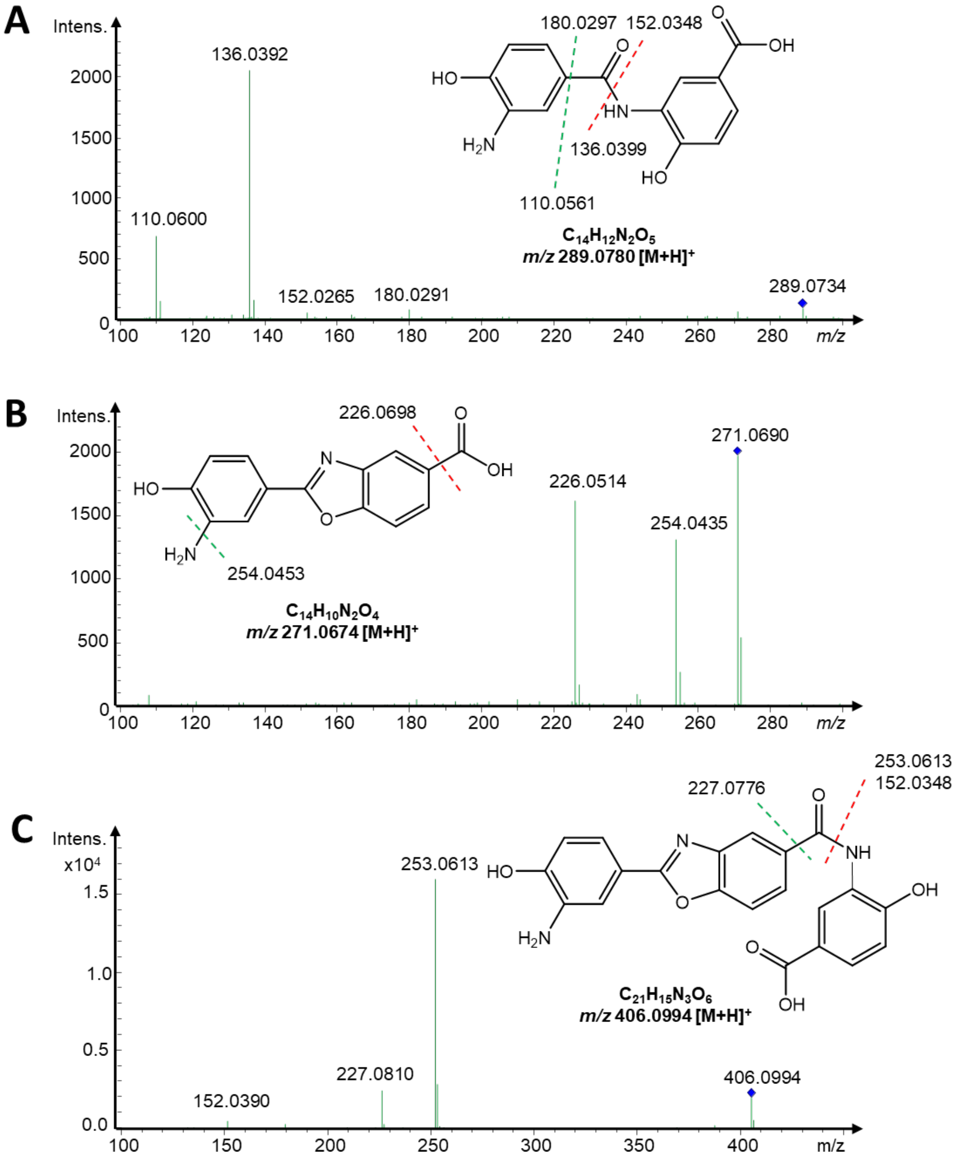

Figure S 2. LC-MS/MS fragmentation pattern of (A) the ligation product (**1**, *m/z* 289.08), (B) the benzoxazole (**2**, *m/z* 271.07), and (C) closoxazole A (**3**, *m/z* 406.10) from a raw extract of a *P. fallax* DSM 14698 culture supplemented with 50 mg L^{-1} 3,4-AHBA.

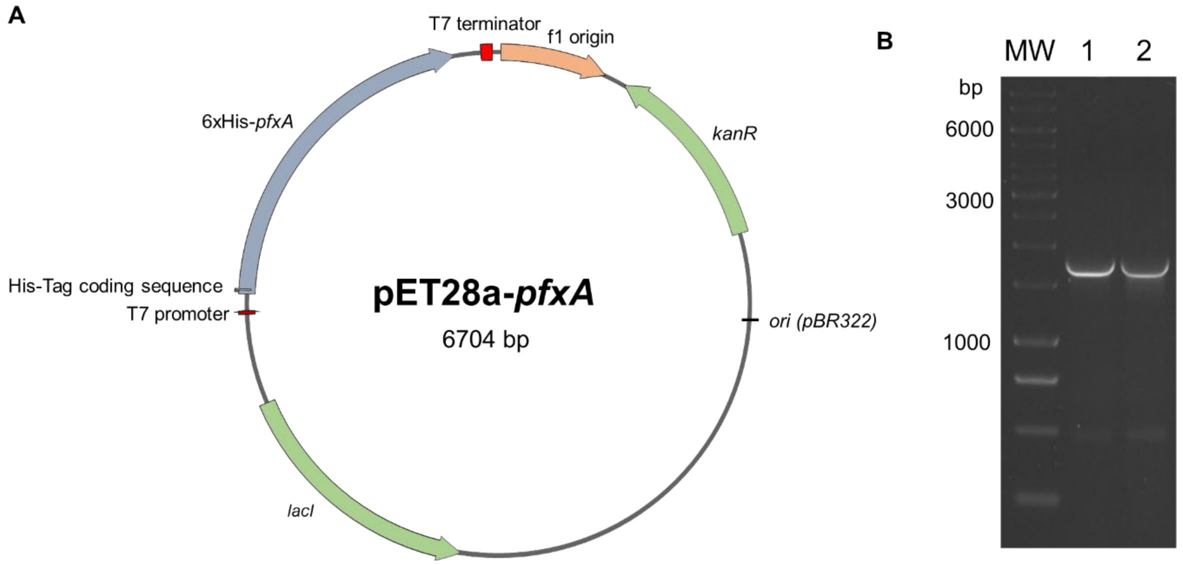

Figure S 3. (A) Schematic map of plasmid pET28a-*pfxA* harboring the *pfxA* gene from *P. fallax* DSM 14698, encoding an AMP ligase. (B) Validation of plasmid pET28a-*pfxA* via colony PCR using the primers P09/P10. 1-2: *E. coli* BL21(DE3): pET28a-*pfxA* clone 1-2. Expected PCR product: 1730 bp MW: DNA ladder.

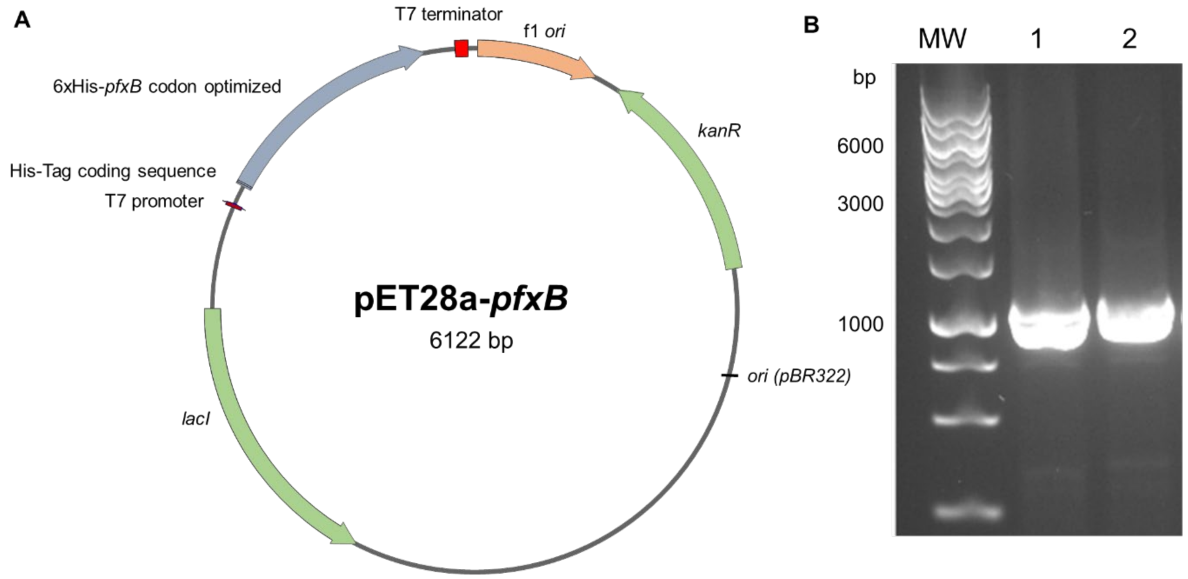

Figure S 4. (A) Schematic map of plasmid pET28a-*pfxB* carrying the codon-optimized *pfxB* originating from *P. fallax* DSM 14698, encoding a condensing amidohydrolase. (B) Validation of the plasmid via colony PCR with the primer pair P09/P10. 1-2: *E. coli* BL21(DE3): pET28a-*pfxB* clone 1-2. Expected PCR product: 1151 bp MW: DNA ladder.

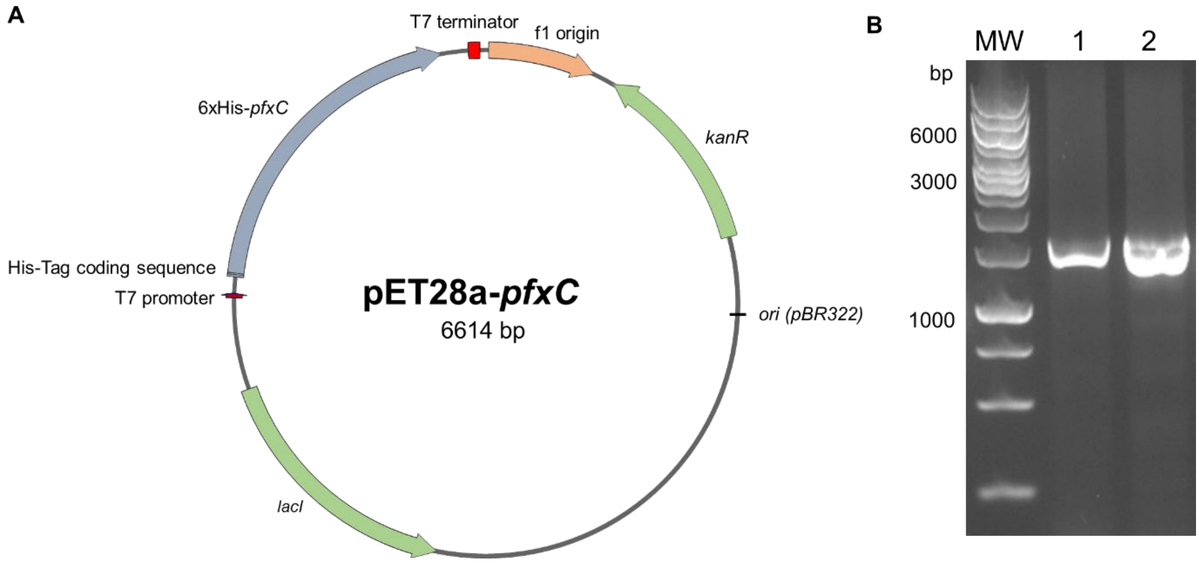

Figure S 5. (A) Schematic map of plasmid pET28a-*pfxC* harboring the *pfxC* gene from *P. fallax* DSM 14698, encoding an AMP ligase. (B) Validation of plasmid pET28a-*pfxC* via colony PCR with the primers P09/P10. 1-2: *E. coli* BL21(DE3): pET28a-*pfxC* clone 1-2. Expected PCR product: 1640 bp MW: DNA ladder.

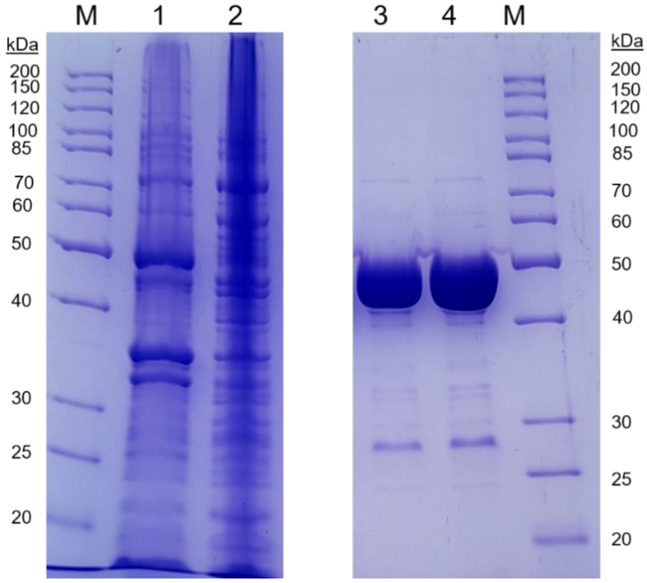

Figure S 6. SDS PAGE analysis of PfxA. 1: Cell pellet of *E. coli* BL21(DE3): pET28a-*pfxA* after cell disruption. 2: Supernatant of *E. coli* BL21(DE3): pET28a-*pfxA* after cell disruption. 3: Supernatant after Ni-NTA purification of PfxA (53.2 kDa). 4: Supernatant after desalting of purified PfxA. M: Protein Ladder.

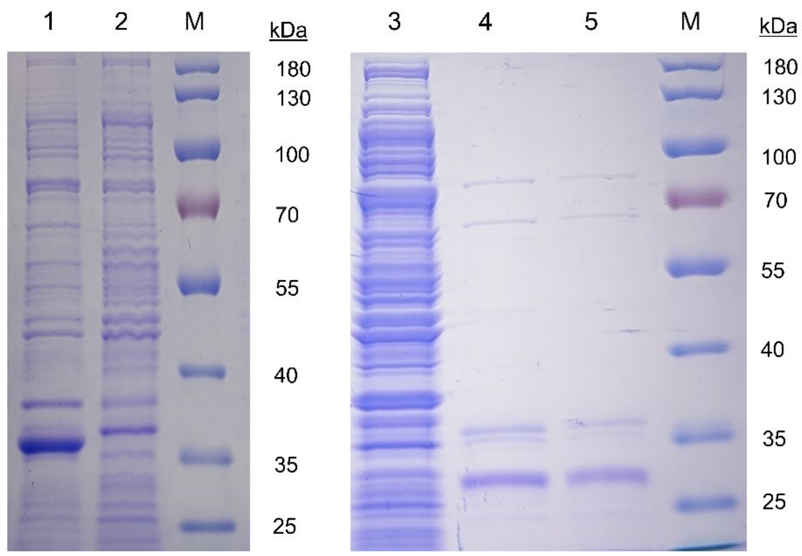

Figure S 7. SDS PAGE analysis of PfxB. 1: Cell pellet of *E. coli* BL21(DE3): pET28a-*pfxB*. 2: Cell pellet of *E. coli* BL21(DE3): pET28a(+) (negative control). 3: Supernatant of *E. coli* BL21(DE3): pET28a-*pfxB* after cell disruption. 4: Supernatant after Ni-NTA purification of PfxB (31.3 kDa). 5: Supernatant after desalting of PfxB. M: Protein ladder.

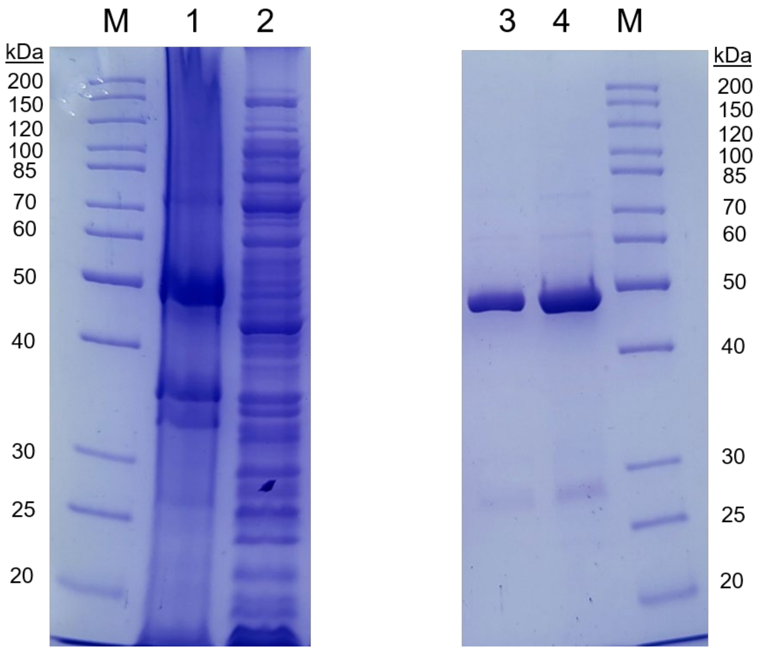

Figure S 8. SDS PAGE analysis of PfxC. 1: Cell pellet of *E. coli* BL21(DE3): pET28a-*pfxC*. 2: Supernatant of *E. coli* BL21(DE3): pET28a-*pfxC* after cell disruption. 3: Supernatant after Ni-NTA purification of PfxC (50.9 kDa). 4: Supernatant after desalting of PfxC. M: Protein ladder.

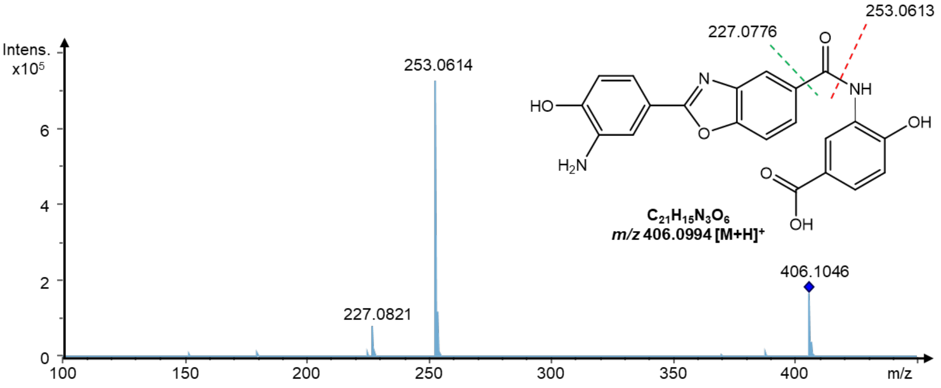

Figure S 9. LC-MS/MS fragmentation pattern of closoxazole A (**3**, *m/z* 406.10) from an in vitro reaction with the enzymes PfxA, PfxB and PfxC and 3,4-AHBA as substrate.

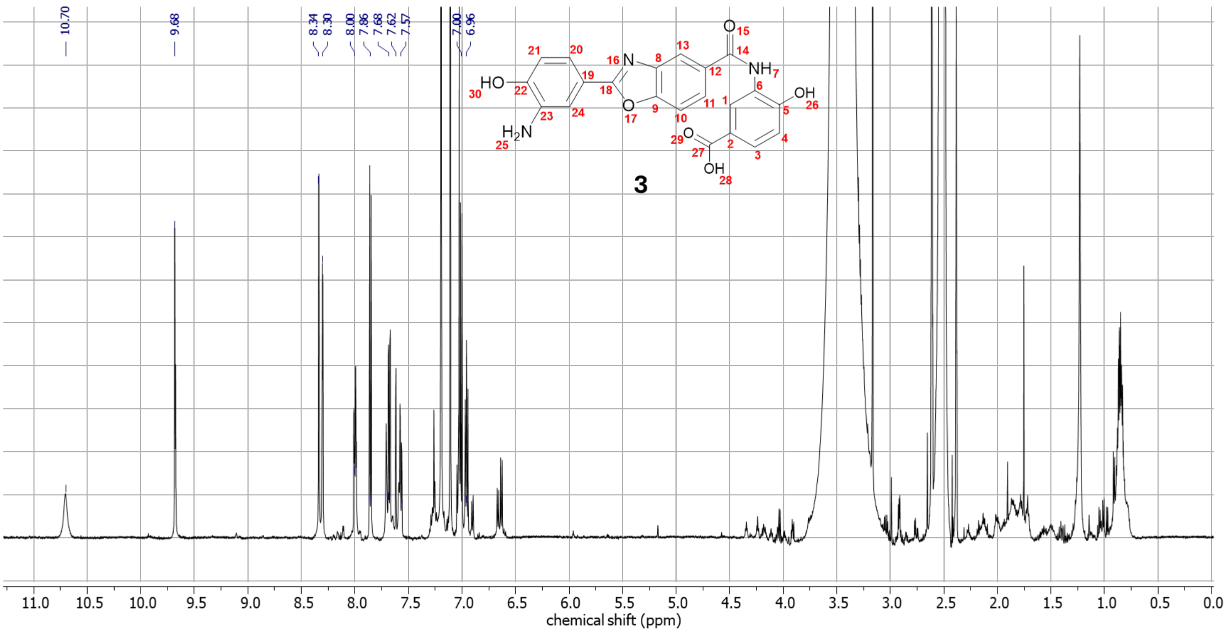

Figure S 10. ^{1}H NMR spectrum (600 MHz, DMSO-d_6) of closoxazole A (**3**). Chemical shifts are consistent with the ones previously published.[9]

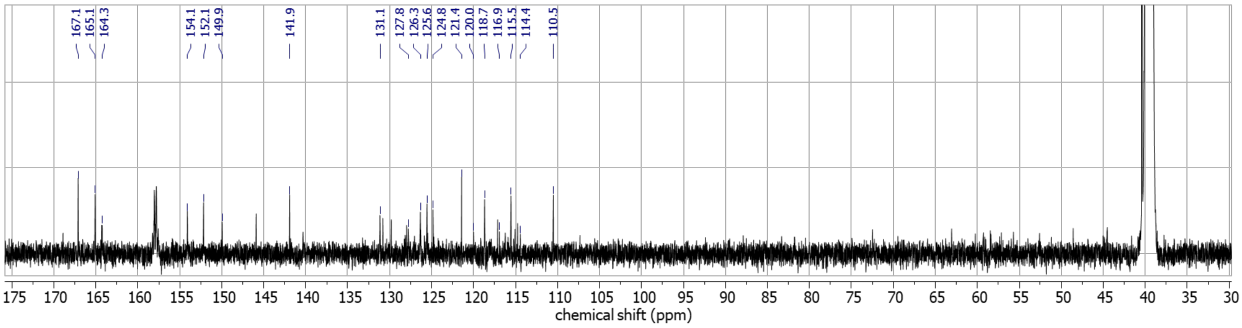

Figure S 11. ^{1}H-decoupled ^{13}C NMR spectrum (150 MHz, DMSO-d_6) of **3**. Chemical shifts are consistent with the ones previously published.[9]

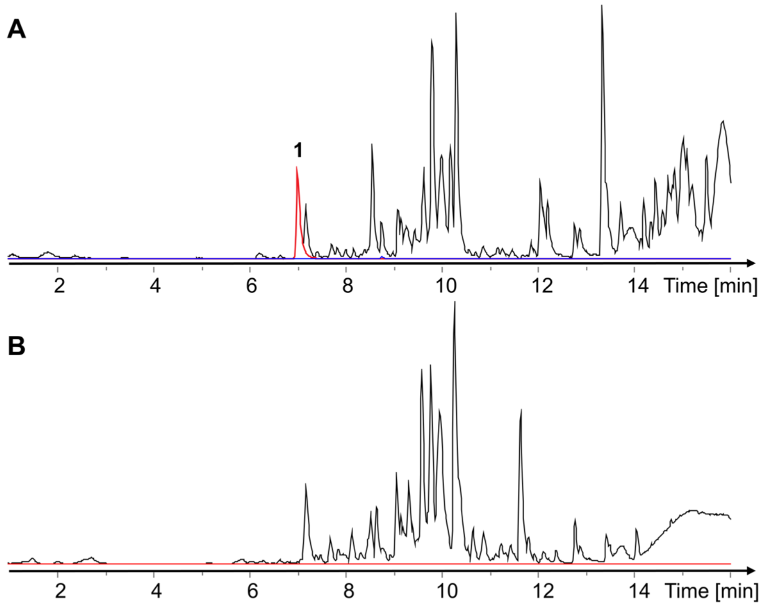

Figure S 12. (A) Raw extract of *E. coli* BL21(DE3): pET28a-*pfxC* supplemented with 50 mg L^{-1} 3,4-AHBA. (B) Raw extract of *E. coli* BL21(DE3): pET28a(+) supplemented with 50 mg L^{-1} 3,4-AHBA (negative control). Black: BPC. Red: EIC of **1** (*m/z* 289.08).

Table S 3. NMR data of **1** in DMSO-d_6. Atom numbering according to Horch et al.[9] n.d.: not detected.

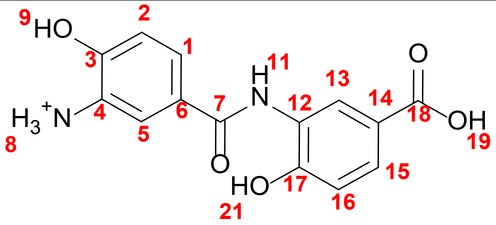

#	δ_C	type	δ_H, M (J in Hz)	COSY	HMBC ($^1H \rightarrow ^{13}C$)
1	122.5*	CH	7.50, dd (8.3, 1.6)	2	3, (7)
2	114.6	CH	6.92, d (8.3)	1	(3), 4
3	150.6*	C_q			
4	125.3	C_q			
5	118.7*	CH	7.56, d (1.6)	1	3, 7
6	n.d.	C_q			
7	164.7	C_q			
11	-	NH	9.25, s		7, 13, 17
12	126.0	C_q			
13	124.6	CH	8.40, d (2.1)	(11), 15	15, 17, 18
14	121.5	C_q			
15	127.0	CH	7.63, dd (8.5, 2.1)	13, 16	13, 17, 18
16	115.2	CH	6.98, d (8.5)	15	12, 14, 17
17	152.9	C_q			
18	167.1	C_q			
	-	OH	10.79, s		

*Chemical shifts were deduced from HSQC or HMBC spectra

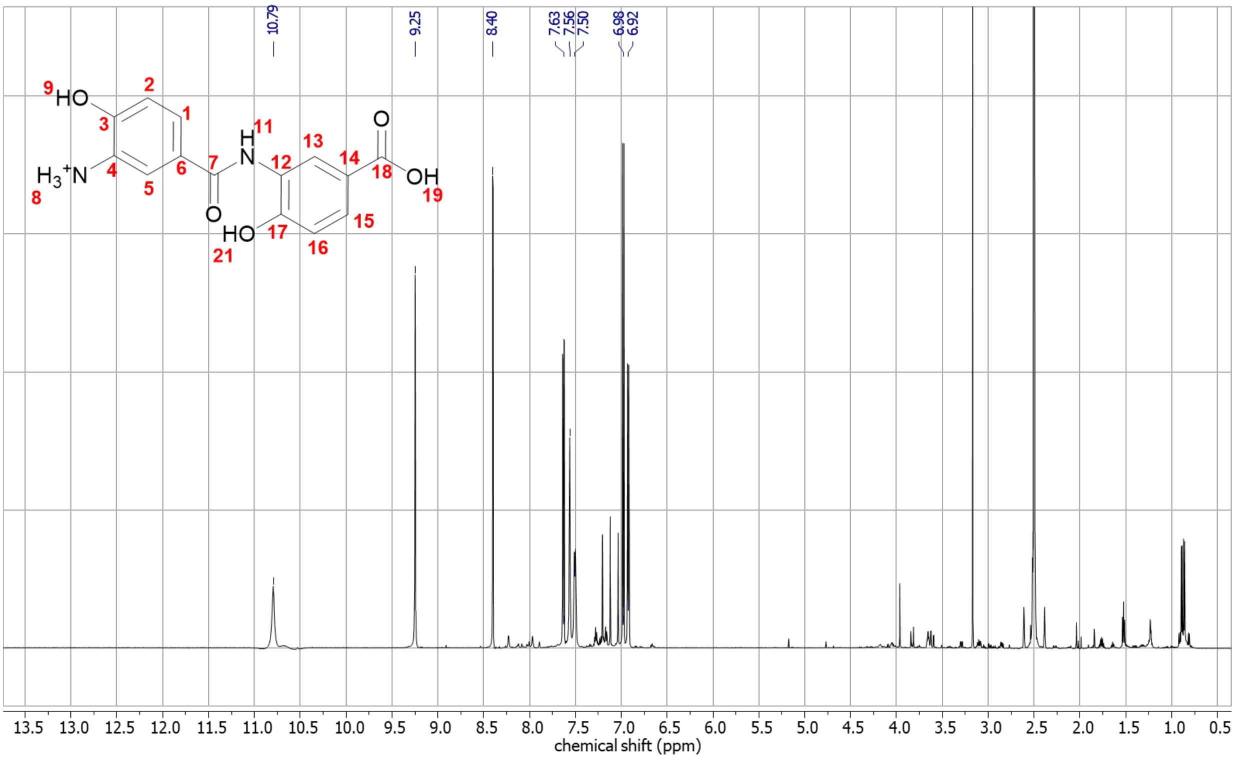

Figure S 13. ^{1}H NMR spectrum (600 MHz, DMSO-d_6) of **1**.

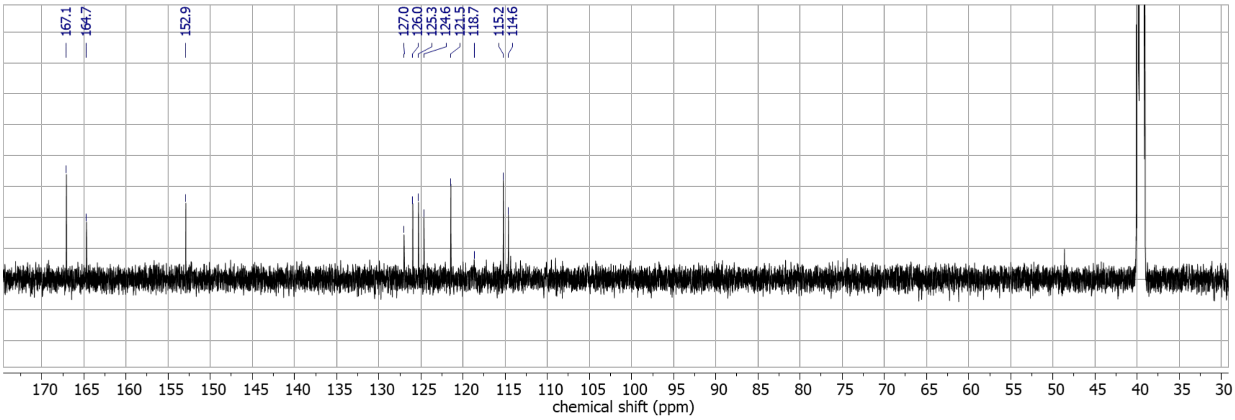

Figure S 14. ^{1}H-decoupled ^{13}C NMR spectrum (150 MHz, DMSO-d_6) of **1**.

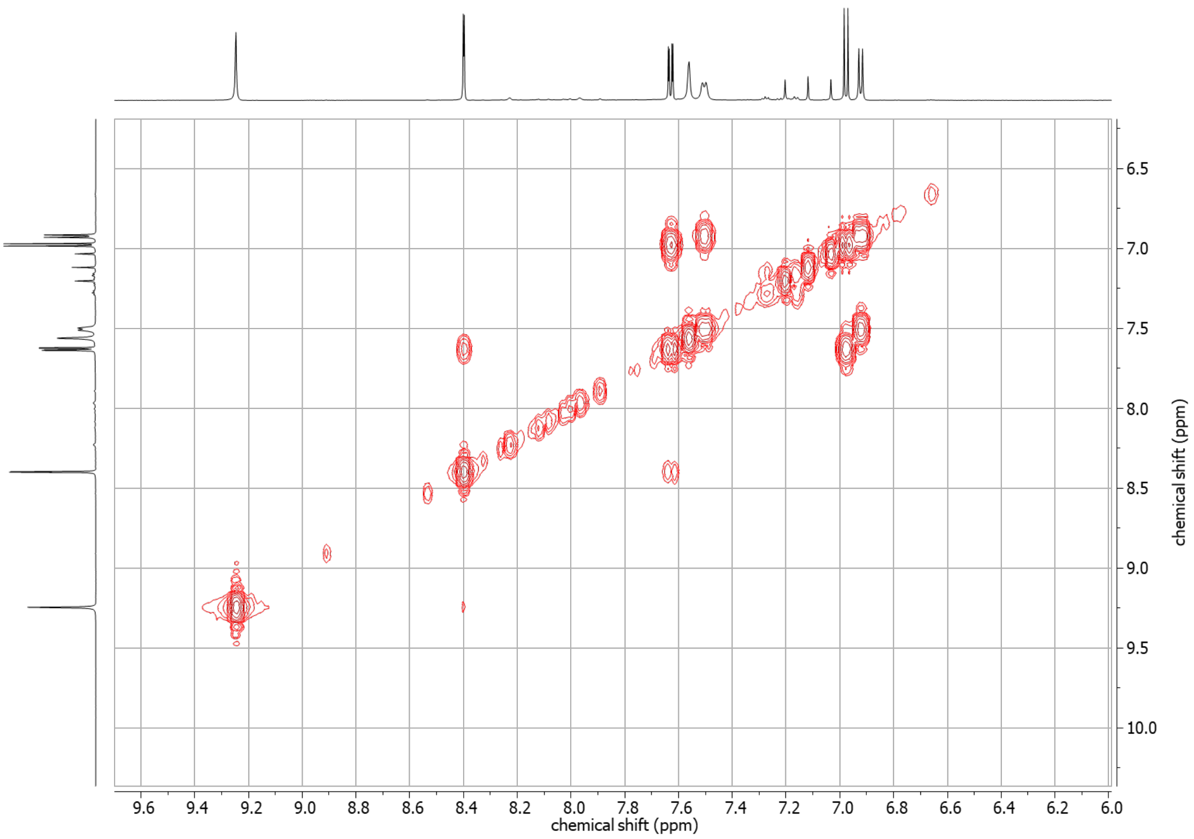

Figure S 15. COSY spectrum (DMSO-d_6) of **1**.

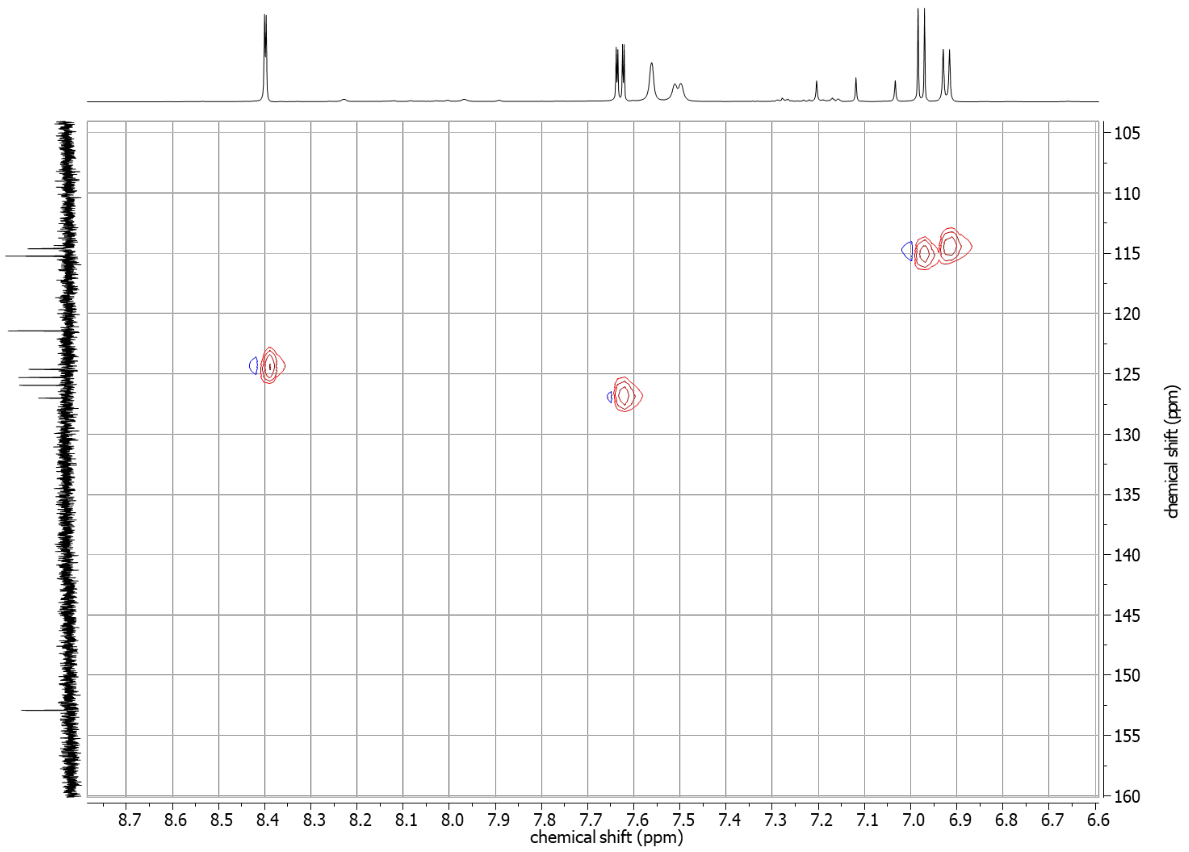

Figure S 16. HSQC spectrum (DMSO-d_6) of **1**.

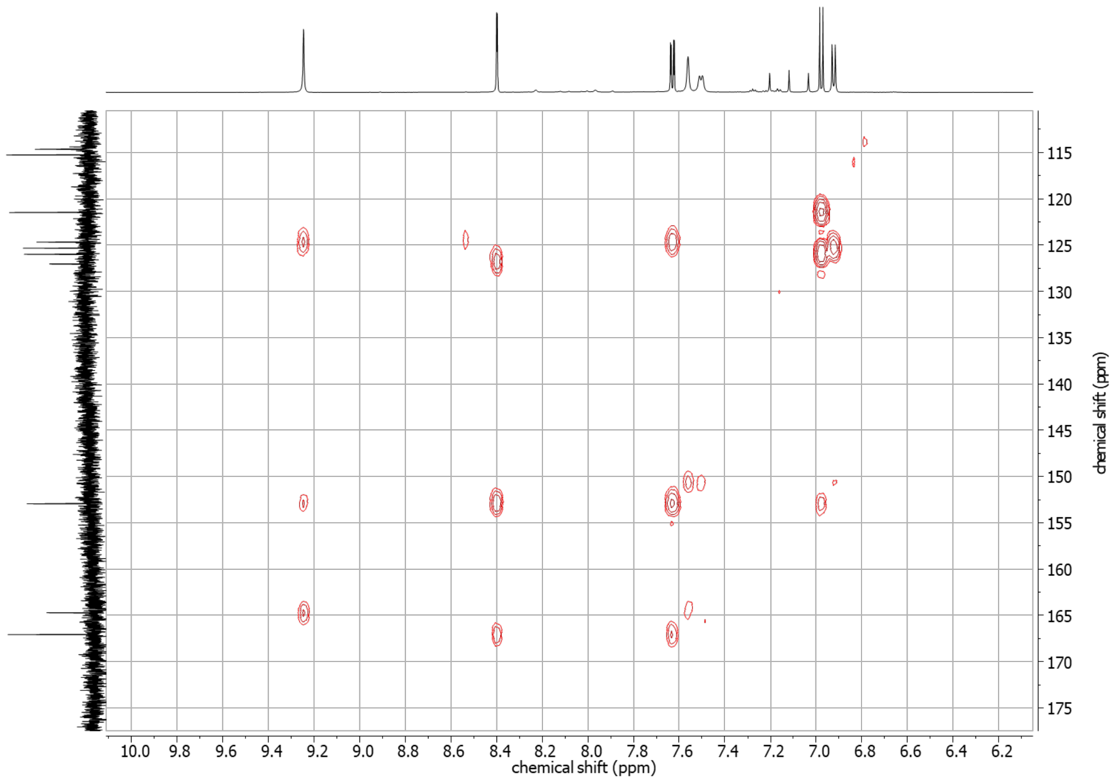

Figure S 17. HMBC spectrum (DMSO-d_6) of **1**.

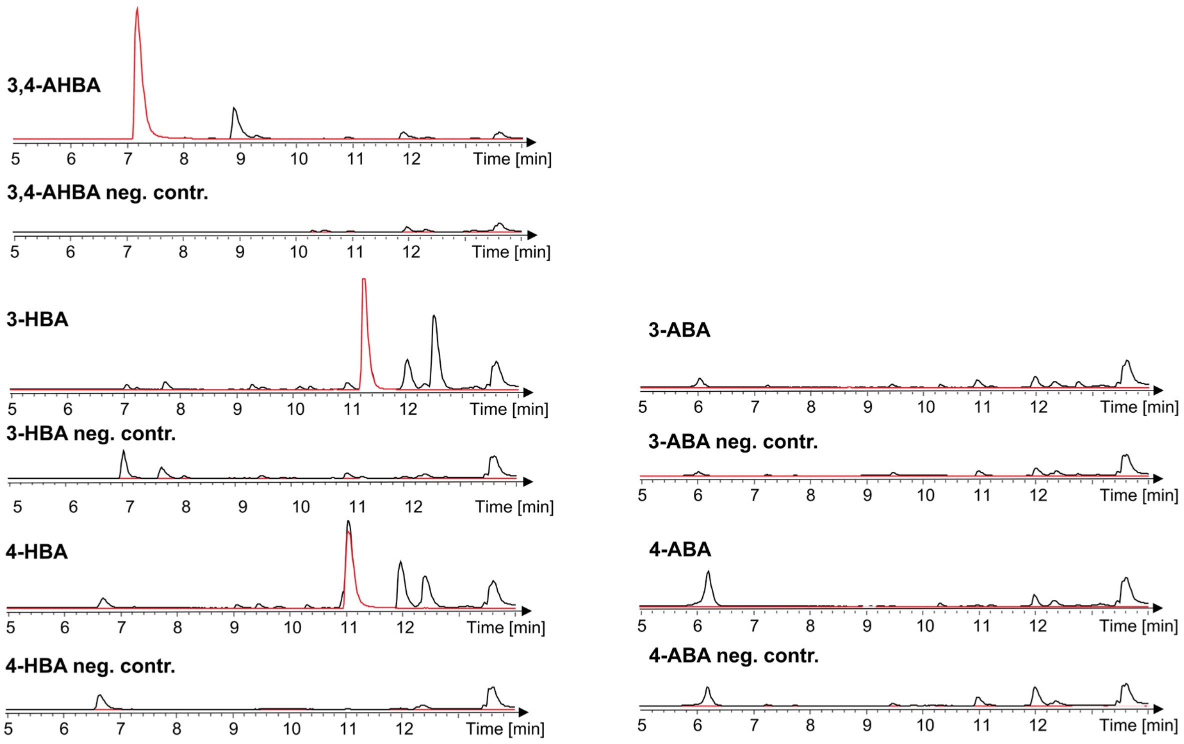

Figure S 18. LC-MS chromatograms of the ligation product formation using the AMP ligase PfxC and the aryl carboxylic acids 3,4-AHBA (*m/z* 289.08), 3-HBA (*m/z* 259.06), 4-HBA (*m/z* 259.06), 3-ABA (*m/z* 257.09), or 4-ABA (*m/z* 257.09) as substrates. Black: BPC. Red: EICs of the respective ligation products. Negative controls were performed without addition of PfxC.

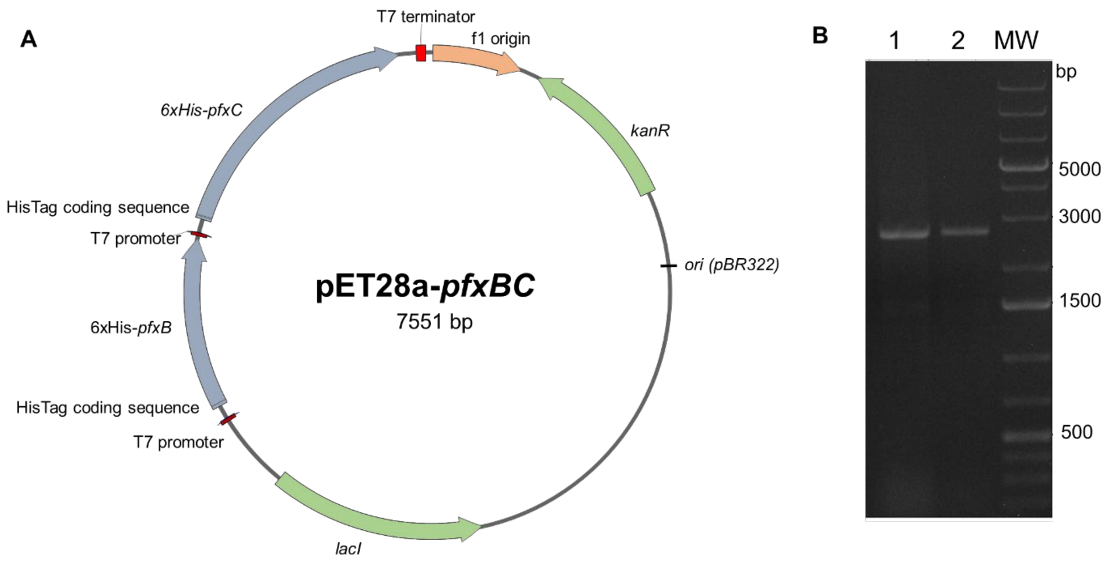

Figure S 19. (A) Schematic map of plasmid pET28a-*pfxBC* harboring the *pfxB* and *pfxC* gene from *P. fallax* DSM 14698, encoding a condensing amidohydrolase and an AMP ligase. (B) Validation of plasmid pET28a-*pfxBC* via colony PCR with the primers P09/P10. 1-2: *E. coli* BL21(DE3): pET28a-*pfxBC* clone 1-2. Expected PCR product: 2606 bp MW: DNA ladder.

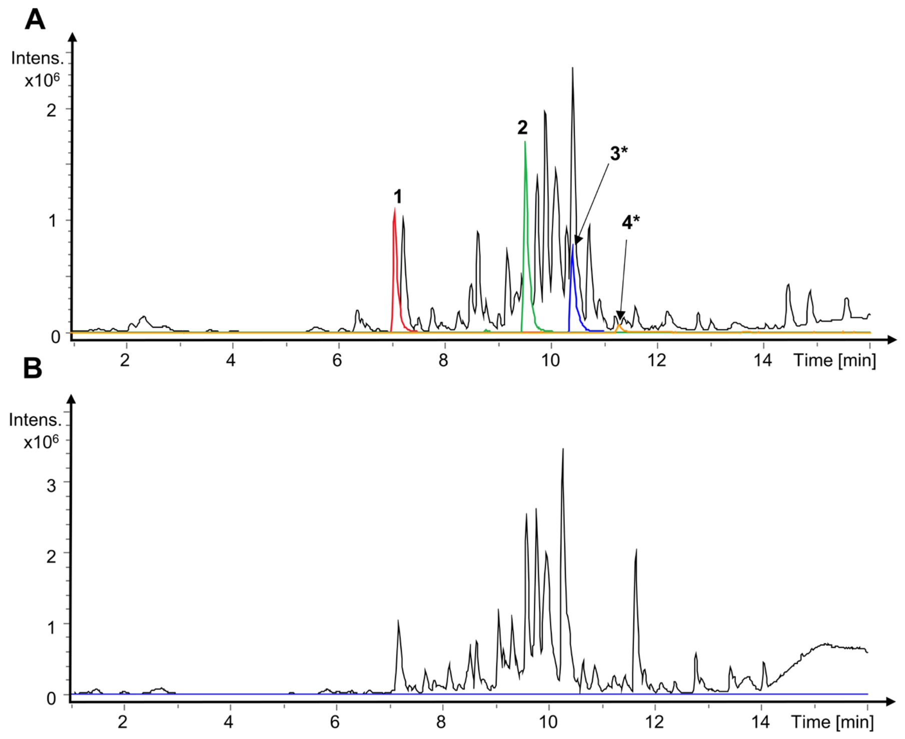

Figure S 20. (A) Raw extract of *E. coli* BL21(DE3): pET28a-*pfxBC* supplemented with 50 mg L^{-1} 3,4-AHBA. (B) Raw extract of *E. coli* BL21(DE3): pET28a(+) supplemented with 50 mg L^{-1} 3,4-AHBA (negative control). Black: BPC. Red: EIC of ligation product (**1**, *m/z* 289.08). Green: EIC of benzoxazole intermediate (**2**, *m/z* 271.07). Blue: EIC of closoxazole A (**3**, *m/z* 406.10). Yellow: EIC of **4*** (*m/z* 390.10).

Table S 4. NMR data of **2** in DMSO-d_6.

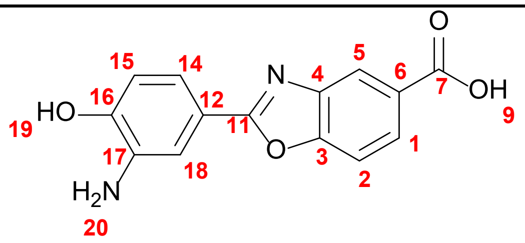

#	δ_C	type	δ_H, M (J in Hz)	COSY	HMBC ($^1H \rightarrow ^{13}C$)
1	126.4	CH	7.98, dd (8.5, 1.7)	2, 5	3, 5, 7
2	110.6	CH	7.82, d (8.5)	1	3, 4, 6
3	152.9	C_q			
4	142.0	C_q			
5	120.4	CH	8.23, d (1.7)	1	1, 3, 7
6	127.6	C_q			
7	167.0	C_q			
11	164.3	C_q			
12	150.1*	C_q			
14	120.7*	CH	7.58, dd (8.3, 2.0)	15, (18)	16, 18
15	115.2	CH	6.95, d (8.3)	14	12, 17
16	150.1*	C_q			
17	117.0	C_q			
18	116.1*	CH	7.70, d (2.1)	14	11, 14, 16
			10.57, br s		
			13.06, br s		

*Chemical shifts were deduced from HSQC or HMBC spectra

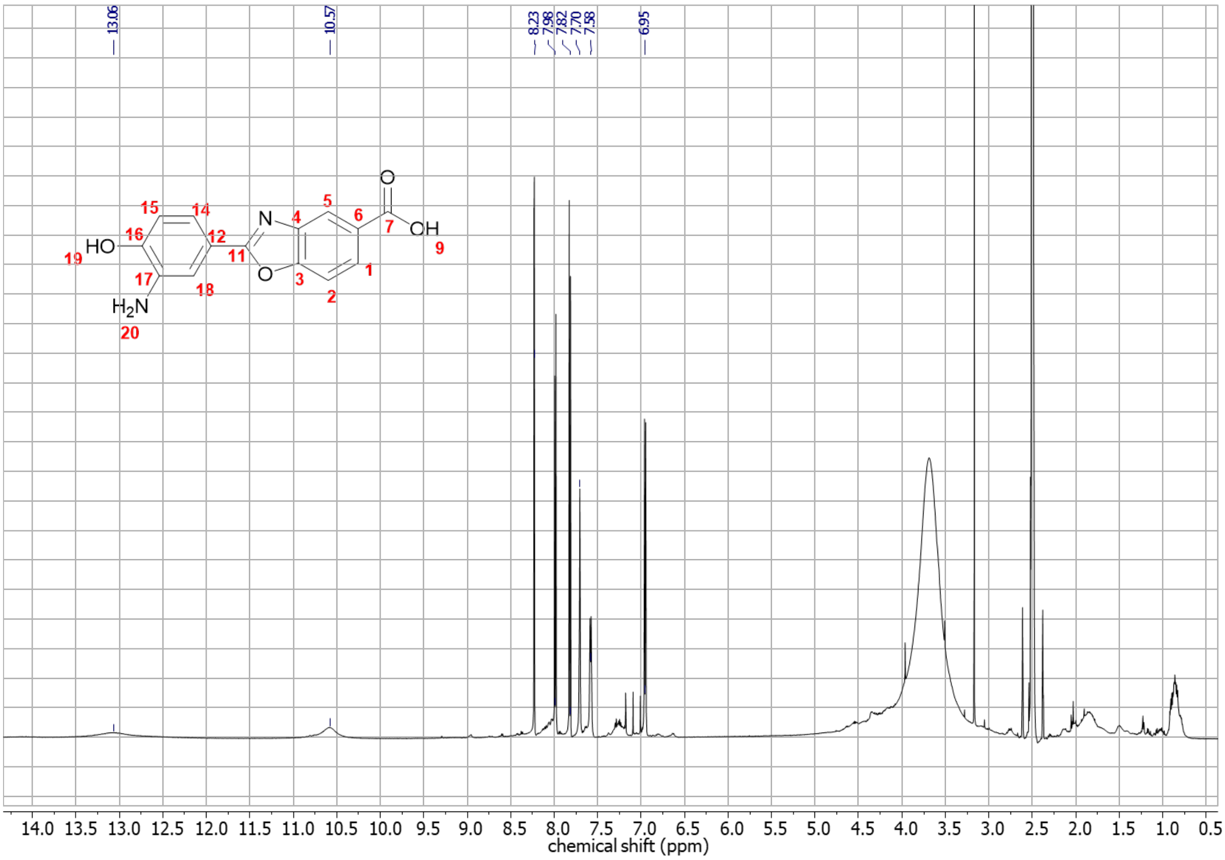

Figure S 21. ^{1}H NMR spectrum (600 MHz, DMSO-d_6) of **2**.

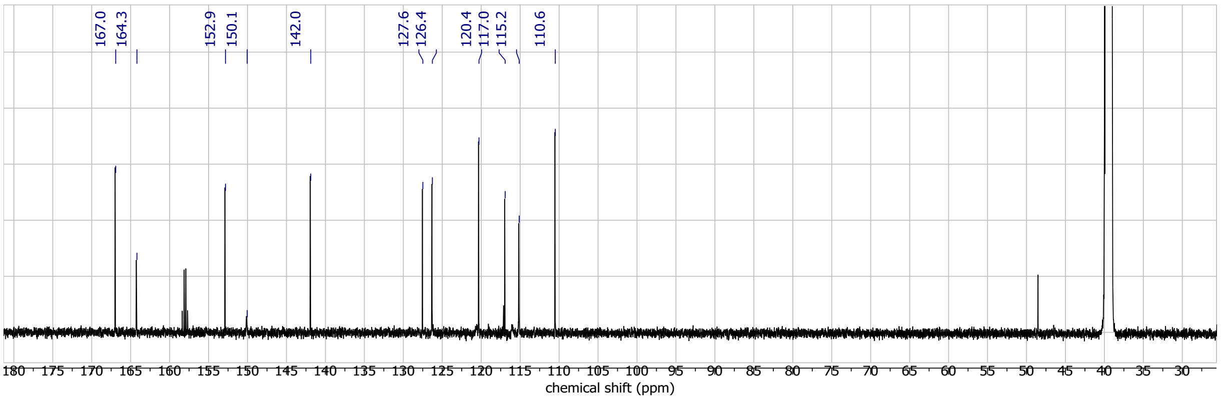

Figure S 22. ^{1}H-decoupled ^{13}C NMR spectrum (150 MHz, DMSO-d_6) of **2**.

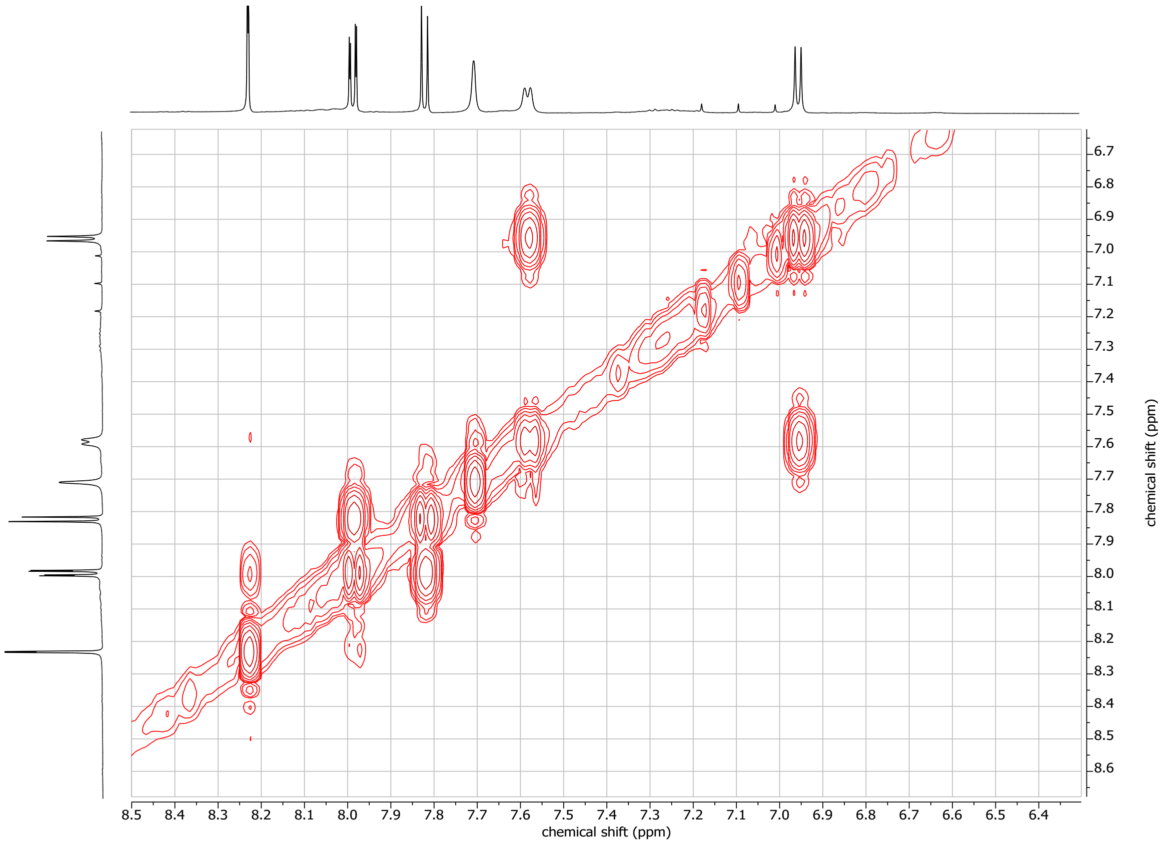

Figure S 23. COSY spectrum (DMSO-d_6) of **2**.

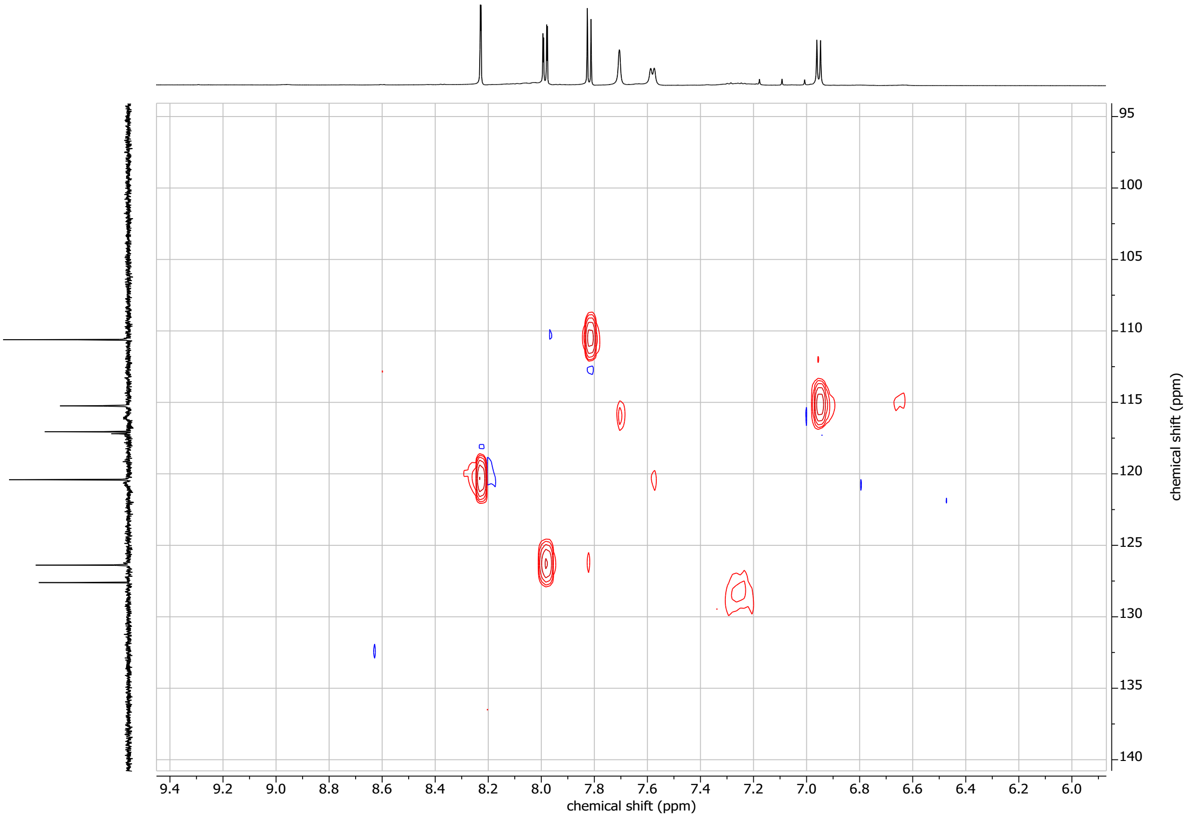

Figure S 24. HSQC spectrum (DMSO-d_6) of **2**.

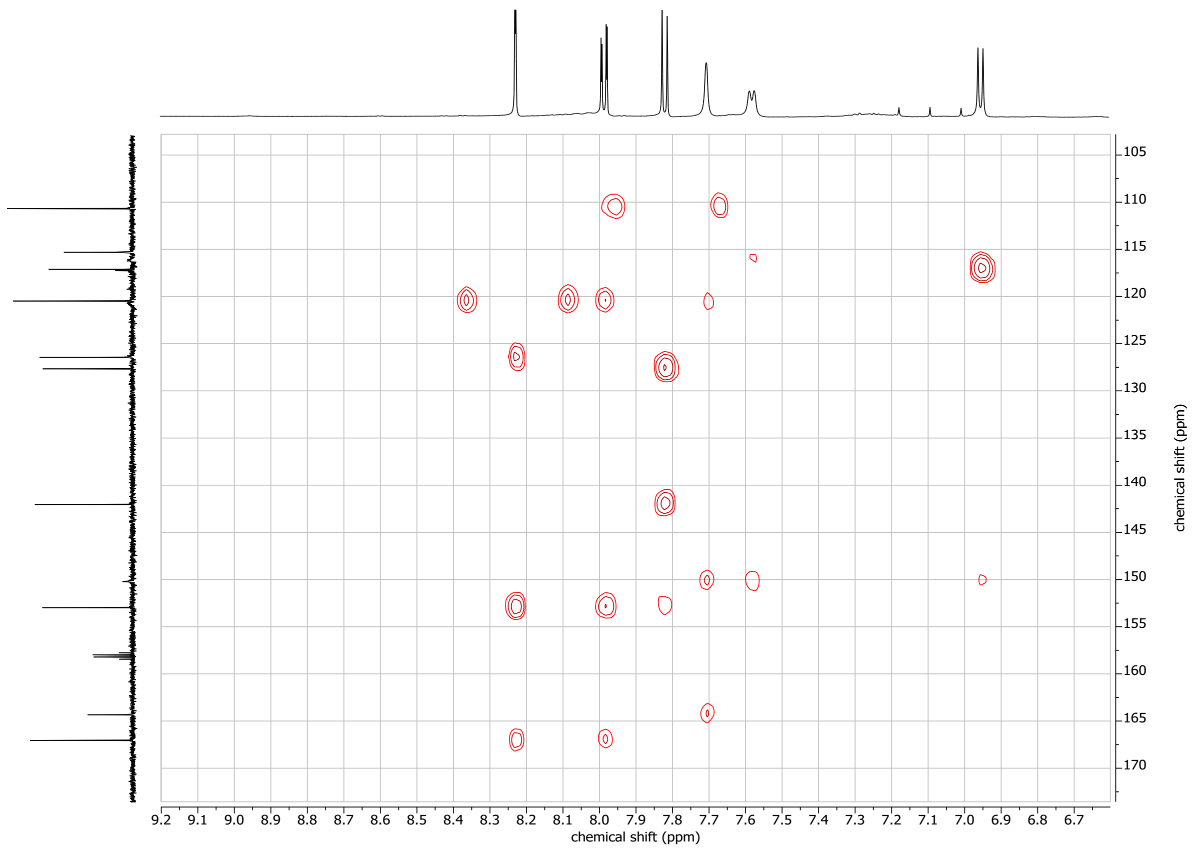

Figure S 25. HMBC spectrum (DMSO-d_6) of **2**.

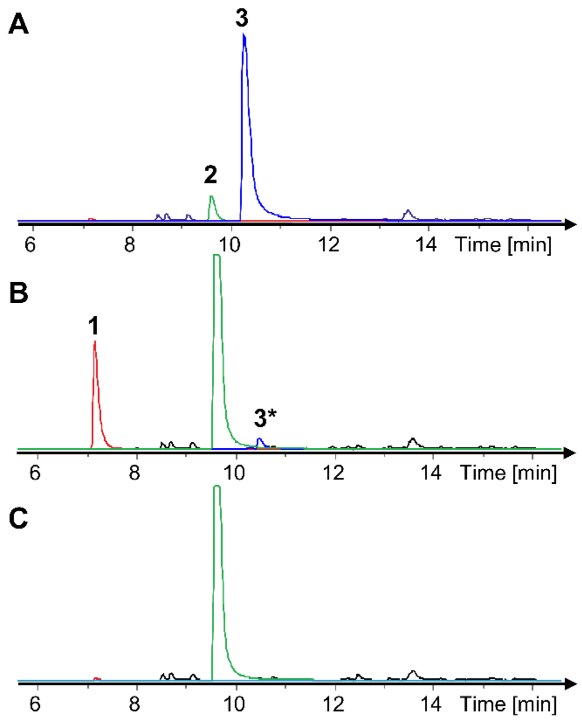

Figure S 26. In vitro reactions with using the benzoxazole (**2**) as substrate. (A) PfxA incubated with **2** and 3,4-AHBA (B) PfxC incubated with **2** and 3,4-AHBA. (C) Negative control without enzyme. Red: EIC of ligation product (**1**, *m/z* 289.08). Green: EIC of the benzoxazole (**2**, *m/z* 271.07). Blue: EIC of closoxazole A (**3**) or its constitutional isomer closoxazole C (**3***, both *m/z* 406.10).

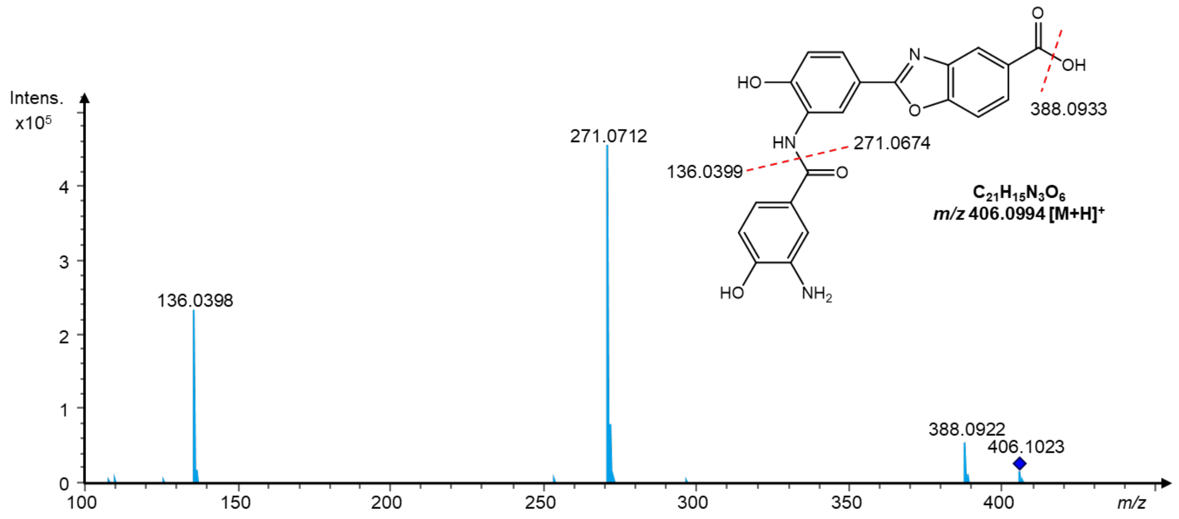

Figure S 27. LC-MS/MS fragmentation pattern of compound **3*** (*m/z* 406.10), produced in an in vitro reaction using the AMP ligase PfxC and the benzoxazole (**2**) and 3,4-AHBA as substrates.

Table S 5. NMR data of closoxazole C (**3***) in DMSO-d_6. Atom numbering according to Horch et al.[9] n.d.: not detected.

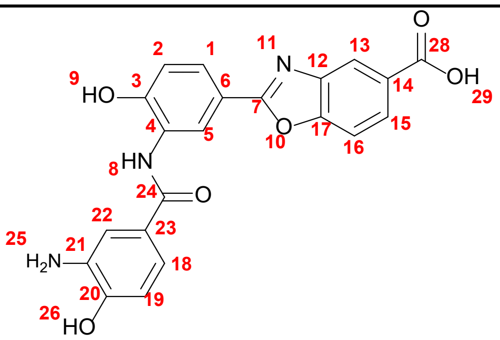

#	δ_C	type	δ_H, M (J in Hz)	COSY	HMBC ($^1H \rightarrow ^{13}C$)
1	124.8	CH	7.90, dd (8.5, 2.1)	2, 5	3, 5, 7
2	116.1	CH	7.13, d (8.5)	1	4, 6
3	152.2	C_q			
4	127.2	C_q			
5	121.7	CH	8.81, d (2.1)	1	1, 3, 7
6	116.7	C_q			
7	164.1	C_q			
8		NH	9.23, s	5	5, 24
9		OH	11.07, s		4
12	142.0	C_q			
13	120.5	CH	8.26, d (1.5)	15, (16)	15, 17, 28
14	127.7	C_q			
15	126.5	CH	8.01, dd (8.5, 1.5)	(13), 16	13, 17
16	110.7	CH	7.86, d (8.5)	15	12, 14
17	153.0	C_q			
18	n.d.	CH	7.39, br s	19	
19	114.3	CH	6.88, d (8.2)	18	23
20	n.d.	C_q			
21	n.d.	C_q			
22	n.d.	CH	7.47, br s		
23	125.3	C_q			
24	165.1	C_q			
28	167.0	C_q			
		OH	13.07, br s		
		OH	10.41, br s		

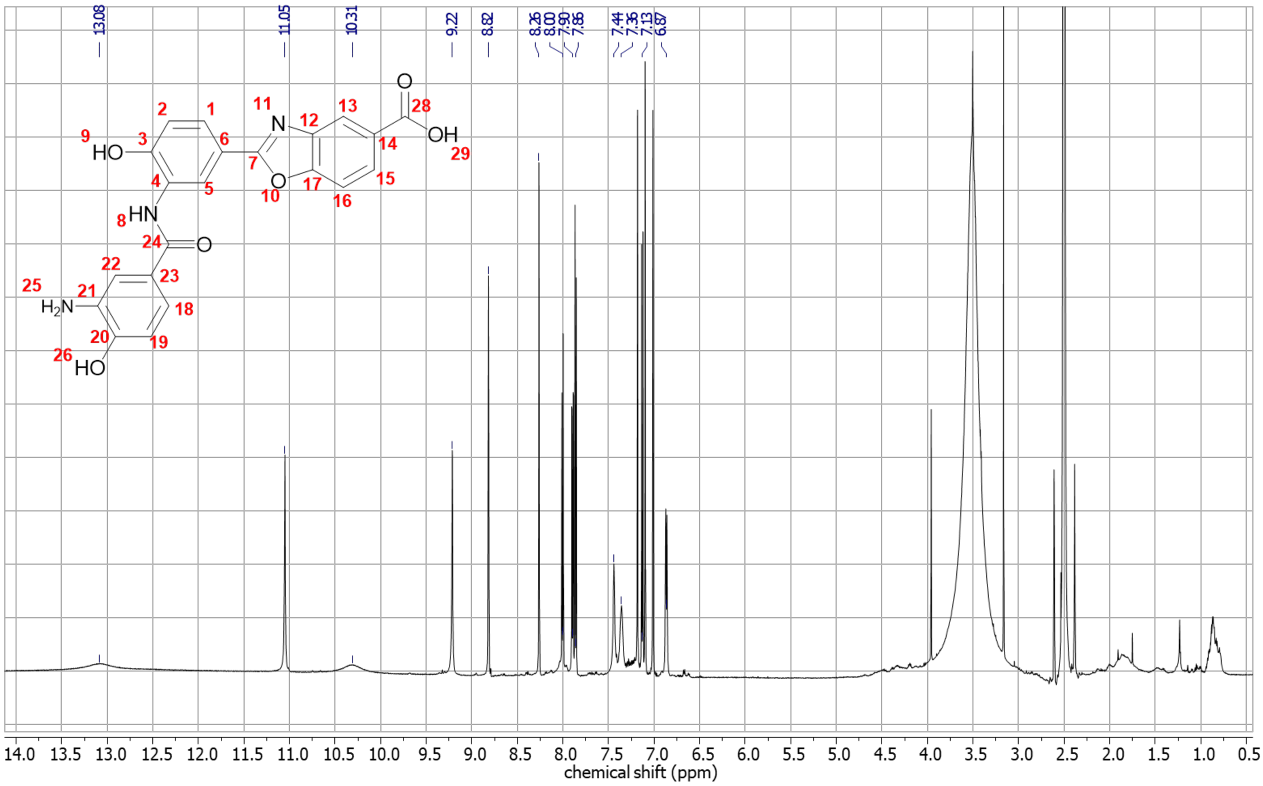

Figure S 28. ^{1}H NMR spectrum (600 MHz, DMSO-d_6) of closoxazole C (**3***).

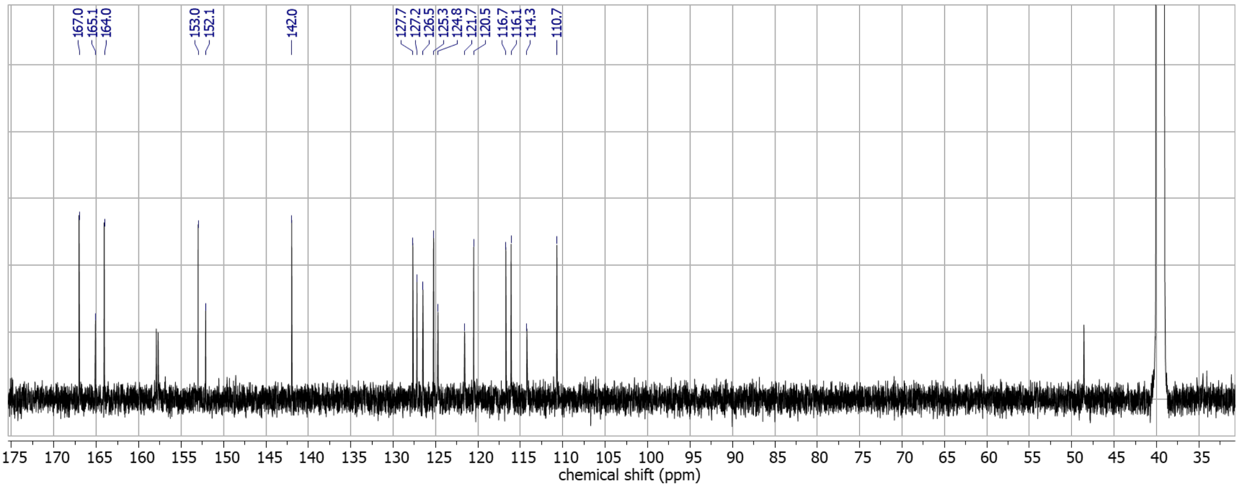

Figure S 29. ^{1}H-decoupled ^{13}C NMR spectrum (150 MHz, DMSO-d_6) of closoxazole C (**3***).

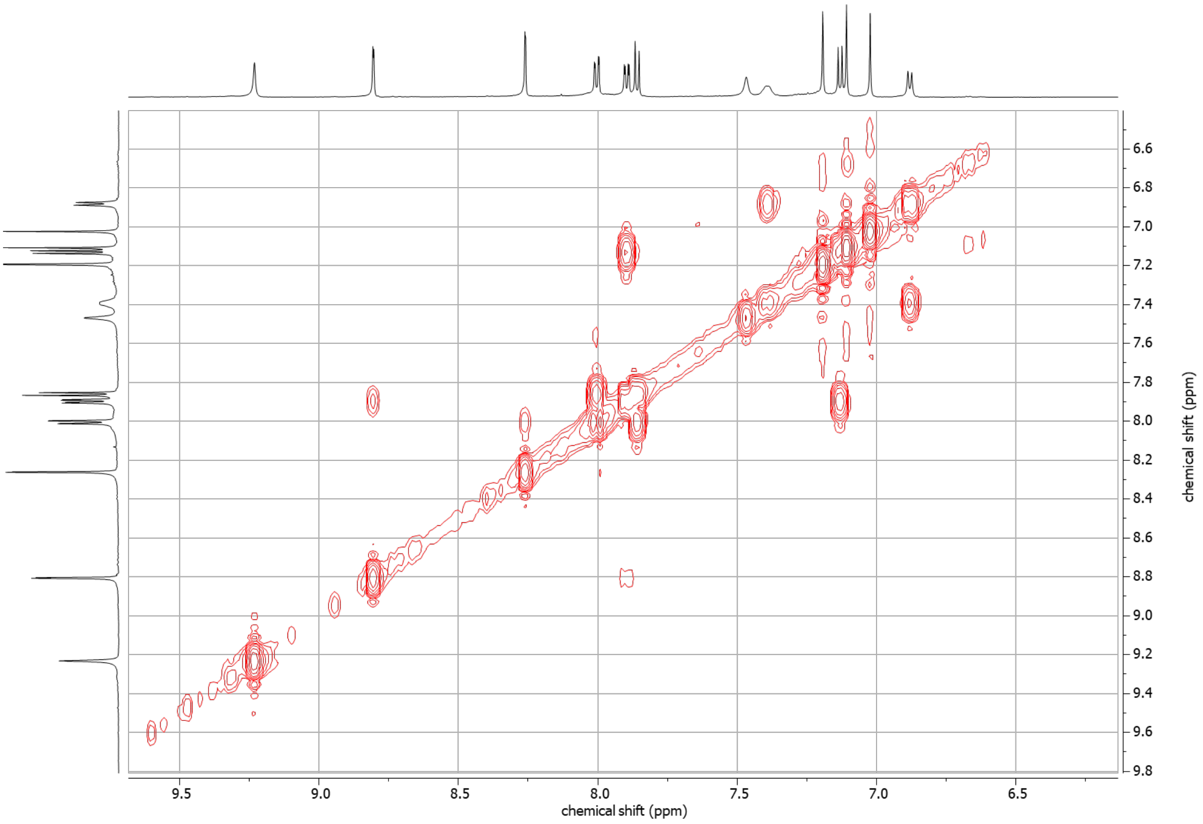

Figure S 30. COSY spectrum (DMSO-d_6) of closoxazole C (**3***).

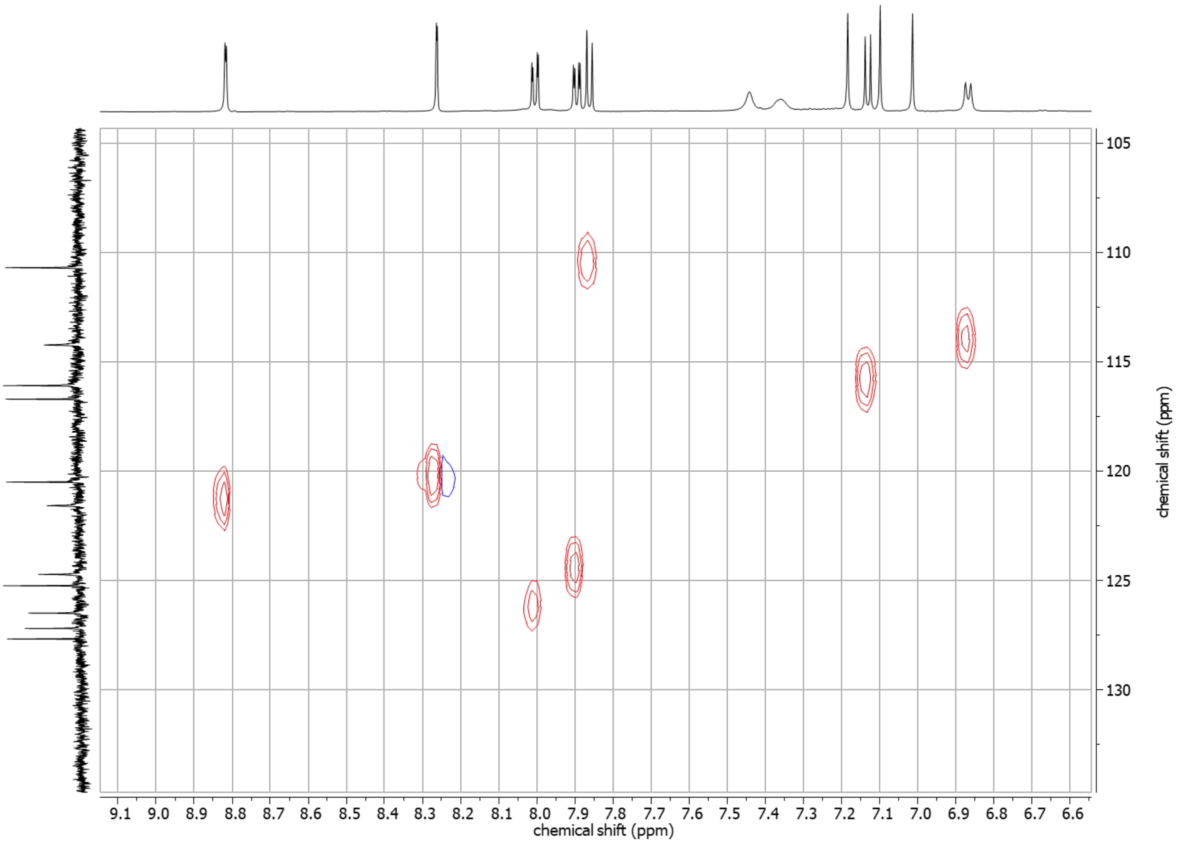

Figure S 31. HSQC spectrum (DMSO-d_6) of closoxazole C (**3***).

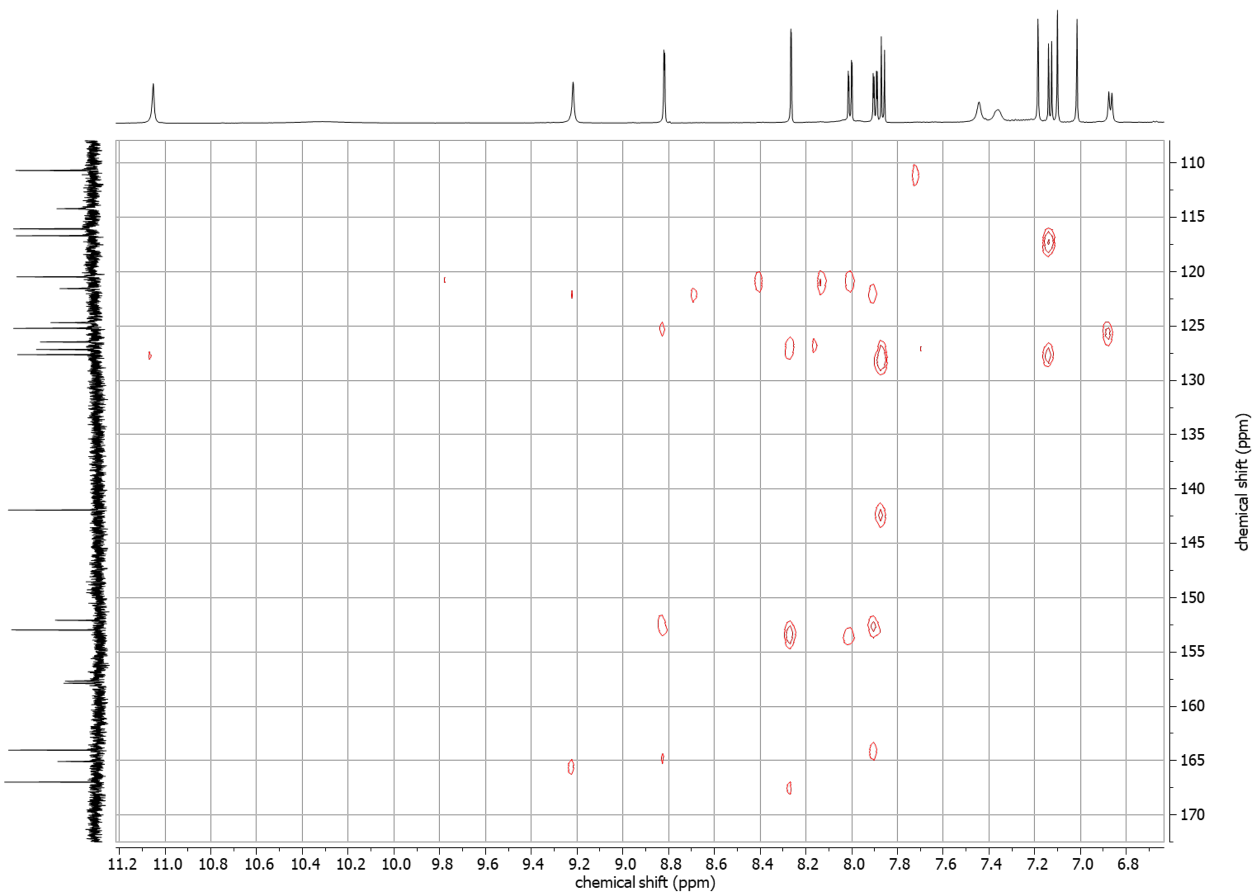

Figure S 32. HMBC spectrum (DMSO-d_6) of closoxazole C (**3***).

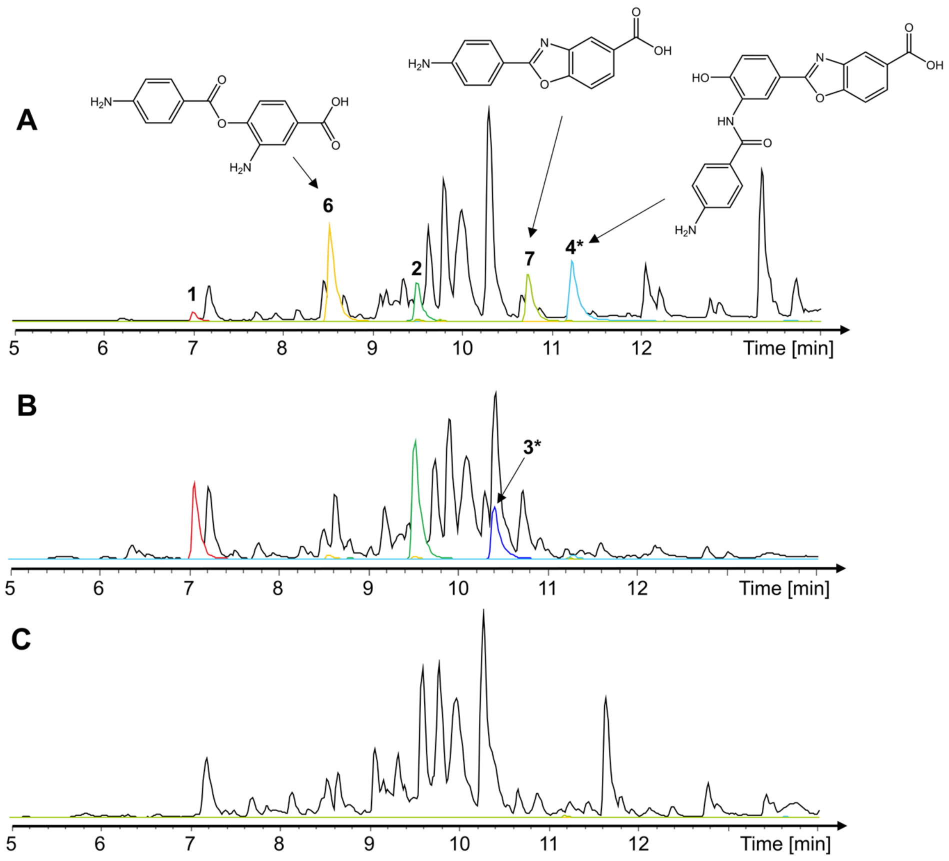

Figure S 33. (A) Raw extract of *E. coli* BL21(DE3): pET28a-*pfxBC* supplemented with 50 mg L^{-1} 3,4-AHBA and 50 mg L^{-1} 4-ABA. (B) Raw extract of *E. coli* BL21(DE3): pET28a-*pfxBC* supplemented with 50 mg L^{-1} 3,4-AHBA. (C) Raw extract of *E. coli* BL21(DE3): pET28a(+) (negative control). Red: EIC of **1** (*m/z* 289.08). Green: EIC of the **2** (*m/z* 289.09). Light blue: EIC of closoxazole D (**4***, *m/z* 390.10). Yellow: EIC of **6** (*m/z* 273.09) Light green: EIC of **7** (*m/z* 255.08).

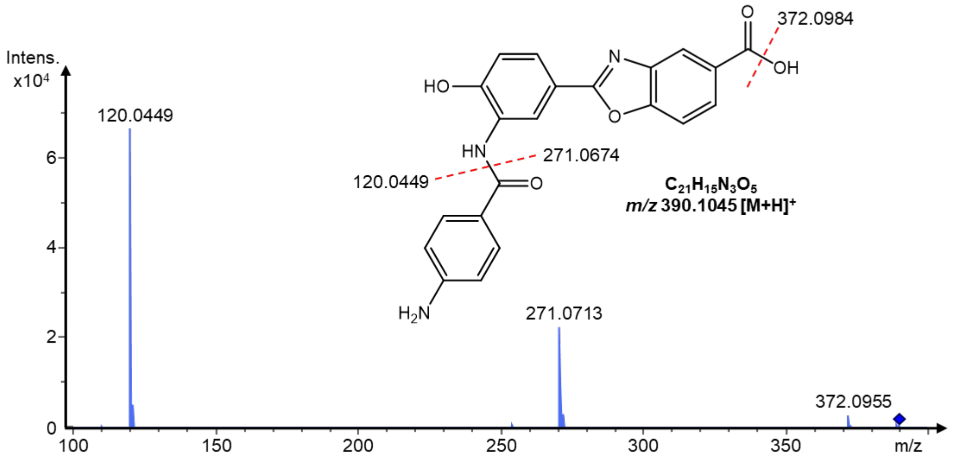

Figure S 34. LC-MS/MS fragmentation pattern of closoxazole D (**4***, *m/z* 390.10) from a raw extract of *E. coli* BL21(DE3): pET28a-*pfxBC* supplemented with 50 mg L^{-1} 3,4-AHBA and 50 mg L^{-1} 4-ABA.

Table S 6. NMR data of closoxazole D (**4***) in DMSO-d_6.

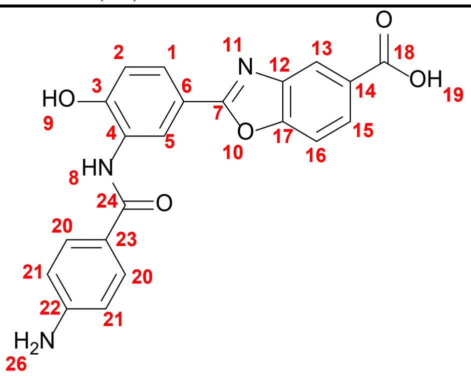

#	δ_C	type	δ_H, M (*J* in Hz)	COSY	HMBC ($^1H \rightarrow ^{13}C$)
1	124.5	CH	7.88, dd (8.4, 2.1)	2, 5	3, 5, 7
2	116.4	CH	7.12, d (8.4)	1	(3), 4, 6
3	152.2	C_q			
4	127.5	C_q			
5	121.8	CH	8.78, d (2.1)	1	1, 3, (4), 7
6	116.7	C_q			
7	164.1	C_q			
8	-	NH	9.22, s		3, 5, 24
12	141.9	C_q			
13	120.5	CH	8.26, br s	15	15, 17, 18
14	127.6	C_q			
15	126.5	CH	8.00, dt (8.5, 1.6)	16	13, 17, 18
16	110.7	CH	7.86, d (8.5)	15	12, 14
17	153.0	C_q			
18	167.0	C_q			
20	129.3	CH x2	7.74, d (8.5)	21	20, 22, 24
21	112.9	CH x2	6.66, d (8.5)	20	21, 23
22	152.3	C_q			
23	120.1	C_q			
24	165.3	C_q			
	-	OH	11.01, s		

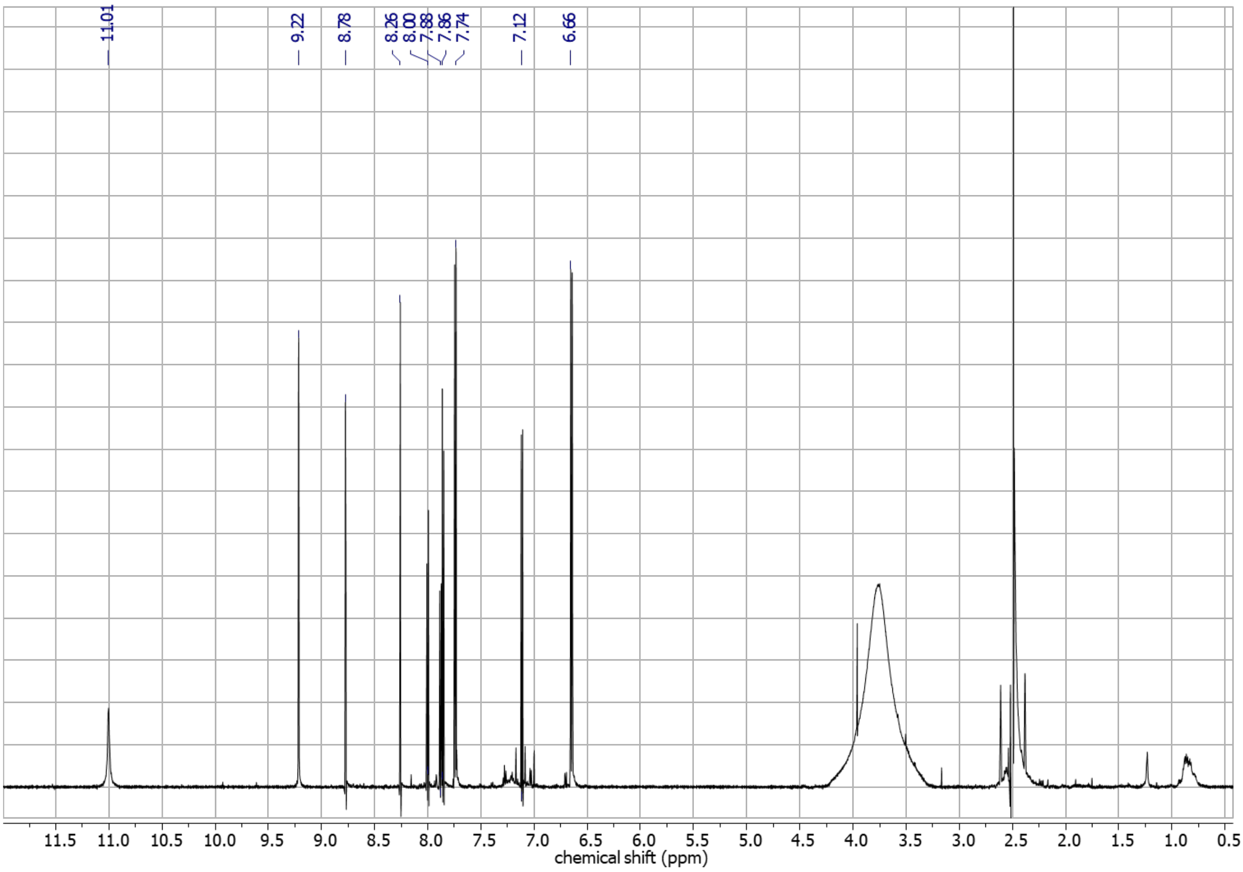

Figure S 35. ^{1}H NMR spectrum (600 MHz, DMSO-d_6) of closoxazole D (**4***).

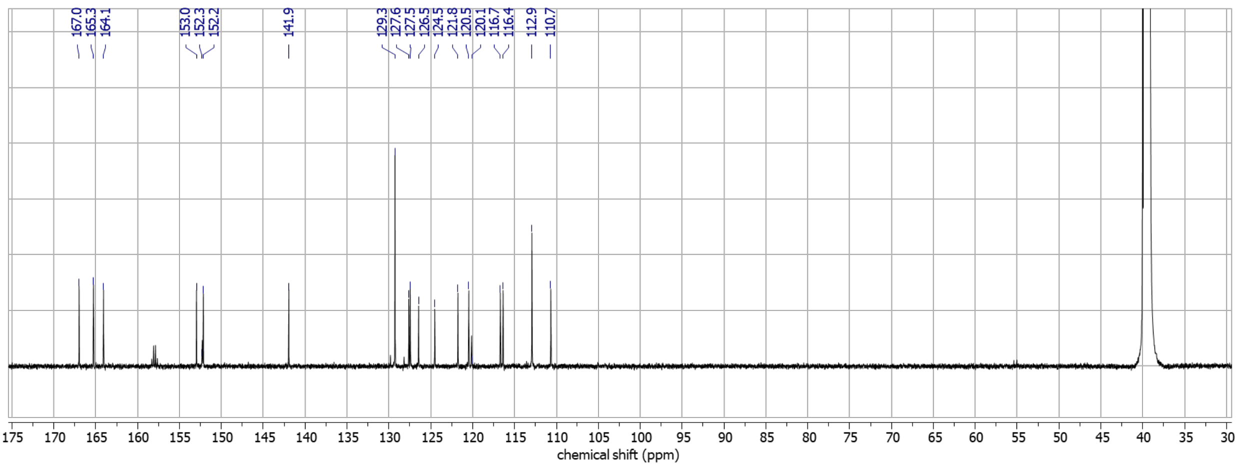

Figure S 36. ^{1}H-decoupled ^{13}C NMR spectrum (150 MHz, DMSO-d_6) of closoxazole D (**4***).

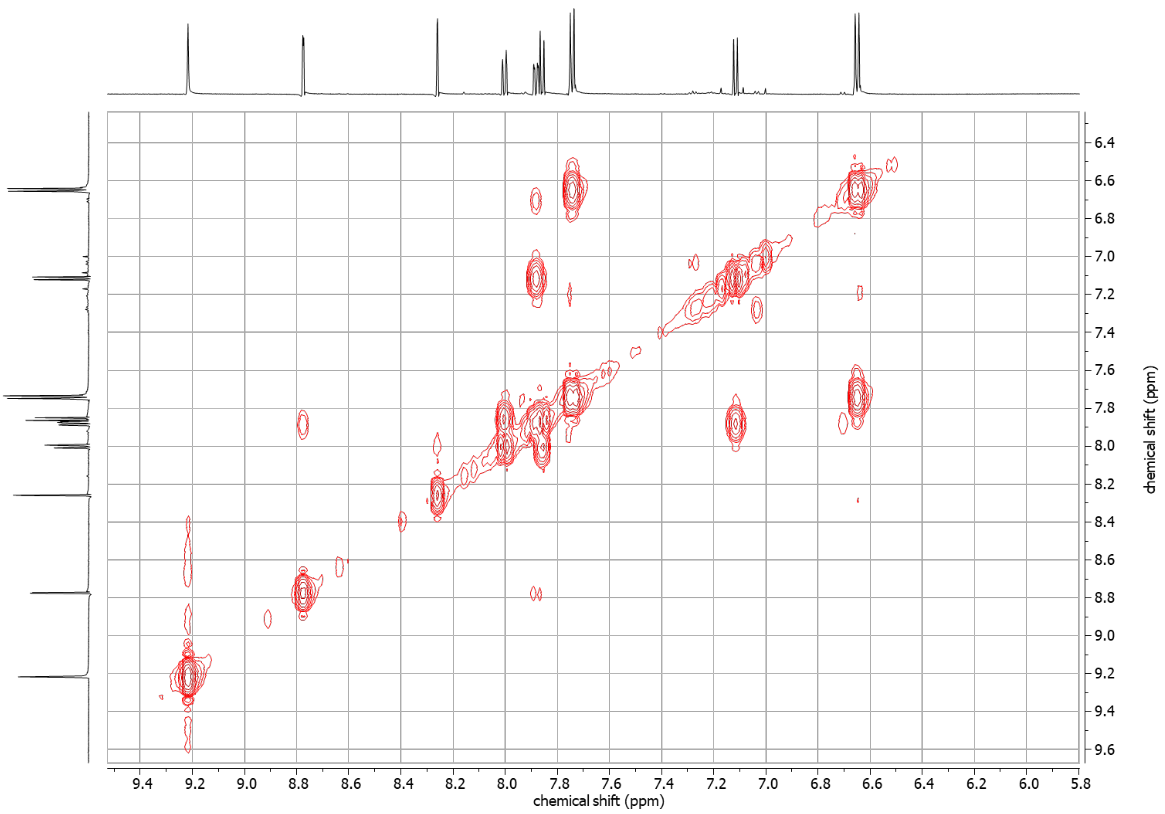

Figure S 37. COSY spectrum (DMSO-d_6) of closoxazole D (**4***).

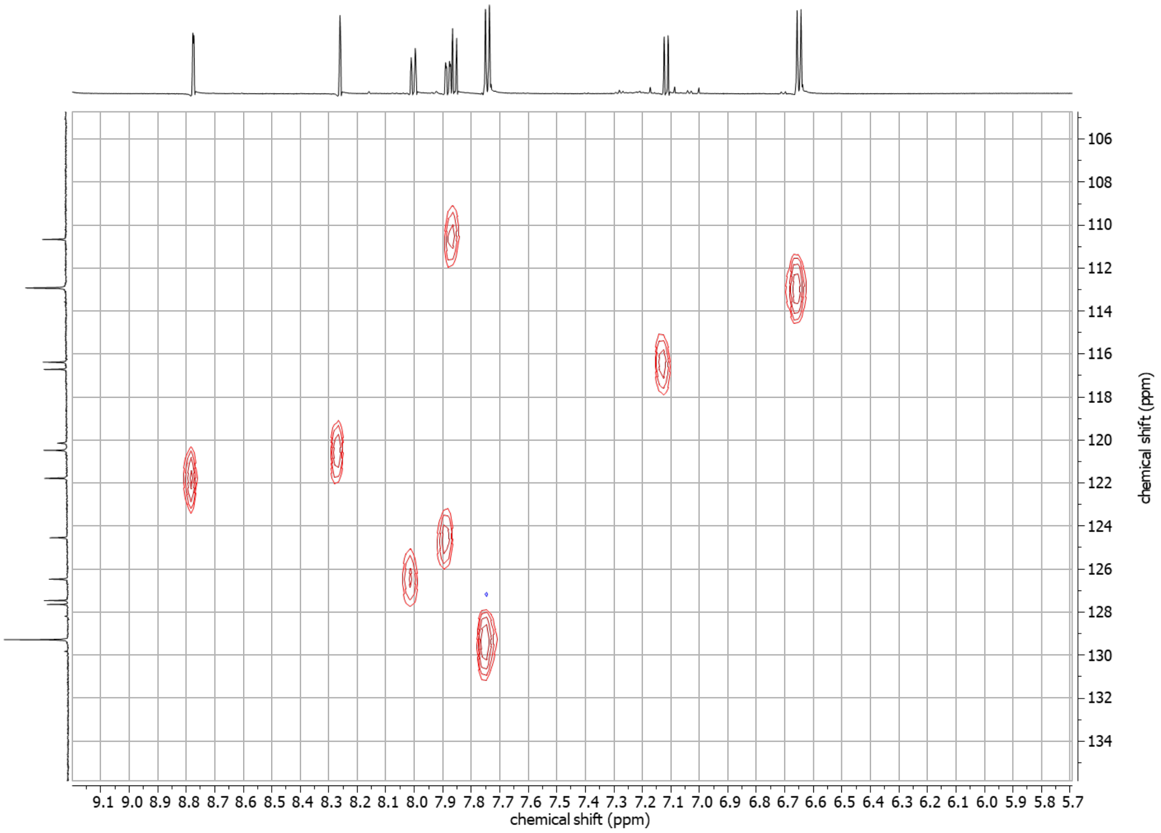

Figure S 38. HSQC spectrum (DMSO-d_6) of closoxazole D (**4***).

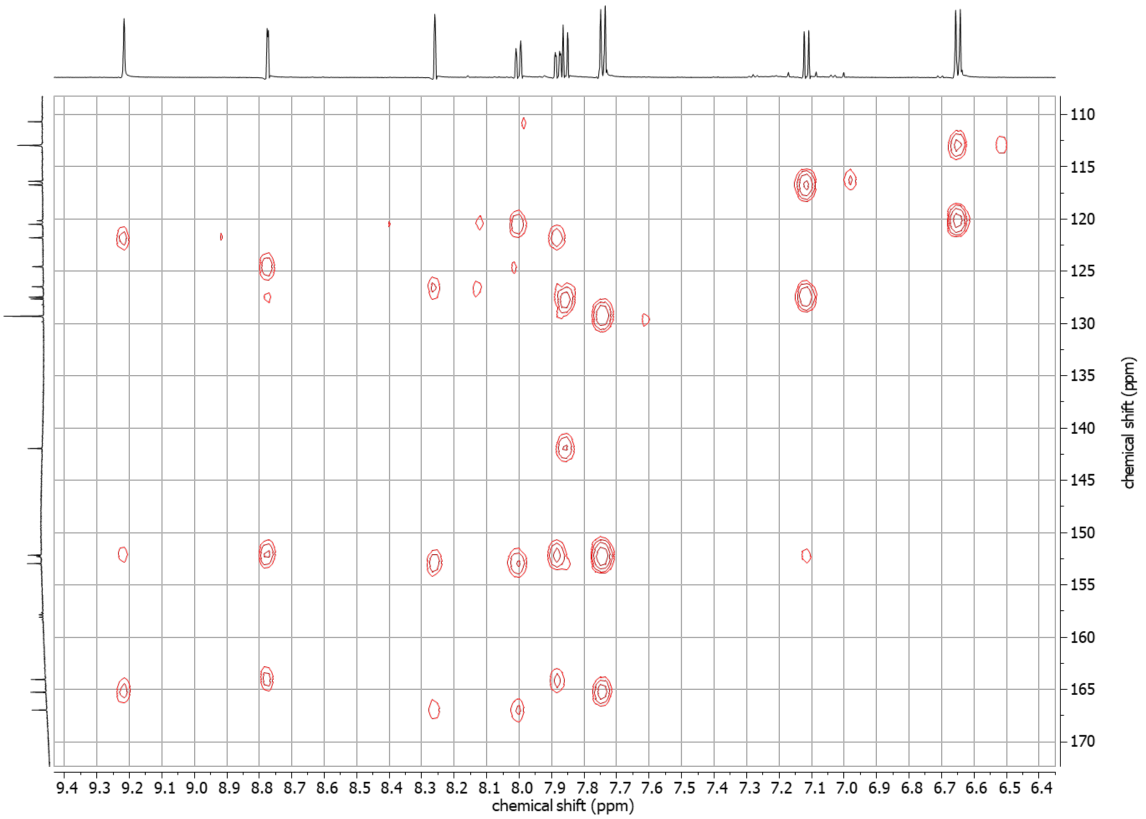

Figure S 39. HMBC spectrum (DMSO-d_6) of closoxazole D (**4***).

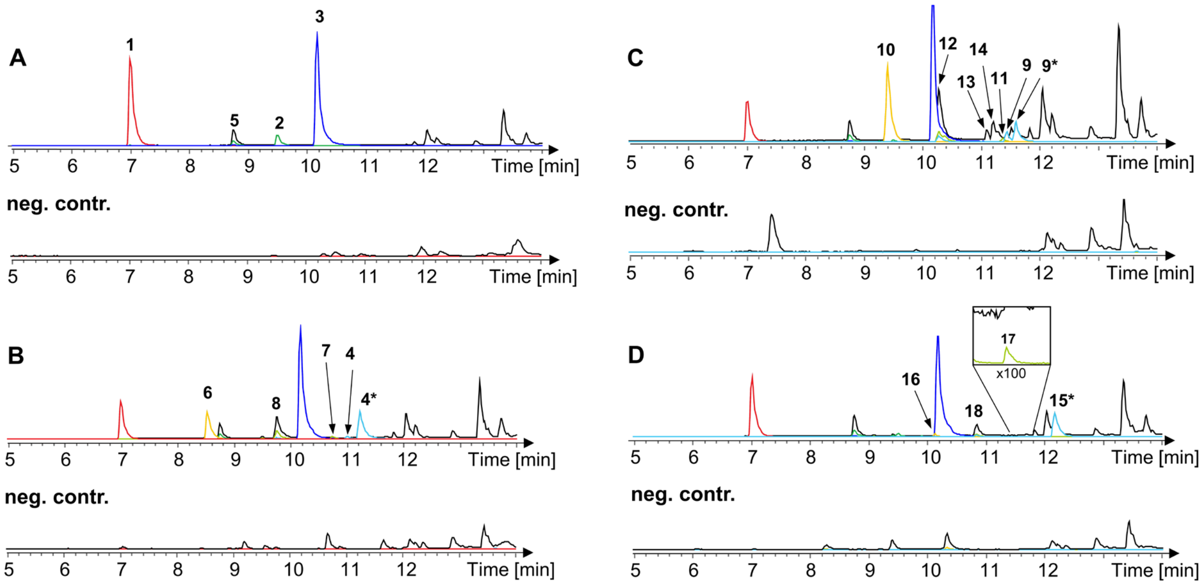

Figure S 40. LC-MS chromatograms of in vitro reactions using PfxA, PfxB and PfxC and the substrates (A) 3,4-AHBA, (B) 3,4-AHBA and 4-ABA, (C) 3,4-AHBA and 3-HBA, or (D) 3,4-AHBA and BA. Black: BPC; Colored: EICs of the respective closoxazole derivatives and the biosynthetic intermediates. Negative controls were prepared without addition of enzymes.

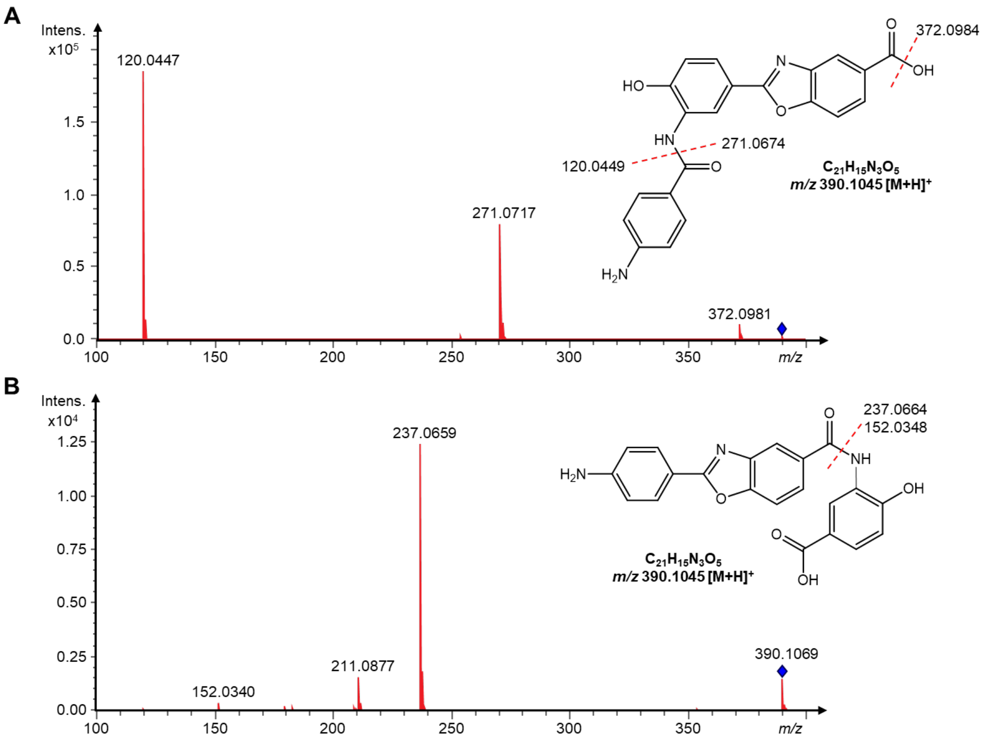

Figure S 41. LC-MS/MS fragmentation of (A) closoxazole D (**4***, *m/z* 390.10) und (B) closoxazole B (**4**, *m/z* 390.10) from in vitro reactions containing PfxA, PfxB and PfxC and 3,4-AHBA and 4-ABA as substrates. The fragmentation pattern of **4** is consistent with the previously published fragmentation pattern for closoxazole B.[9]

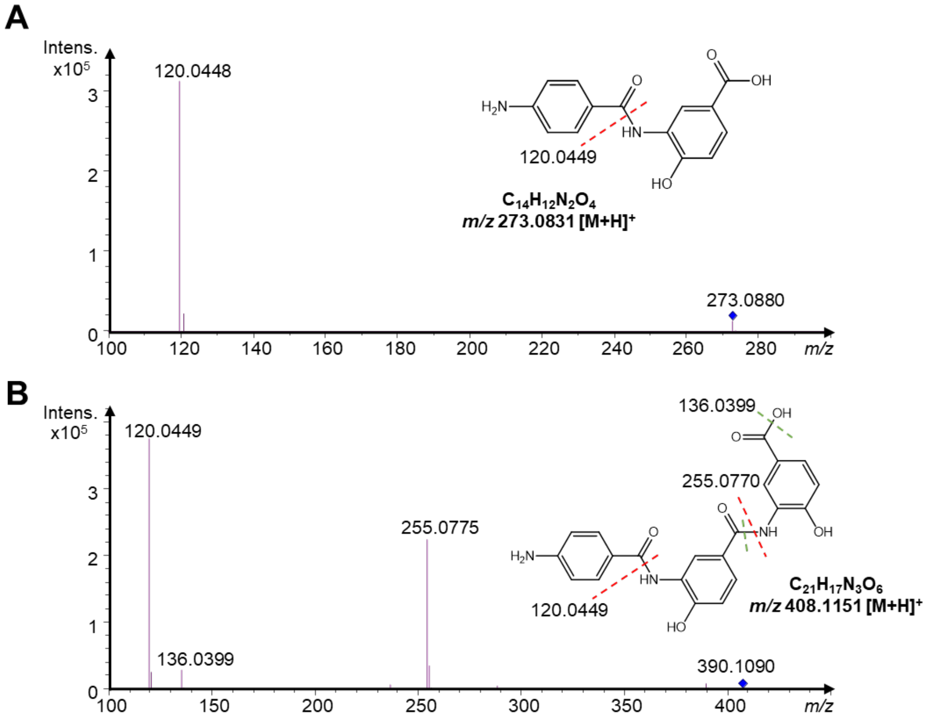

Figure S 42. LC-MS/MS fragmentation patterns of (A) **6** (*m/z* 273.08) and (B) **8** (*m/z* 408.11) from in vitro assays using the enzymes PfxA, PfxB and PfxC and the substrates 3,4-AHBA and 4-ABA.

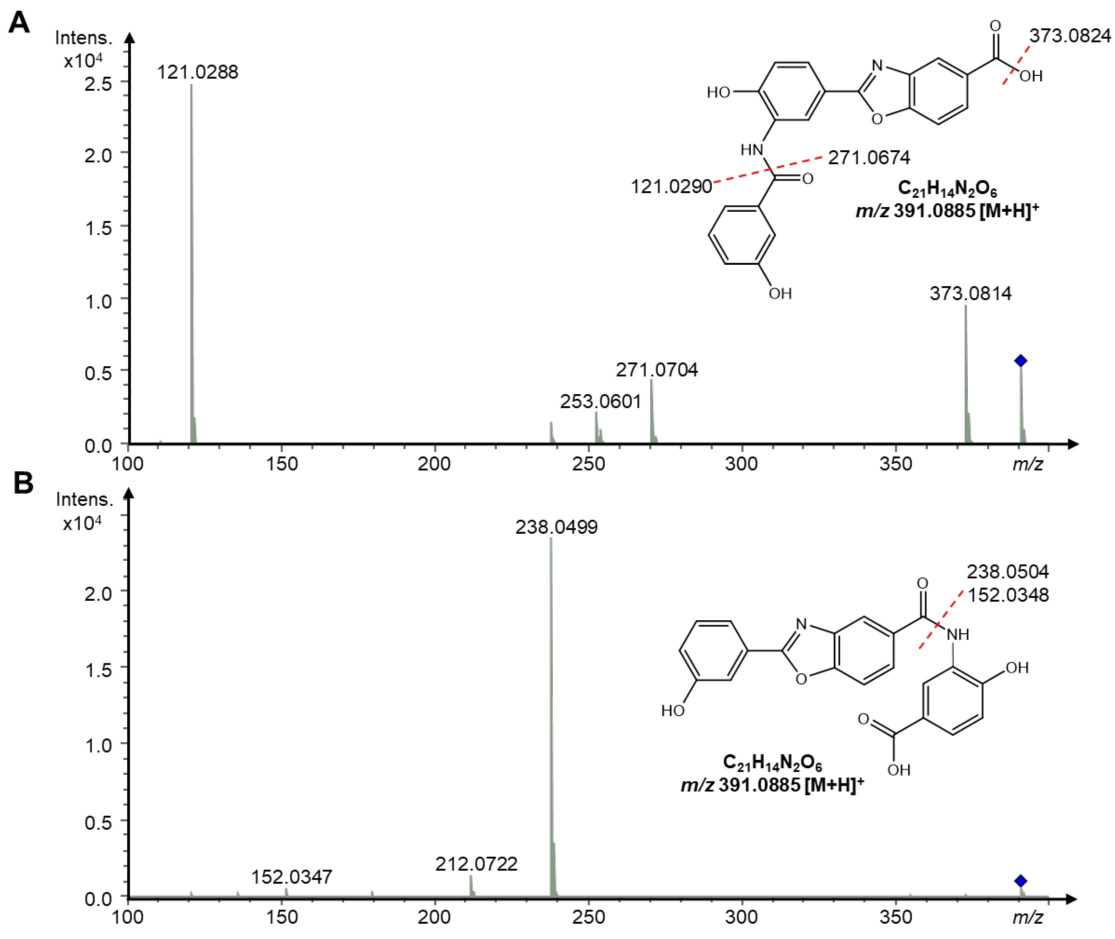

Figure S 43. LC-MS/MS fragmentation patterns of (A) **9*** (*m/z* 391.09) and (B) **9** (*m/z* 391.09) isolated from in vitro assays using the enzymes PfxA, PfxB and PfxC and the substrates 3,4-AHBA and 3-HBA.

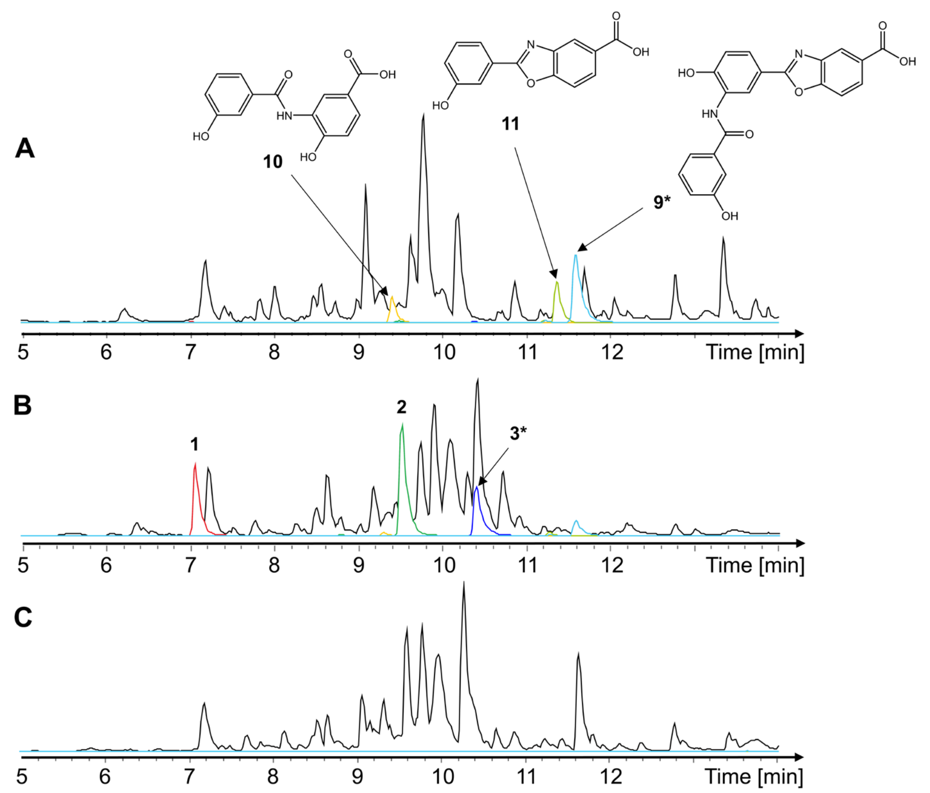

Figure S 44. (A) Raw extract of *E. coli* BL21(DE3): pET28a-*pfxBC* supplemented with 50 mg L^{-1} 3,4-AHBA and 50 mg L^{-1} 3-HBA. (B) Raw extract of *E. coli* BL21(DE3): pET28a-*pfxBC* supplemented with 50 mg L^{-1} 3,4-AHBA. (C) Raw extract of *E. coli* BL21(DE3): pET28a(+) culture (negative control). Light blue: EIC of closoxazole E (**9***, *m/z* 391.09). Yellow: EIC of **10** (*m/z* 274.07). Light green: EIC of **11** (*m/z* 256.06).

Table S 7. NMR data of closoxazole E (**9***) in DMSO-d_6.

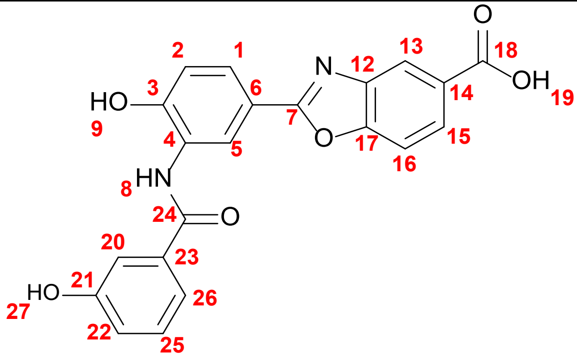

#	δ_C	type	δ_H, M (*J* in Hz)	COSY	HMBC ($^1H \rightarrow ^{13}C$)
1	125.3	CH	7.93, dd (8.5, 2.2)	2, 5	3, 5, 7
2	116.2	CH	7.14, d (8.5)	1	4, 6
3	152.8	C_q			
4	126.5	C_q			
5	122.5	CH	8.75, d (2.2)	1, 8	1, 3, 7
6	116.6	C_q			
7	164.0	C_q			
8	-	NH	9.43, s		3, 5, 24
9	-	OH	10.98, s		(3), 4
12	141.9	C_q			
13	120.5	CH	8.26, d (1.8)	15	15, 17, 18
14	127.7	C_q			
15	126.7	CH	8.00, dd (8.5, 1.8)	(13), 16	13, 17, 18
16	110.7	CH	7.86, d (8.5)	15	12, 14
17	153.0	C_q			
18	167.0	C_q			
20	114.3	CH	7.37, dd (2.6, 1.5)	22, 26	24, 26
21	157.5	C_q			
22	118.9	CH	7.00, ddd (8.0, 2.6, 1.0)	25	20, 26
23	135.6	C_q			
24	165.2	C_q			
25	129.7	CH	7.35, t (8.0, 7.7)	22, 26	21, 23
26	117.9	CH	7.43, dt (7.7, 1.4)	22, 25	20, 22, 24
27	-	OH	9.81, s		20, 21, 22
		OH	13.08, br s		

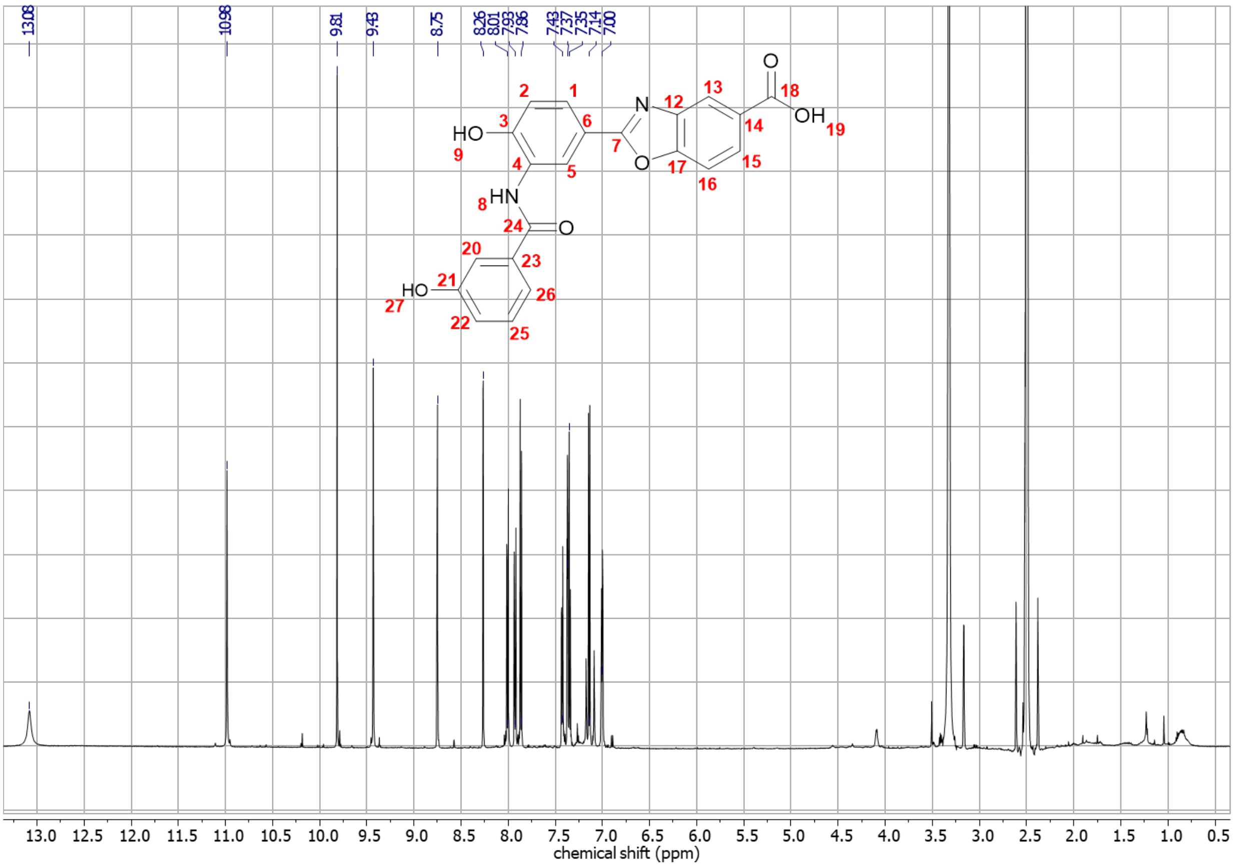

Figure S 45. ^{1}H NMR spectrum (600 MHz, DMSO-d_6) of closoxazole E (**9***).

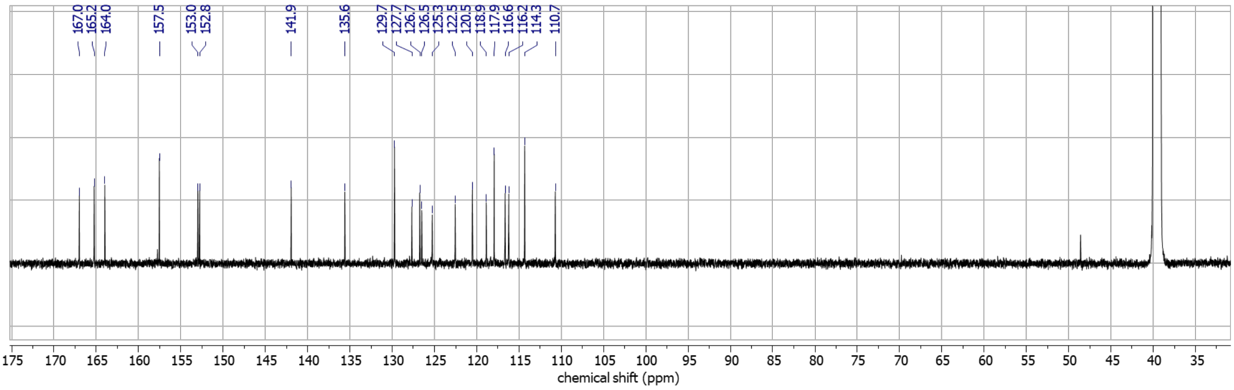

Figure S 46. ^{1}H-decoupled ^{13}C NMR spectrum (150 MHz, DMSO-d_6) of closoxazole E (**9***).

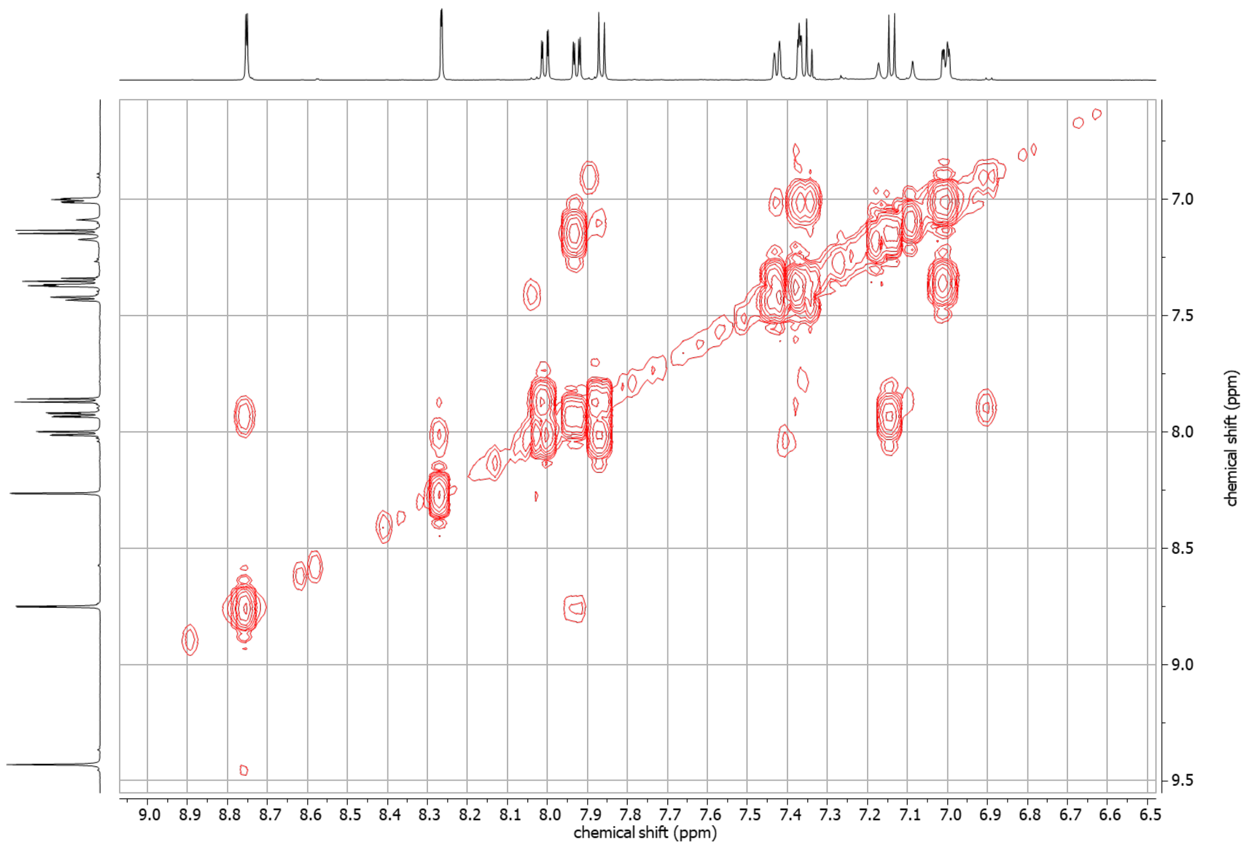

Figure S 47. COSY spectrum (DMSO-d_6) of closoxazole E (**9***).

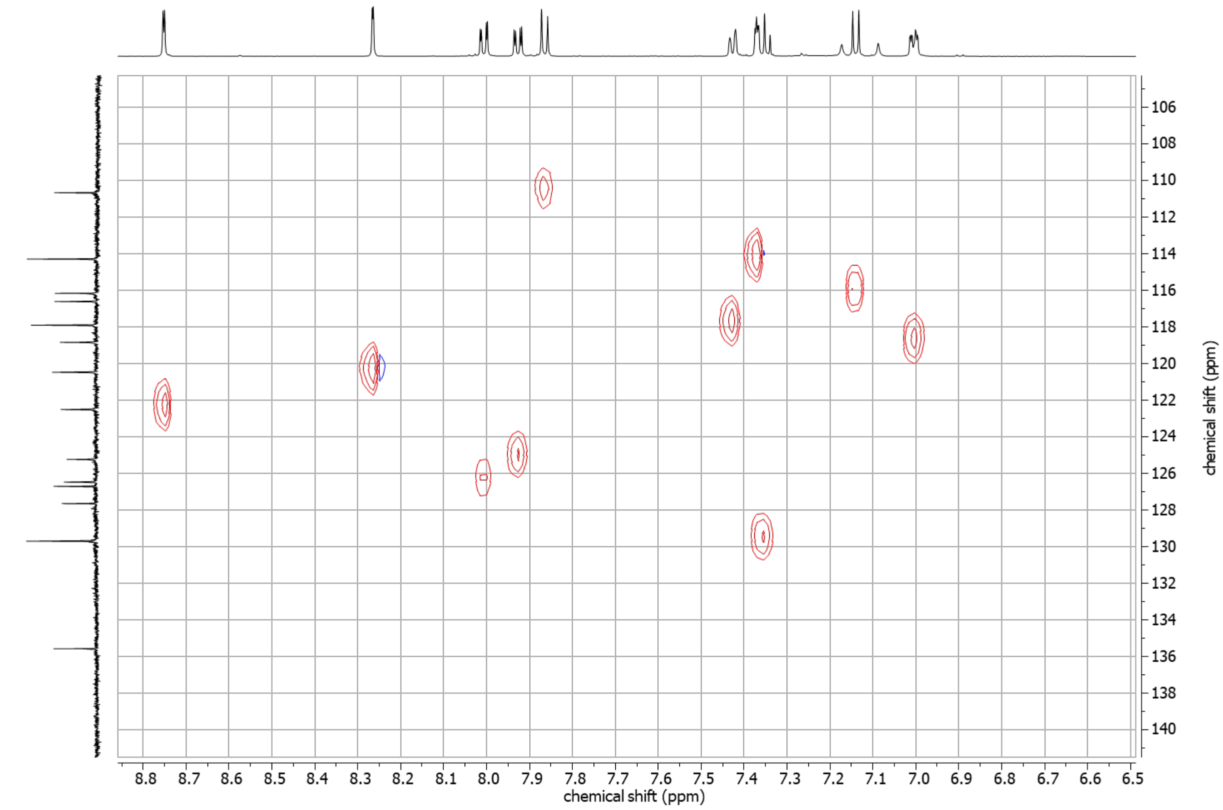

Figure S 48. HSQC spectrum (DMSO-d_6) of closoxazole E (**9***).

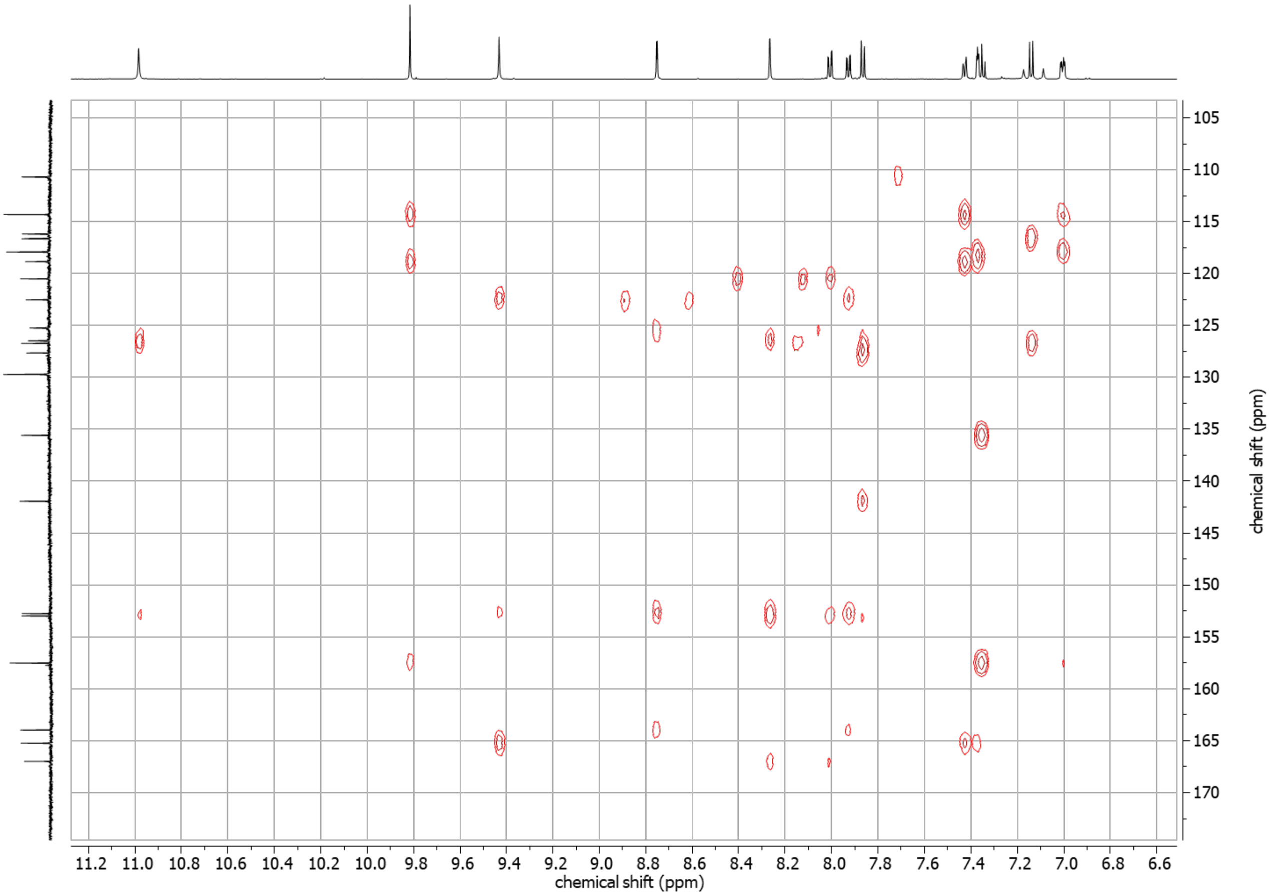

Figure S 49. HMBC spectrum (DMSO-d_6) of closoxazole E (**9***).

Table S 8. NMR data of **11** in DMSO-d_6.

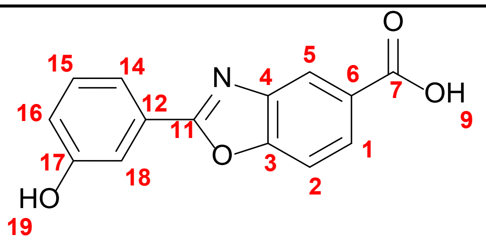

#	δ_C	type	δ_H, M (J in Hz)	COSY	HMBC ($^1H \rightarrow ^{13}C$)
1	127.0	CH	8.04, dd (8.5, 1.7)	2, 5	(2), 3, 5, 7
2	111.0	CH	7.89, d (8.5)	1	(3), 4, 6
3	153.0	C_q			
4	141.6	C_q			
5	121.0	CH	8.31, d (1.7)	1	1, 3, 7
6	128.0*	C_q			
7	167.0*	C_q			
9	-	OH	13.12, s		
11	163.7	C_q			
12	127.1	C_q			
14	118.3	CH	7.66, ddd (7.6, 1.6, 1.0)	15	11, 16, 18
15	130.6	CH	7.43, t (8.1, 7.9)	14, 16	12, 17
16	119.6	CH	7.05, ddd (8.1, 2.6, 1.0)	15, 18	14, 18
17	157.9	C_q			
18	113.8	CH	7.61, dd (2.6, 1.6)	16	11, 14, 16
19	-	OH	10.00, s		16, 17, 18

* Chemical shifts were deduced from HSQC or HMBC spectra.

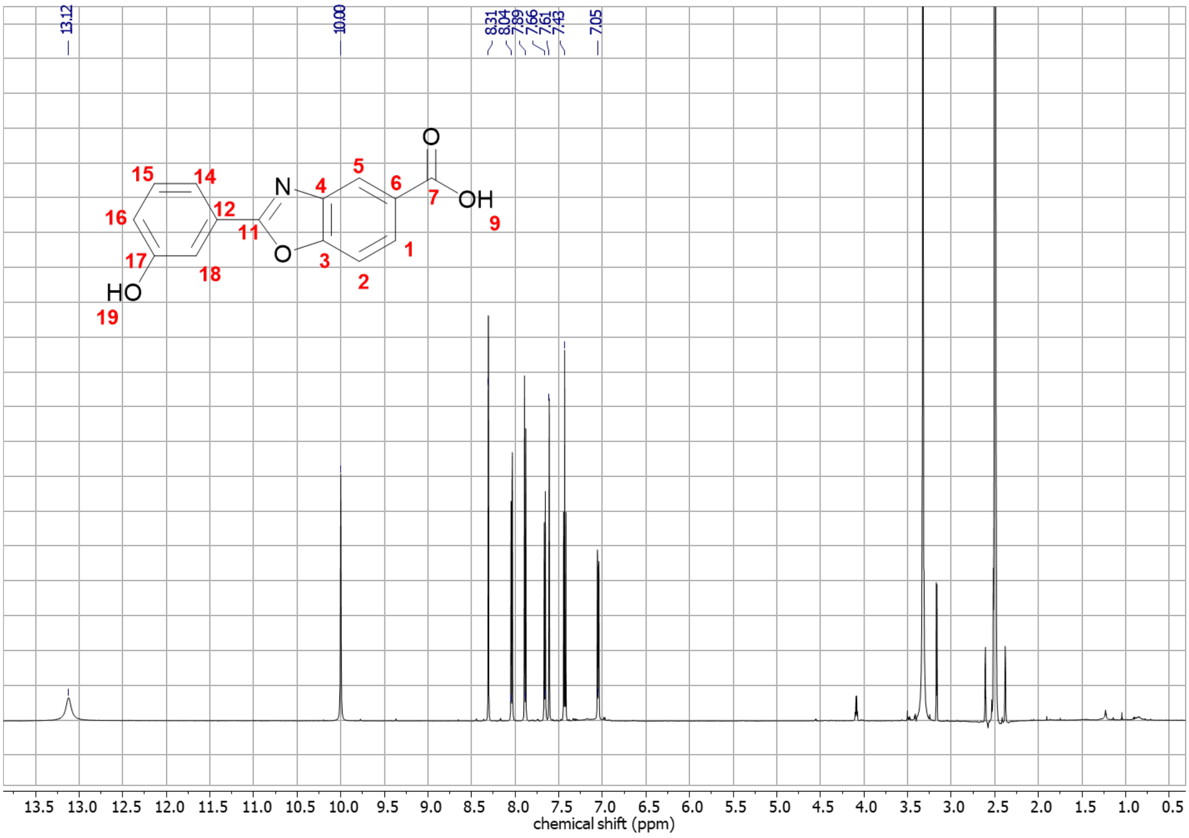

Figure S 50. ^{1}H NMR spectrum (600 MHz, DMSO-d_6) of **11**.

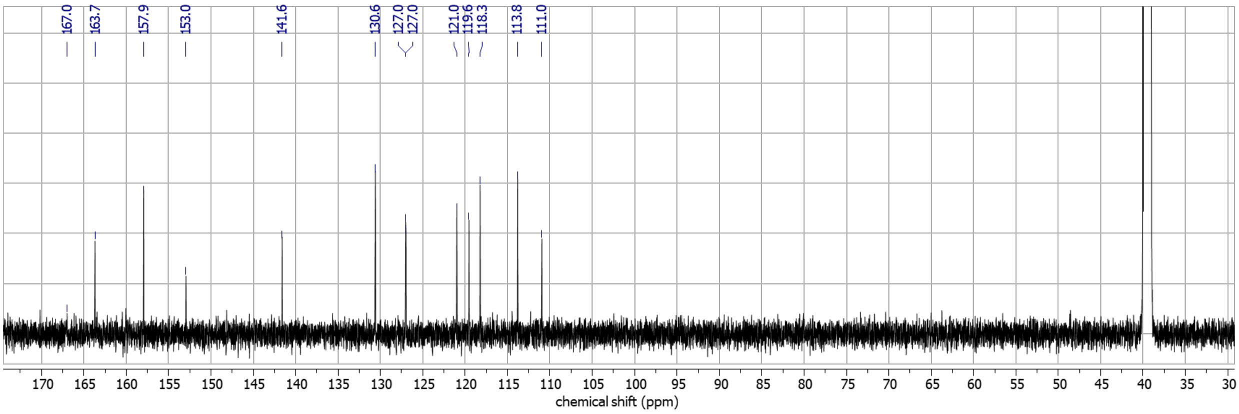

Figure S 51. ^{1}H-decoupled ^{13}C NMR spectrum (150 MHz, DMSO-d_6) of **11**.

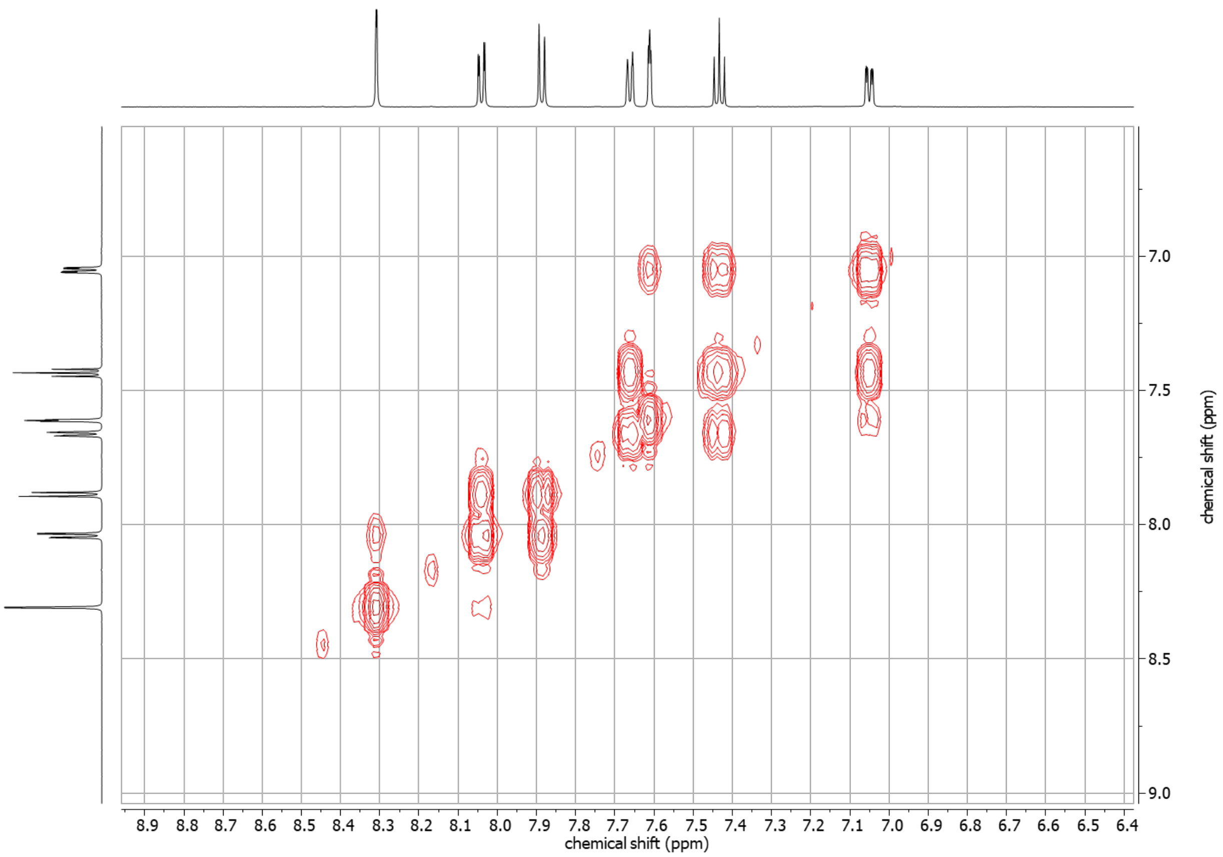

Figure S 52. COSY spectrum (DMSO-d_6) of **11**.

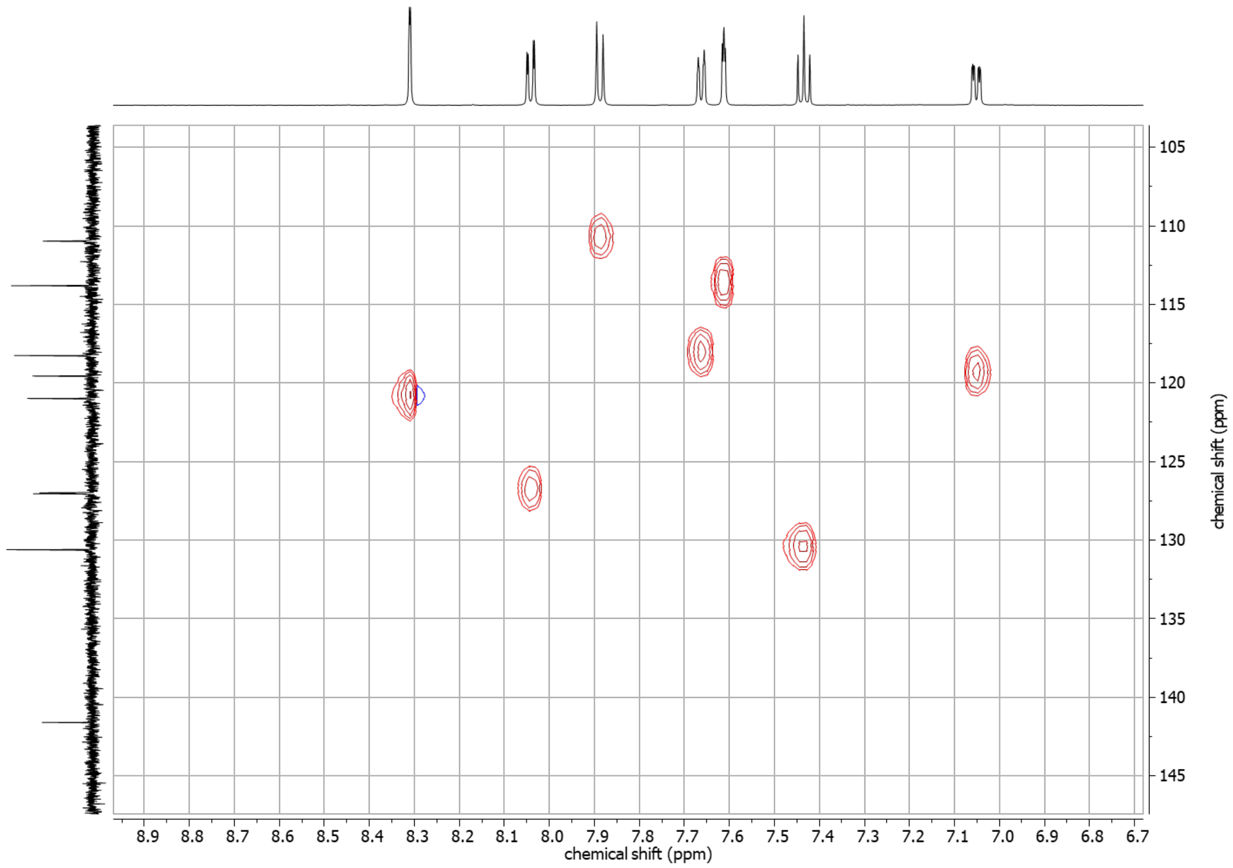

Figure S 53. HSQC spectrum (DMSO-d_6) of **11**.

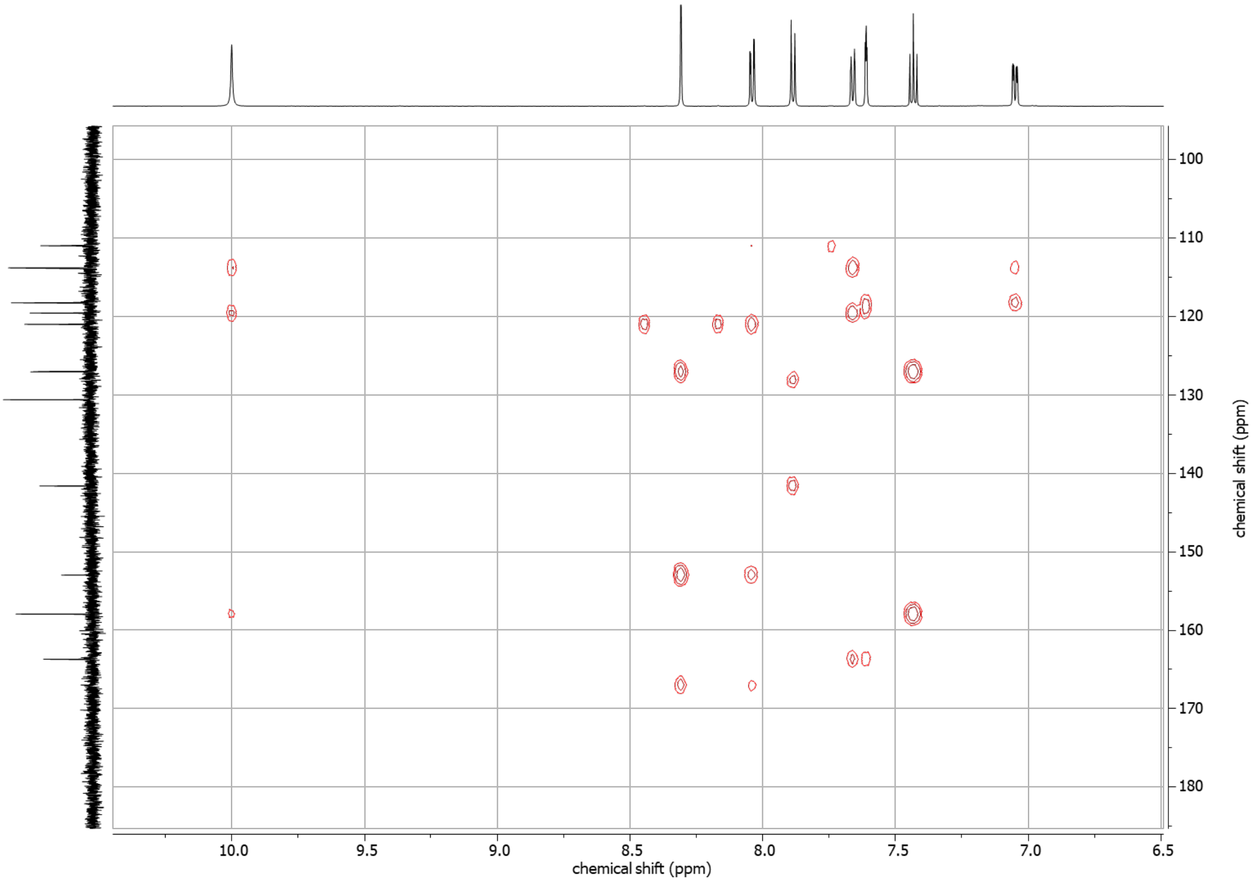

Figure S 54. HMBC spectrum (DMSO-d_6) of **11**.

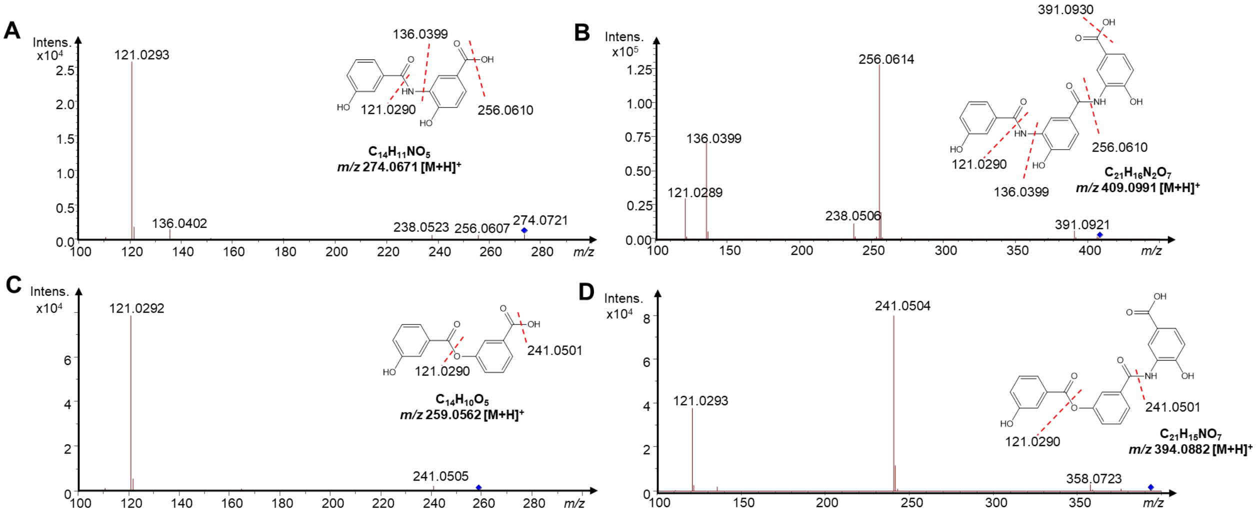

Figure S 55. LC-MS/MS fragmentation patterns of (A) **10** (*m/z* 274.07), (B) **12** (*m/z* 409.10), (C) **13** (*m/z* 259.06), and (D) **14** (*m/z* 394.09), from in vitro assays using the enzymes PfxA, PfxB and PfxC and the substrates 3,4-AHBA and 3-HBA.

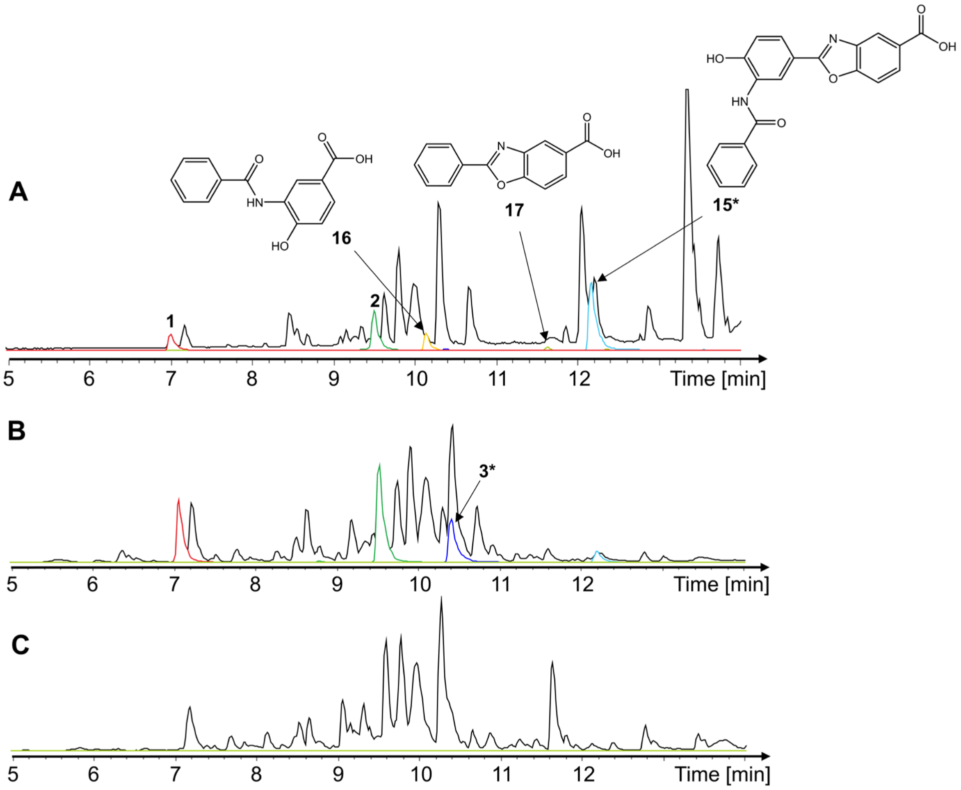

Figure S 56. (A) Raw extract of *E. coli* BL21(DE3): pET28a-*pfxBC* supplemented with 50 mg L^{-1} 3,4-AHBA and 50 mg L^{-1} BA. (B) Raw extract of *E. coli* BL21(DE3): pET28a-*pfxBC* supplemented with 50 mg L^{-1} 3,4-AHBA. (C) Raw extract of *E. coli* BL21(DE3): pET28a(+) (negative control). Green: EIC of **2** (*m/z* 271.08). Light blue: EIC of **15*** (*m/z* 375.09). Yellow: EIC of **16** (*m/z* 258.07). Light green: EIC of **17** (*m/z* 240.06).

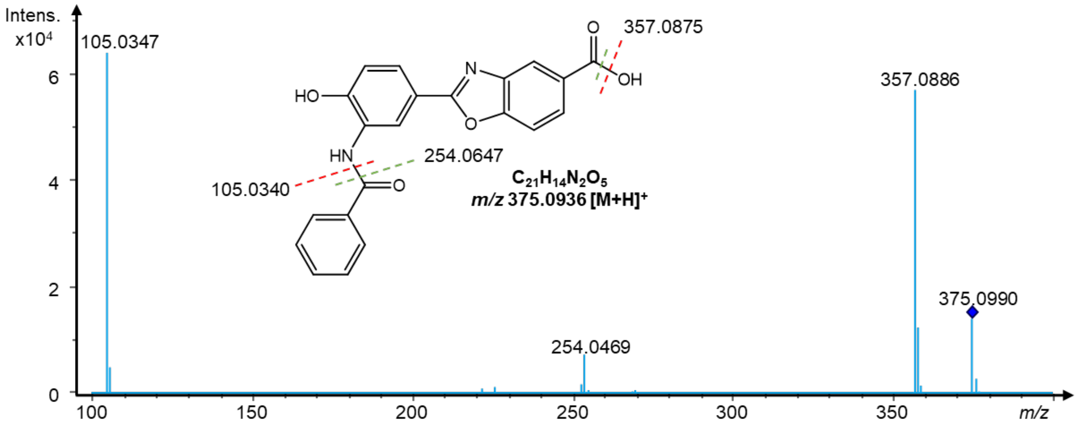

Figure S 57. LC-MS/MS fragmentation pattern of **15*** (*m/z* 375.09) from an in vitro assay using the enzymes PfxA, PfxB and PfxC and the substrates 3,4-AHBA and BA.

Table S 9. NMR data of closoxazole F (**15***) in DMSO-d_6.

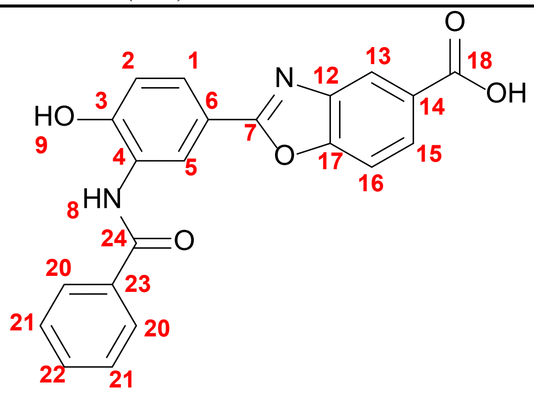

#	δ_C	type	δ_H, M (J in Hz)	COSY	HMBC ($^1H \rightarrow ^{13}C$)
1	125.5	CH	7.94, dd (8.5, 2.2)	2, 5	3, 5, 7
2	116.3	CH	7.15, d (8.5)	1	4, 6
3	153.1	C_q			
4	126.6	C_q			
5	123.1	CH	8.72, d (2.2)	1	1, 3, 7
6	116.6	C_q			
7	164.0	C_q			
8		NH	9.60, s		3, 5, 24
9		OH	10.95, s		2, (3), 4
12	141.9	C_q			
13	120.5	CH	8.26, d (1.8)	15	15, 17, 18
14	127.7	C_q			
15	126.5	CH	8.00, dd (8.5, 1.8)	16	13, 17
16	110.7	CH	7.86, d (8.5)	15	12, 14
17	153.0	C_q			
18	167.0	C_q			
20	127.6	CH x2	8.01, dd (8.5, 1.6)	21	20, 22, 24
21	128.6	CH x2	7.56, t (8.5, 7.4)	20, 22	21, 23
22	131.9	CH	7.63, dt (7.4, 1.6)	20, 21	20
23	134.2	C_q			
24	165.4	C_q			
		OH	13.09, br s		

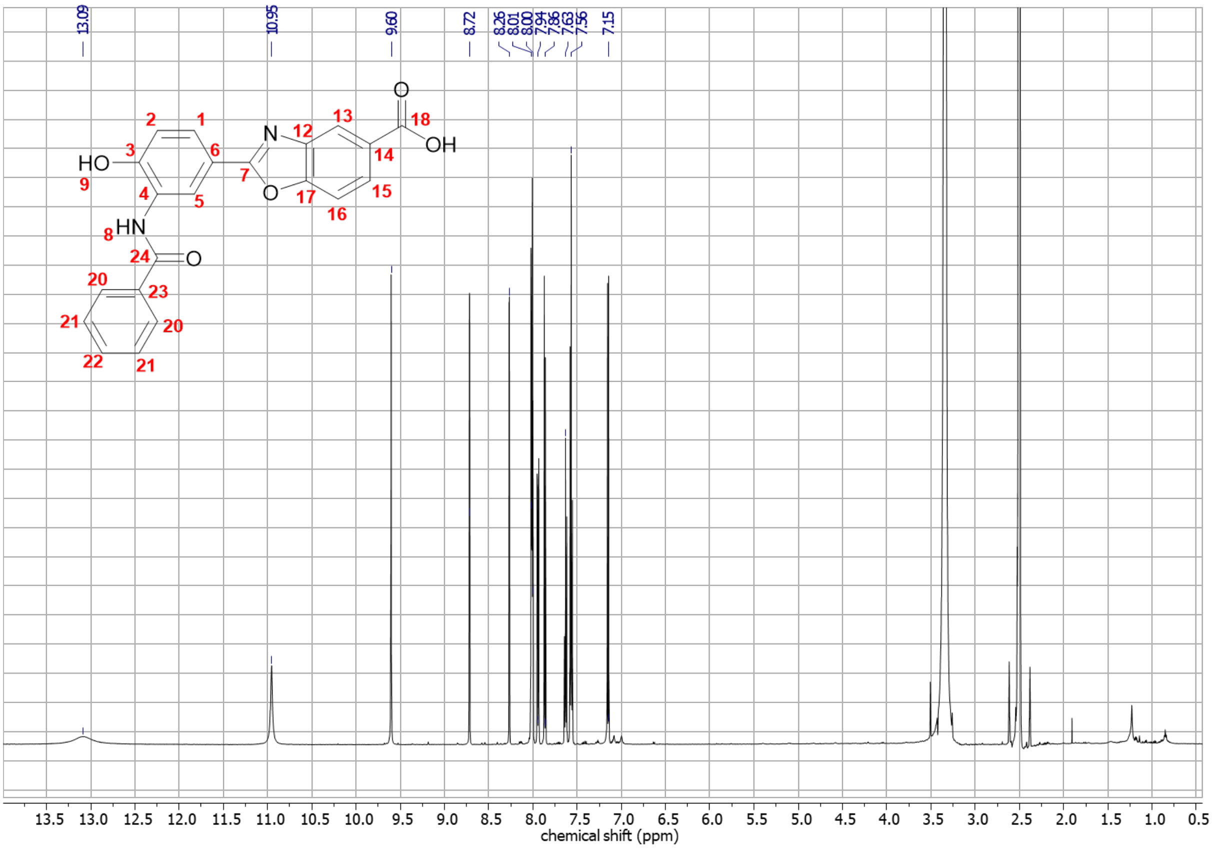

Figure S 58. ^{1}H NMR spectrum (600 MHz, DMSO-d_6) of closoxazole F (**15***).

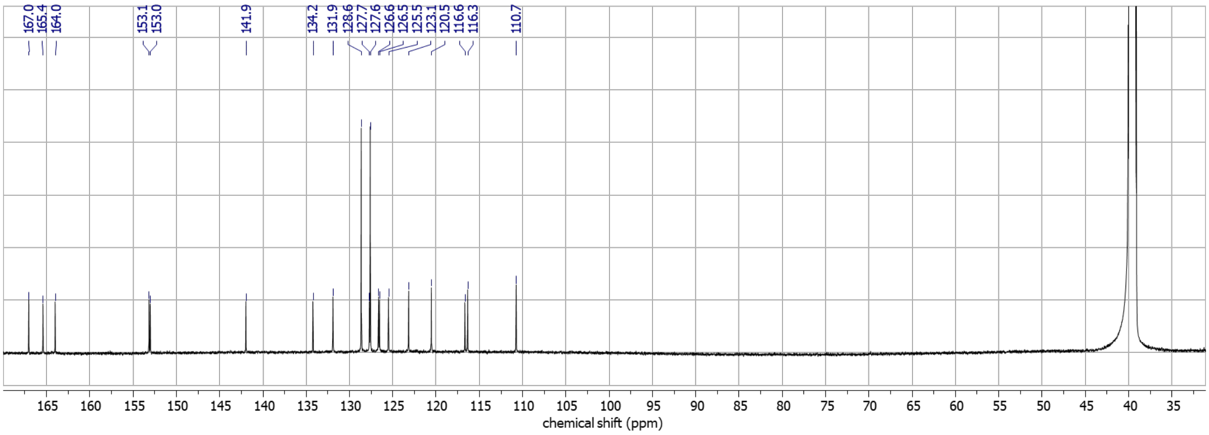

Figure S 59. ^{1}H-decoupled ^{13}C NMR spectrum (150 MHz, DMSO-d_6) of closoxazole F (**15***).

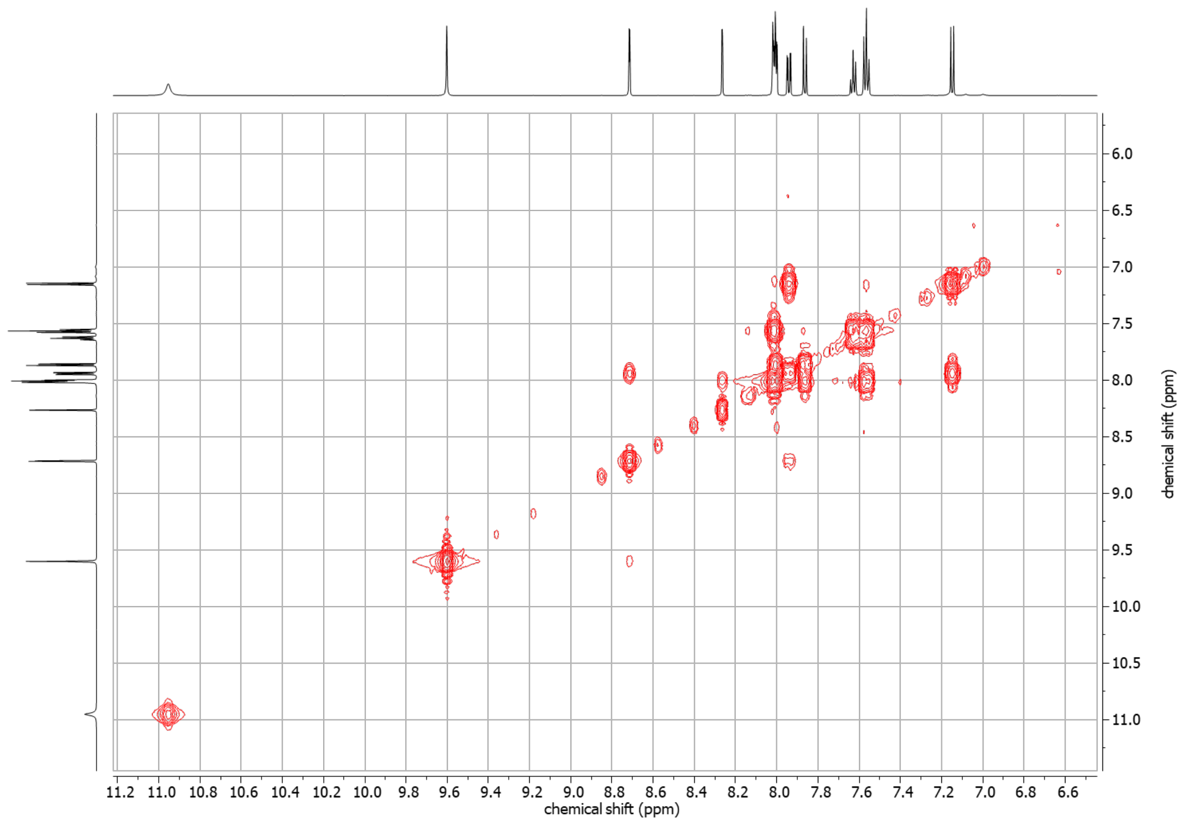

Figure S 60. COSY spectrum (DMSO-d_6) of closoxazole F (**15***).

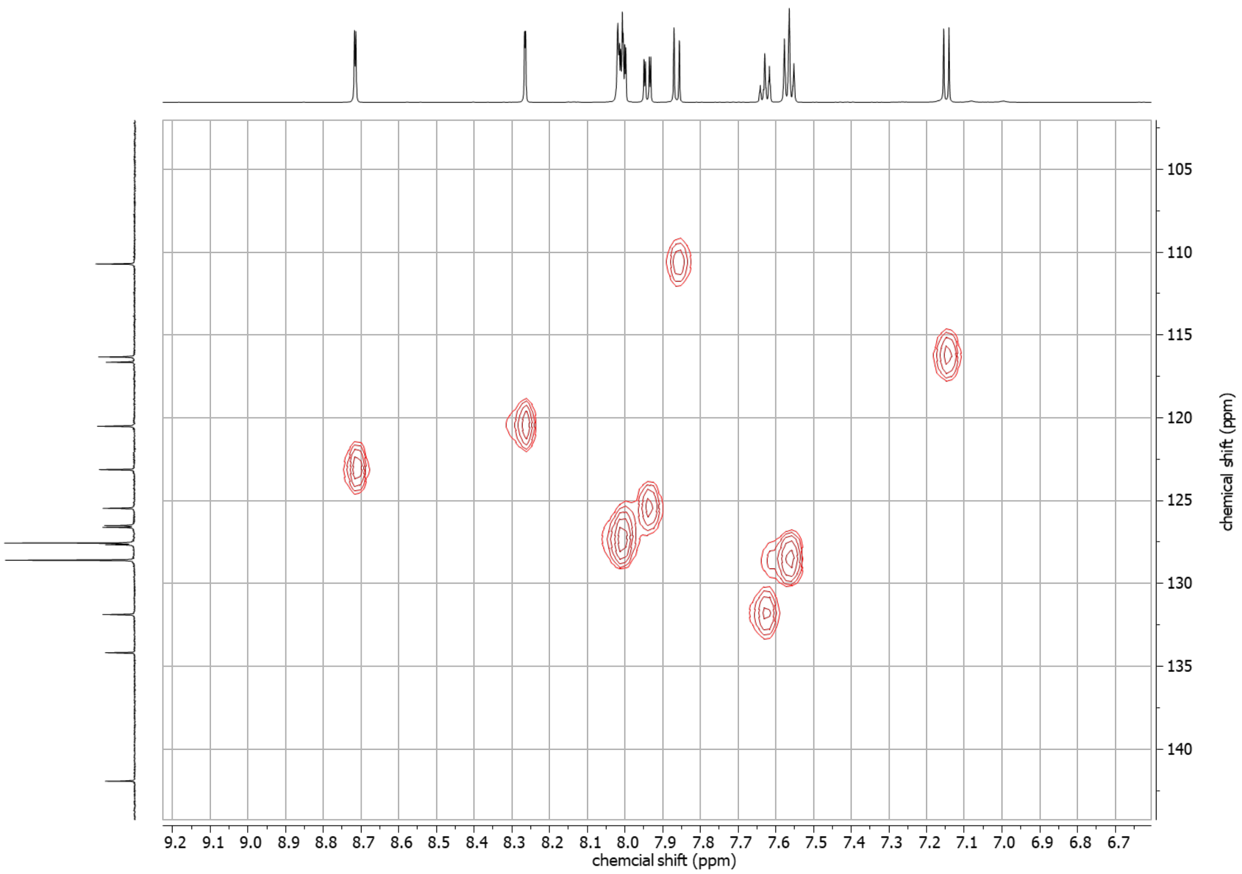

Figure S 61. HSQC spectrum (DMSO-d_6) of closoxazole F (**15***).

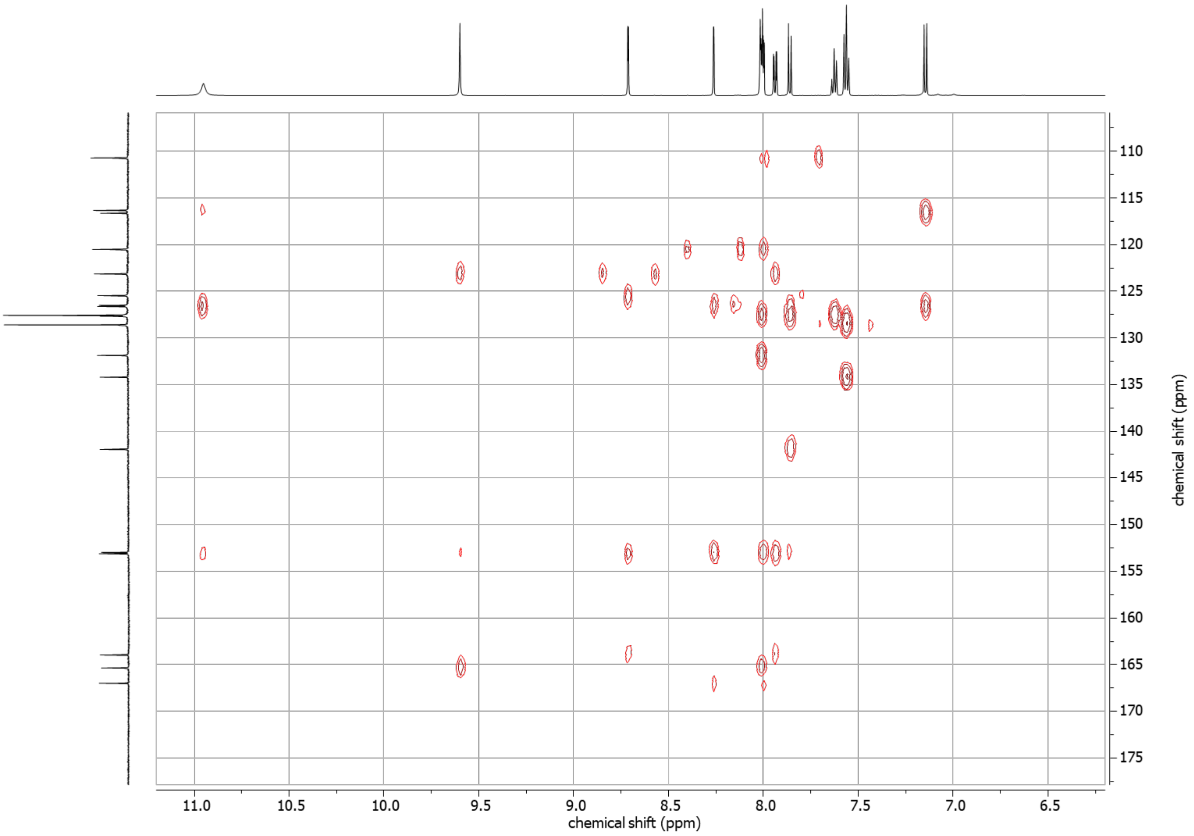

Figure S 62. HMBC spectrum (DMSO-d_6) of closoxazole F (**15***).

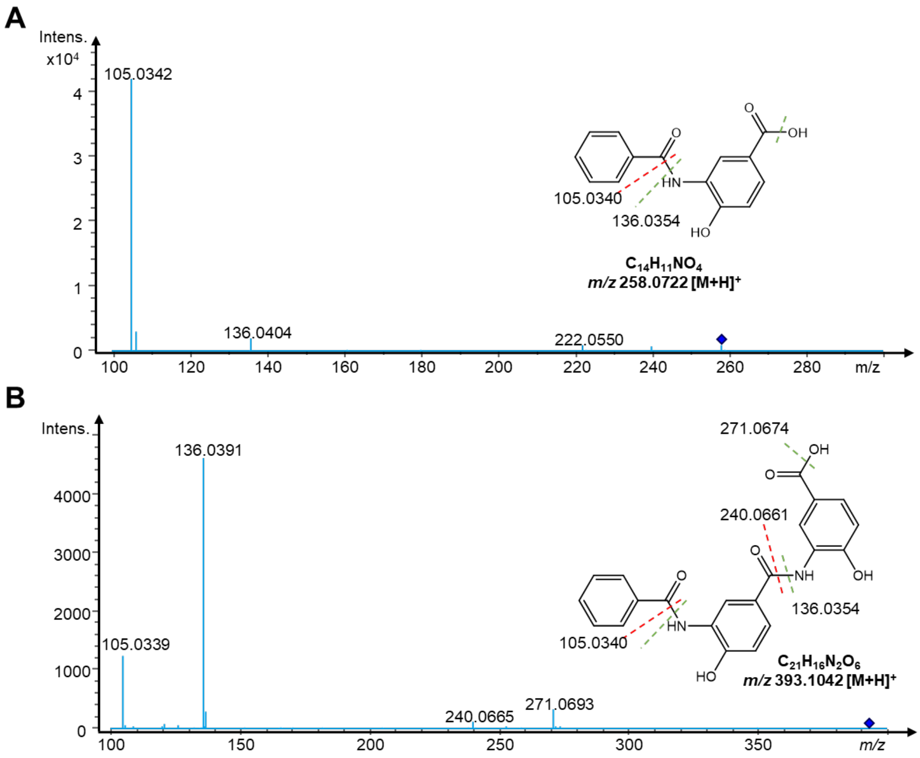

Figure S 63. LC-MS/MS fragmentation patterns of (A) **16** (*m/z* 258.07) and (B) **18** (*m/z* 393.11) produced in in vitro reaction using the enzymes PfxA, PfxB, and PfxC and the substrates 3,4-AHBA and BA.
